# Analysis of plasma‐derived small extracellular vesicle characteristics and microRNA cargo following exercise‐induced skeletal muscle damage in men

**DOI:** 10.14814/phy2.70056

**Published:** 2024-09-20

**Authors:** Jason Lovett, Rhys S. McColl, Peter Durcan, Ivan Vechetti, Kathryn H. Myburgh

**Affiliations:** ^1^ Department of Physiological Sciences Stellenbosch University Stellenbosch South Africa; ^2^ Department of Nutrition and Health Sciences University of Nebraska‐Lincoln Lincoln Nebraska USA

**Keywords:** damage, eccentric exercise, extracellular vesicles, microRNA, muscle

## Abstract

Extracellular vesicle (EV) cargo is known to change in response to stimuli such as muscle damage. This study aimed to assess particle size, concentration and microRNA (miR) content within small EV‐enriched separations prepared from human blood taken before and after unaccustomed eccentric‐biased exercise‐induced muscle damage. Nine male volunteers underwent plyometric jumping and downhill running, with blood samples taken at baseline, 2, and 24 h post‐exercise. EVs were separated using size exclusion chromatography (SEC) and their characteristics evaluated by nanoparticle tracking. No changes in EV size or concentration were seen following the muscle‐damaging exercise. Small RNA sequencing identified 240 miRs to be consistently present within the EVs. RT‐qPCR analysis was performed: specifically, for known muscle‐enriched/important miRs, including miR‐1, −206, −133a, −133b, −31, −208b, −451a, −486 and − 499 and the immune‐important miR‐21, −146a and − 155. Notably, none of the immune‐important miRs within the EVs tested changed in response to the muscle damage. Of the muscle‐associated miRs tested, only the levels of miR‐31‐5p were seen to change with decreased levels at 24 h compared to baseline and 2 h, indicating involvement in the damage response. These findings shed light on the dynamic role of EV miRs in response to exercise‐induced muscle damage.

## 1 INTRODUCTION

Skeletal muscle is central to force production and is subjected to various forms of damage related to physical activity (Hamilton et al., 2015), trauma, and muscle pathologies such as cachexia‐induced muscle wasting (Corona et al., 2016) and myositis (Kamperman et al., 2022). Like most internal organs, skeletal muscle releases several types of particles into circulation, many of which being released at increased rates during and shortly after exercise (Chaar et al., 2011; Neufer et al., 2015; Frühbeis et al., 2015; Whitham et al., 2018). Extracellular vesicles (EVs), which include exosomes, microvesicles, and apoptotic bodies, are a major component of the particles released by cells (Anand et al., 2021; Ståhl et al., 2019). In vitro studies have previously confirmed that skeletal muscle is a significant source of plasma‐derived EVs (Forterre et al., 2014; Guescini et al., 2010; Rome et al., 2019).

Various EV subtypes have been characterized based on specific features, such as the subcellular origin (e.g., endosomal or plasma membrane), biophysical characteristics (e.g., density and size), and even the separation techniques (Konoshenko et al., 2018; Ståhl et al., 2019). Small EVs, smaller than 200 nm, are produced and selectively packaged within the endosome; unlike the larger apoptotic bodies and microvesicles which bud from the cell‐membrane and have a different cargo profile (Jia et al., 2022; Veziroglu & Mias, 2020). Small EVs represent the main category of EVs released by cells as well as the most widely studied with regard to signaling and pathological conditions (Jia et al., 2022).

MicroRNAs (miRs) are a group of small non‐coding RNAs (generally 19–25 nucleotides long) involved in various post‐transcriptional mechanisms influencing mRNA translation for example, the transient repression of mRNA translation into protein (O'Brien et al., 2018) or destabilization of mRNA leading to its degradation (Wilczynska & Bushell, 2015). In this way, miRs are important in fine‐tuning many cellular processes (Wilczynska & Bushell, 2015). In circulation, miRs can be free or contained within/bound to extracellular particles (EPs) that include EVs as well as RNA‐binding protein complexes consisting of molecules such as ApoA1 and Argonaut 2 (Hunter et al., 2008; Zaborowski et al., 2015). These particles provide miRs with protection from rapid breakdown within the bloodstream (Hunter et al., 2008; Zaborowski et al., 2015).

A number of skeletal muscle‐enriched miRs (enriched by 20‐fold vs. other tissues), termed myomiRs, have been identified and include miR‐1, 133a, 133b, and 206 (McCarthy, 2008; Rome et al., 2019). The number of known myomiRs has subsequently expanded to include miR‐208a‐3p, −208b‐3p, −486‐5p and ‐499a‐5p (Aoi et al., 2013; Small et al., 2010). A number of other miRs are still known to be important regulators of skeletal muscle growth e.g., miR‐489 and ‐31, which have been shown to promote satellite cell quiescence (Cheung et al., 2012; Crist et al., 2012), as well as miR‐27a, a miR known to promote satellite cell proliferation (Huang et al., 2012). Additionally, miR‐451a is highly upregulated in older Rhesus monkey muscle while in C2C12 myoblasts, miR‐451a levels have been shown to drop during differentiation (Munk et al., 2019).

Over the past decade, research has begun to establish changes in circulating miRs (free and encapsulated), following various exercise modalities, including short and longer endurance exercise, with or without weight bearing (Baggish et al., 2014; Nielsen et al., 2014; Russell et al., 2013; Uhlemann et al., 2014) and resistance exercise (Annibalini et al., 2019; Sawada et al., 2013; Xhuti et al., 2023). Less information is available on miR release following muscle‐damaging exercise (Banzet et al., 2013), although establishing a role for specific miRs as markers of muscle damage may have applications in other scenarios of muscle pathology (Koutsoulidou & Phylactou, 2020). For example, Banzet et al. (2013) found that concentric exercise increased the levels of miR‐181b and miR‐214 in human plasma, while eccentric exercise resulted in increases in miR‐1, miR‐133a, miR‐133b, and miR‐208b levels between 2 and 24 h post‐exercise; it was not determined whether these miRs were encapsulated, within vesicles or bound to protein, or free within the plasma samples tested.

Despite the increased interest regarding circulating EVs and miRs, few studies have focused on EV size and number as well as miR content in response to muscle‐damaging exercise. Guescini et al. (2015) reported that 40 min of high‐intensity aerobic exercise increased the levels of miR‐133b and ‐181a‐5p within circulating EVs (Guescini et al., 2015). Annibalini et al. (2019) reported elevated circulating EV concentrations and EV‐associated miR‐206 and miR‐146a levels 2 h after flywheel‐based resistance exercise. Specific biological responses to muscle‐damaging exercise suggest that EV or miR responses or miRs contained in EVs may be different at later time points, more typical of times when circulating markers of muscle damage are elevated (Banzet et al., 2013; Brancaccio et al., 2010). Although eccentrically‐biased plyometric jumping exercise followed by downhill running did not result in statistically significant changes in EV size and number following exercise, qPCR analysis showed a decrease in the levels of miR‐31 at 24 h post‐exercise (Lovett et al., 2018). The immune system is involved in the early phases of muscle damage and regeneration (Tidball, 2017) thus also warranting investigation of immune‐important miRs in EV cargo at various time points after muscle‐damaging exercise.

As such, decreases or elevations of muscle‐important miRs in circulation should be considered with regard to muscle regeneration after damage. Furthermore, it appears that the type of exercise is a significant factor in directing the miR response and should be considered when designing and analyzing the results from such studies. Additionally, immune‐important miRs that might be relevant to muscle damage and regeneration have been identified, for example, miR‐21‐5p (Yang et al., 2018) and miR‐155‐5p (Corsten et al., 2012).

The aims of this study were firstly, to assess any changes in size and number of small EVs in separations prepared from plasma following unaccustomed eccentrically‐biased exercise. Secondly, total small RNA sequencing was used to determine the number of different miRs present within the EV‐enriched preparations. Finally, the study aimed to determine by use of qPCR, whether the levels of muscle‐ and immune‐response‐associated EV miRs changed following exercise.

## 2 METHODS

### 2.1 Ethics statement

This study was approved by Stellenbosch University's Health Research Ethics Committee and was carried out in accordance with the guidelines of the South African Medical Research Council and the Declaration of Helsinki.

### 2.2 Participants

Nine healthy, untrained males between the ages of 18 and 30 volunteered for this study. Each volunteer gave written informed consent. Exclusion criteria for participants included regular exercise exceeding 2 bouts per week, participation in professional sport, smoking, or the use of anti‐inflammatory medication within 3 months preceding the study. Participants were required to be in good health (with no chronic illnesses) and should not have participated in any strenuous exercise in the week preceding the study, or throughout the duration of study. All blood draws were taken with the participants in a fasted state that is, at least 8 h since last meal.

### 2.3 Exercise regimen

Each participant performed two consecutive bouts of eccentrically‐biased, muscle‐damaging exercise. This consisted of a combined regimen of both plyometric jumping and downhill running (DHR), modified from similar protocols previously used in our lab (Lovett et al., 2018; Macaluso et al., 2012).

Fasted participants were guided through a 5 min warm‐up focused to the quadricep muscles. Following this, all participants completed 10 sets of 10 plyometric jumps at 90% of their maximum achievable jump height, with a 1 min standing interval between sets. Upon completion of the plyometric jumping regimen, a 5 min rest period was given before commencement with DHR, which consisted of 5 × 3 min sets of DHR at 10 km/h, at a 10% decline, and were given a 2 min standing interval between sets. DHR was stopped if the rating of perceived exertion reached the maximum value of 20 on the Borg scale: 6–20 (Borg, 1982). Perceived muscle soreness (PMP) was assessed using a Delayed onset muscle soreness (DOMS) scale of 1 to 10. A score of 1 signified no pain, and a score of 10 signified extreme, or unbearable pain. Scores were recorded at baseline and at 2, 6, 24, 48 and 72 h post‐exercise.

### 2.4 Blood sampling

Approximately 25 mL of whole blood was drawn from the antecubital vein by a qualified phlebotomist at baseline, and at 2 (acute phase reaction) and 24 h (ongoing inflammatory and repair processes) post‐exercise. The 2 and 24 h time points were chosen as markers of muscle damage, such as IL‐6, creatine kinase, LDH, IGF‐1, and myoglobin as well as circulating miRs have been shown to become elevated within this time frame following eccentric exercise (Annibalini et al., 2019; Banzet et al., 2013; Brancaccio et al., 2010); we therefore chose to asses EV dynamics and content during this period. The 2 and 24‐h time points represent the acute phase and the ongoing inflammatory and repair phase respectively. Furthermore, Banzet et al. (2013) have shown that there is little change in the levels of circulating miRs immediately after muscle damaging exercise; seemingly due to a delayed response to the stimuli. Participants were instructed to not take any anti‐inflammatory or analgesic medication following the exercise protocol and before the final blood draw. Blood was collected into EDTA‐coated (plasma) or heparin‐containing tubes (serum). All blood draws were taken in the fasted state. Blood plasma was isolated from whole blood by centrifugation at 2000×*g* for 10 min at 4°C and immediately frozen at −80°C for subsequent analysis (i.e., SEC isolation, NTA, western blotting, total RNA sequencing, and miR qPCR). Blood serum was collected for serum creatine kinase analysis (analysis done by PathCare, South Africa).

### 2.5 Extracellular vesicle enrichment

Plasma was thawed on ice and centrifuged at 10,000 ×*g* for 10 min to remove larger particles such as apoptotic bodies. Extracellular vesicles were enriched from the clarified plasma using qEVoriginal 70 nm size exclusion columns (Izon Science), according to the manufacturer's protocol. One milliliter of clarified plasma was added to the top of the qEV column, and 12 × 500 μL fractions (F1–F12) of the eluent were collected. qEV columns were continuously topped with PBS, so as to not allow the frit to run dry. Samples were aliquoted and frozen at −80°C.

### 2.6 SDS‐PAGE and western blotting

Prior to the running of the SDS‐PAGE, the individual qEV SEC fractions (500 μL) were concentrated using 10 kDa centrifugal concentrator columns (Amicon® Ultra, cat. UFC501096). The concentrated qEV SEC fractions were mixed with five times RIPA buffer (one part RIPA with four parts sample), containing a protease inhibitor cocktail (Roche, cat. 04693116001) and PhosSTOP (Sigma Aldrich, cat. 4906845001) and were then placed on ice for 1 h. After incubation on ice for 1 h, the protein samples were sonicated (3×5 s bursts at amplitude 3, Misonix Ultrasonic Liquid sonicator, model S‐2000‐010) to ensure lysis of contained vesicles. Before loading onto the gels, the protein lysates were reduced by mixing 1:5 (buffer: sample) in sample treatment buffer (containing 5% mercaptoethanol) and heated to 95°C for 5 min on a heating block. The acrylamide gels (4% stacking, 15% resolving) were cast containing 0.5% TCE (Merck, cat. 8,086,100,100) to allow for UV activation and visualization of protein bands using a Bio‐rad Chemidoc MP gel system (Bio‐Rad, cat. 170–8280). The gels were run at 90 v for 2 h.

Gels were transferred onto 2 μm nitrocellulose membranes (Bio‐Rad, cat. 162‐0112) using a semi‐dry Transblot® Turbo™ transfer apparatus (Bio‐Rad, cat. 1704150EDU) at 25 V, 2.5 mA for 20 min. Following transfer, the blots were blocked in 5% (v/v) fat‐free milk in tris‐buffered saline containing 0.1% (*v*/*v*) Tween 20 (TBST) (Sigma Aldrich, cat. P1379‐500 mL). All antibodies used during labeling were diluted in TBST containing 1% (*w*/*v*) bovine serum albumin (BSA). The blots were treated either anti‐α‐sarcoglycan (1/500, Santa Cruz, sc‐271321), anti‐Alix (1/500, Santa Cruz, sc‐53,540), anti‐ApoA1 (1/500, sc‐376818), anti‐calnexin (1/500, sc‐23954), anti‐CD9 (1/500, Santa Cruz, cat. sc‐13118), anti‐TSG101 (1/500, Santa Cruz, cat. sc‐7964) primary antibodies for 4 h at room temperature on a tube roller. The blots were then washed with TBST and incubated with a 1/10000 dilution of an HRP‐linked, anti‐mouse secondary antibody (Cell Signalling, cat. 7074S) for 1 h at room temperature on a roller. The blots were then washed thoroughly and visualized using the SuperSignal™ West Femto Maximum Sensitivity Substrate (Thermo Scientific, cat.34095) and a Bio‐Rad Chemidoc MP gel system.

### 2.7 Scanning transmission electron microscopy

Two hundred mesh carbon‐coated copper TEM grids were incubated on 10 μL droplets of EV‐enriched preparation aliquots for 10 min. Grids were then washed with dH_2_O and carefully dabbed onto Whatman paper. Following this, grids were incubated on 10 μL of freshly filtered 2% Uranyl acetate Zero for contrast (Agar Scientific). EV‐loaded grids were viewed using a Zeiss Merlin field emission scanning electron microscope (Zeiss) at 20 kV.

### 2.8 Nanoparticle tracking analysis

Vesicle size and number were assessed using nanoparticle tracking analysis (NTA), with the NanoSight LM10 (Malvern Instruments). The qEV SEC fractions were diluted in PBS (25X—200X) for a suitable number of particles per frame (± 35), and three 30‐s videos were used to determine mean particle numbers per mL and mean particle diameters. The detection threshold was kept constant for all samples, at 6.

### 2.9 Small RNA sequencing

Total RNA was isolated from pooled EV fractions 7 to 10 (F7–F10) using the Norgen Plasma/Serum RNA Purification Mini Kit (Norgen Biotek: 55000). Prior to sequencing the quantity of RNA was determined by use of a small RNA Pico chip kit (RNA 6000 Pico kit, Agilent technologies) with analysis being performed in a Bioanalyzer 2100 (Agilent technologies). Next‐generation small RNA sequencing was done on 3 baseline and 3 matched 24‐h post‐exercise samples using an Illumina NextSeq500 (Norgen Biotek), specifying between 15 and 50 nucleotides in length. Post‐processing of the raw miRNA sequencing data was provided by Norgen Biotek; and produced using the excerpt small RNA‐seq Pipeline (v4.6.2) data analysis workflow with miRbase version 21 being used to obtain reference miR sequences.

### 2.10 microRNA qPCR

Total RNA was isolated from the pooled EV‐enriched fractions (qEV SEC fractions 7 to 10) using the Norgen Plasma/Serum RNA Purification Mini kit as per the manufacturer's guidelines (Norgen Biotek: 55000) and reverse transcribed using the TaqMan Advanced miRNA cDNA synthesis kit (Thermo Fisher Scientific, Waltham, MA, United States; A28007). qPCR analysis was achieved using TaqMan Advanced miRNA assays; cel‐miR‐39‐3p (478293_mir), miR‐1‐3p (477820_mir), miR‐21‐5p (477975_mir), miR‐26a‐5p (478293_mir), miR‐31‐5p (478015_mir), miR‐133a‐3p (478511_mir), miR‐133b (480871_mir), miR‐146a‐5p (478399_mir), miR‐155‐5p (483064_mir), miR‐206 (477968_mir), miR‐208b‐5p (477806_mir), miR‐451a (478107_mir), miR‐486‐5p (478128_mir), and miR‐499a‐5p (478561_mir) (all obtained from Thermo Fisher Scientific, Waltham, MA, United States). qPCR analysis was performed in duplicates on a StepOne qPCR thermocycler (Applied Biosystems). As control, 10 pm of the synthetic C. Elegans oligo, cel‐miR‐39 (Sequence: UCACCGGGUGUAAAUCAGCUUG), was added to the isolated total RNA. This sequence does not exist in humans and was used as an exogenous control. All qPCR reactions were normalized to their corresponding cel‐miR‐39 Ct values.

### 2.11 Statistical analysis

All data are represented as means with standard deviations (SD) unless otherwise stated. In figures that represent individual participant values, each colored dot represents a single participant with the color assigned to each being consistent between figures. Data regarding perceived muscle pain, creatine kinase levels, nanoparticle tracking analysis (NTA) readings, and protein concentrations were analyzed by means of one‐way ANOVAs along with the Fisher LSD post‐hoc test using the SPSS software package. For the small RNA sequencing, all displayed read counts per million were first normalized in GraphPad Prism 5 (GraphPad Software Inc., USA) using the Trimmed Mean of M‐values (TMM) method. miR ΔCt values were normalized to the exogenous. When analyzing the qPCR results, the R statistical analysis software was used to show normal distributions as well as to show statistical significance by way of mixed model ANOVAs along with the LSD post‐hoc test. Graphs were made using GraphPad Prism 5, and statistical significance was set at a 5% confidence interval for all statistical tests i.e., *p* < 0.05.

## 3 RESULTS

### 3.1 Participant characteristics

Nine healthy male participants took part in the exercise regime. The average age, height and weight of the participants were 20 years (± 2.5), 178 cm (± 4.5), and 72 kgs (± 5.8) respectively.

### 3.2 Assessment of exercise‐induced, muscle damage

**FIGURE 1:**
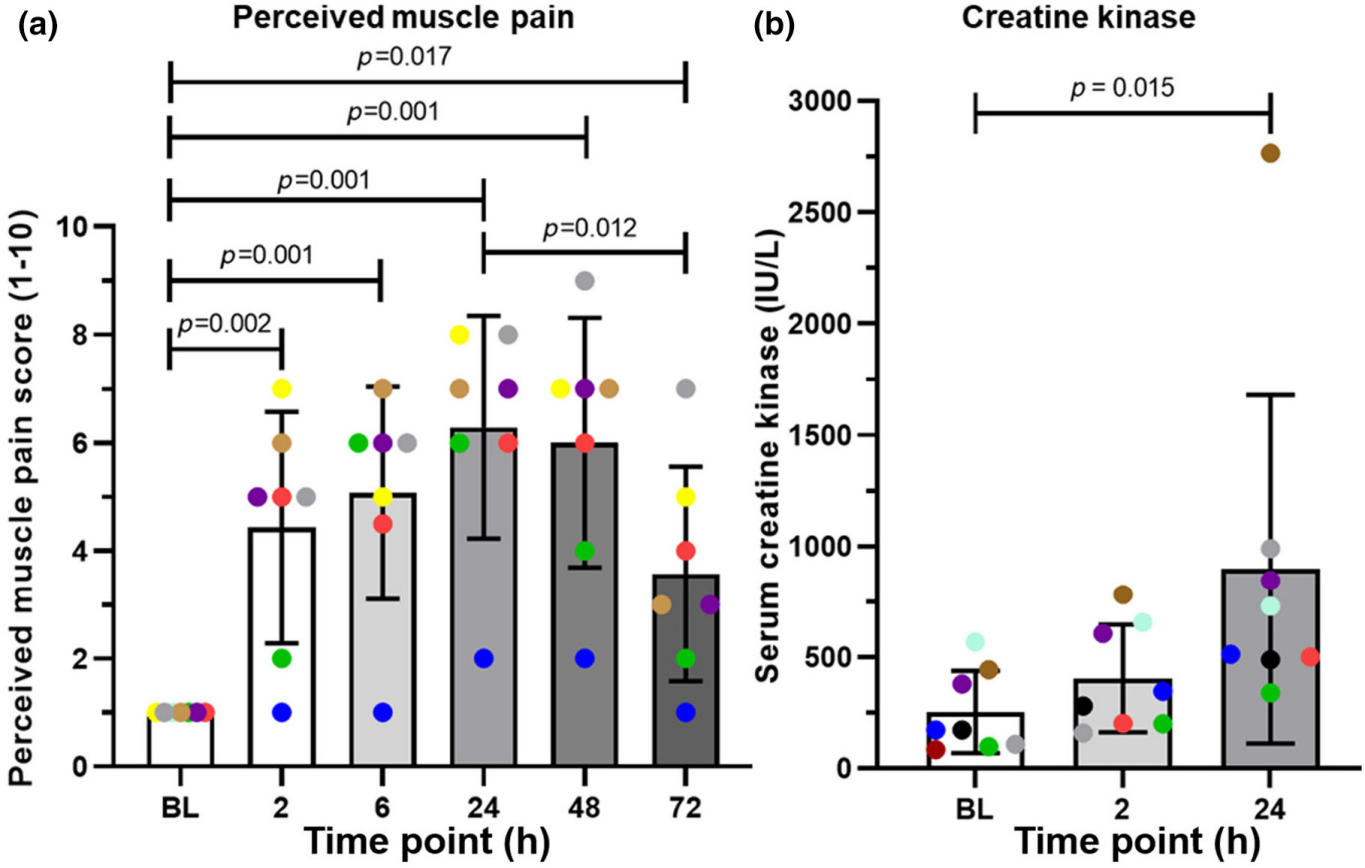
Indirect measures of post‐exercise muscle damage. All data are expressed as means with standard deviations. The colored dots denote individual participants. (a) Perceived muscle pain of the *anterior quadriceps* muscles upon walking, expressed on a scale of 1–10 (*n* = 7). (b) Serum creatine kinase levels before and after muscle‐damaging exercise (*n* = 8). BL = baseline. Statistical analysis was carried out using one‐way ANOVAs along with the LSD post‐hoc test.

All 9 participants were able to complete at 10 × 10 sets of plyometric jumps while only 7 were also able to complete all 5 bouts of DHR. Two of the participants were only able to complete 2 bouts of DHR following the plyometric jumps due to muscle fatigue/pain. Two different indirect indicators of muscle damage were used; perceived muscle pain (PMP) and serum creatine kinase levels (CK). All PMP scores and CK levels are expressed as means with SD values in brackets. PMP scores (baseline score taken to be 1 = no pain) within the first 24 h post‐exercise increased as time progressed (see Figure 1a). All post‐exercise PMP scores were increased relative to the BL PMP score of 1 (± 0) with a peak score of 6.29 (± 1.9) being seen at 24 h post‐exercise (*n* = 7). PMP was still present 72 h following exercise, 3.57 (± 1.8). Serum CK levels, measured in international units/liter (IU/L), are a clinically used indicator of general muscle damage (Baird et al., 2012). A statistically significant increase in CK levels was seen at 24 h, 896 IU/L (± 734) when compared to BL, 253 IU/L (± 172) (*p* = 0.015); a roughly threefold increase (Figure 1b) (*n* = 8).

### 3.3 NTA analysis of qEV SEC separated fractions

The process of qEV SEC has typically been highly successful in the enrichment of EVs with low carry‐over of contaminating particles for example, albumin and HDL. Following further characterization of the prepared fractions, fractions 7 to 12 were seen to be EV enriched.

**FIGURE 2:**
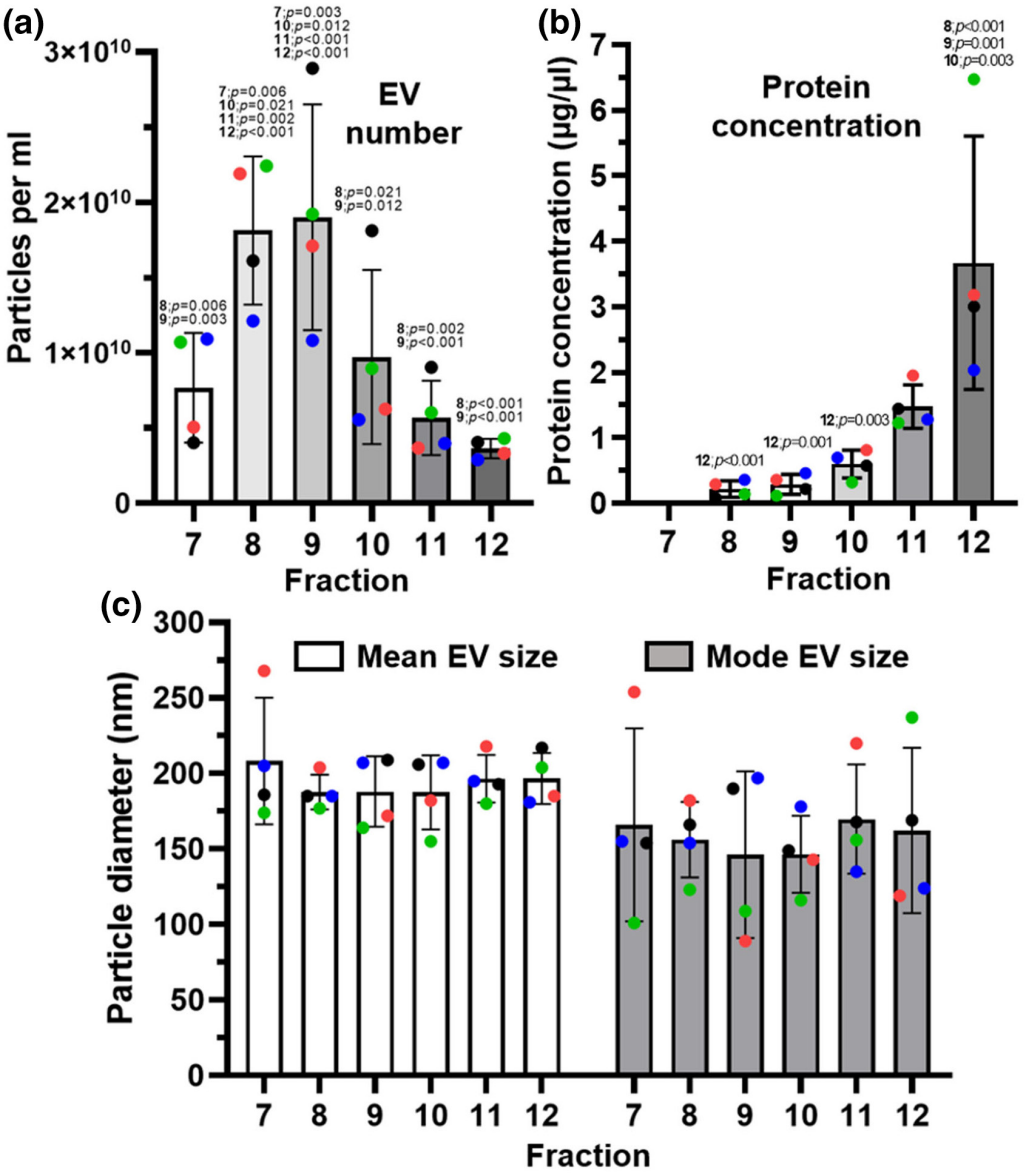
Extracellular vesicle concentration, mean size, mode size as well as protein concentration within qEV SEC separated fractions. All data are expressed showing standard deviations. The colored dots denote individual participants. (a) EV concentrations within individual qEV SEC fractions displayed as particles/mL (*n* = 4). The numbers above the individual bars denote significance. Fractions 7–12 are displayed; fractions 1–6 had no detectable particles. Only particles between 10 and 1000 nm in size are detected/measured by the NTA setup. (b) Protein concentration readings were measured using a BCA assay. Protein levels were measured in SEC fractions 7–12 prepared from plasma samples taken at baseline (BL) (*n* = 4); F7 had undetectable protein levels. (c) Mean and mode EV diameters expressed in nanometers (nm) (*n* = 4). Statistically significant changes were determined by use of one‐way ANOVAs and the LSD post‐hoc test.

To determine which qEV SEC‐separated plasma fractions would be pooled for further analysis, a subset of baseline samples was used to determine the average size and concentration of particles within the individual fractions (*n* = 4). A NanoSight LM10 was used to detect particles between 10 and 1000 nm in diameter. Very few particles were detected in fractions earlier than fraction 7 (F7) and were excluded from further analysis. The majority of small EV‐sized particles were found in fractions 7–10 (Figure 2a). The concentrations of EVs in fractions 7–12 were 0.77×10^10^ (± 0.32 × 10^10^), 1.81×10^10^ (± 0.43 × 10^10^), 1.90*×*10^10^ (± 0.64 × 10^10^), 0.97×10^10^ (± 0.50 × 10^10^), 0.56 × 10^10^ (± 0.21 × 10^10^) and 0.36 × 10^10^ (± 0.056 × 10^10^) respectively (Figure 2a). No statistically significant differences in mean or mode EV diameters were observed between the different fractions (Figure 2c). The average diameters of the EVs were 208 nm (± 36), 188 nm (± 10), 188 nm (± 20), 188 nm (± 21), 197 nm (± 14) and 198 nm (± 15) for fractions 7–12 (Figure 2b). The mode sizes were 170 nm (± 63), 153 nm (± 24), 132 nm (± 47), 146 nm (± 25), 170 nm (± 36), and 160 nm (± 54) for fractions 7–12 (Figure 2b). Protein content was determined by a micro‐BCA assay (Figure 2b). Quantifiable protein was detectable in fractions 8–12 but not in fraction 7. The high levels of protein in fractions 11 and 12 were likely due to the presence of non‐EV‐associated proteins.

### 3.4 Qualitative assessment of protein present in qEV SEC isolated fractions

**FIGURE 3:**
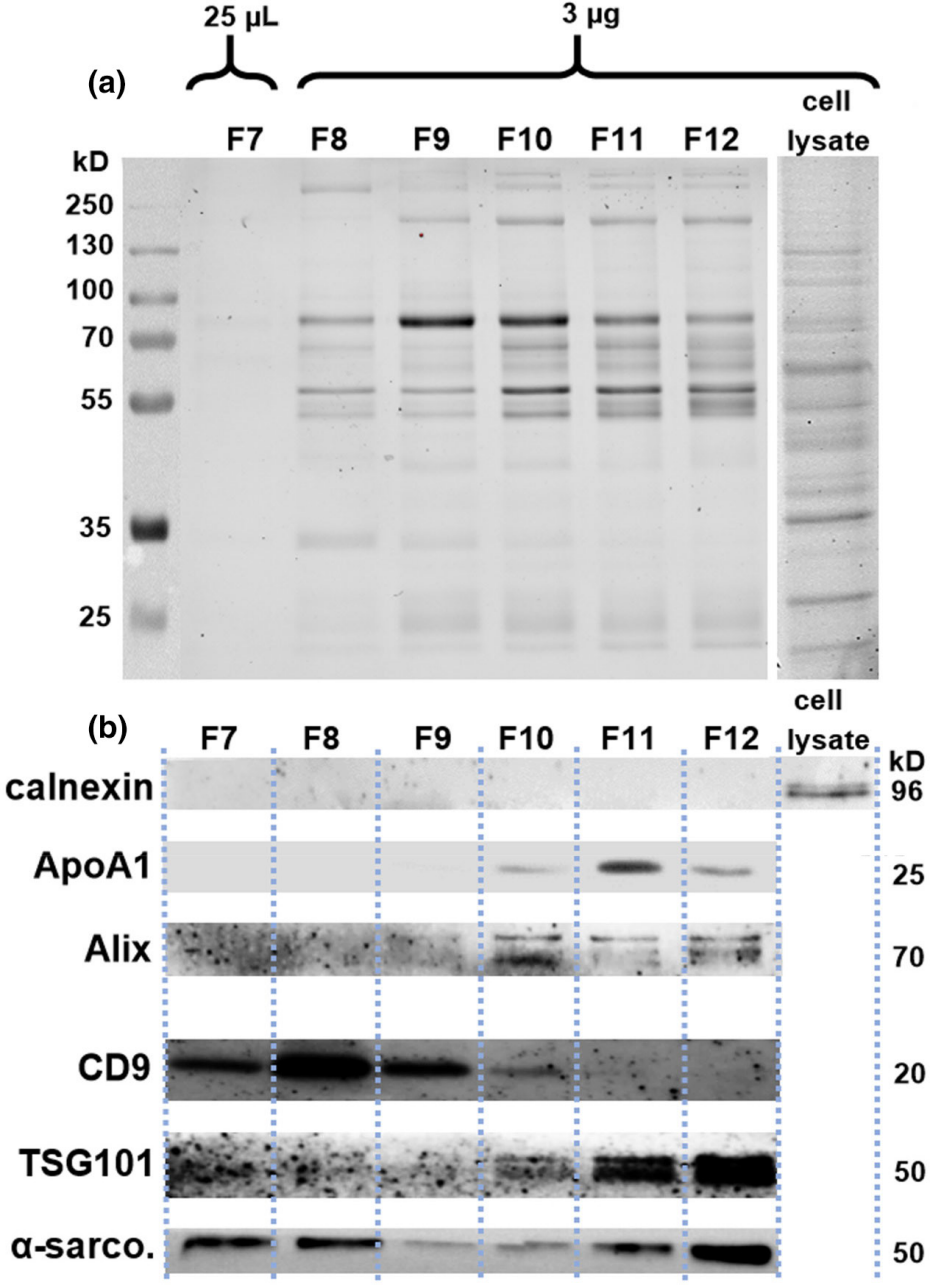
Analysis of potential contaminants as well as EV‐associated proteins within qEV SEC separated fractions. All data are expressed as means with standard deviations. (a) Representative image of a UV‐activated SDS PAGE gel of size exclusion fractionated BL plasma (fractions 7–12). Protein molecular weight ladder in left lane with molecular weights indicated. For fraction 7, a fraction with low protein content, 25 μL of the concentrated fraction was loaded onto the gel. For fractions 8–12, volumes containing 3 μg of protein were loaded. (b) The enrichment/levels of EV‐associated as well as non‐EV‐associated contaminating proteins were determined by western blotting. The cell lysates used for the calnexin blots were prepared from proliferating primary human myoblasts. Due to the limited number of gel lanes for loading, the cell lysates were not run for every antibody tested.

The first few fractions derived from qEV SEC columns are known to contain very low amounts of proteins or to be devoid of proteins altogether, hence protein assessment started at F7. A total of 3 μg of protein from fractions 8–12 was added to each well while a total of 25 μL (max volume) of fraction 7 was analyzed due to the low protein yield in this fraction. Distinct protein bands were visible in the lanes for fractions 8 to 12 (Figure 3a). Major protein bands were present at approximately 25, 55, 65, 80 and 150 kDa in most of the fractions. These bands likely represent abundant plasma proteins such as albumin (± 66 kDa), transferrin (± 79 kDa) and immunoglobulins (± 150 kDa) (Ahmed, 2009). Western blotting was used to determine the levels of both EV‐associated as well as contaminating, non‐EV‐associated proteins (Figure 3b). No calnexin, a marker of cellular contamination, was detected in any of the fractions. A calnexin band was seen in the control cell lysate lane; these lysates were prepared from proliferating primary human myoblasts (PHMs). The lipoprotein ApoA1, a component of RNA‐carrying HDL (Vickers & Michell, 2021), was very faintly detected in fraction 10 with the highest amount being seen in fraction 11 and a moderate amount in fraction 12; for this reason, fractions 11 and 12 were not pooled for further analysis. The EV‐associated molecule Alix (van Niel et al., 2018) was detected in fractions 9–12 with the strongest band seen in fraction 10. CD9, another molecule enriched in EVs (Jankovičová et al., 2020; van Niel et al., 2018), was detected in fractions 7–10 and was particularly enriched in fractions 7, 8 and 9; fractions 8 an 9 having the highest NTA particle counts. TSG101 was detected in fractions 10 to 12. Taken together, CD9 (F7‐10) and Alix/TSG101 (fracs 10–12) appeared to be enriched in different fractions, possibly representing different sub‐populations of EVs; although both markers were seen to overlap in fraction 10. The α‐sarcoglycan molecule has previously been identified as being specific to EVs originating from muscle tissue (Guescini et al., 2015; Madison & Robinson, 2019). We detected α‐sarcoglycan in all of the fractions. Distinct α‐sarcoglycan bands are present in fractions 7 and 8 and may represent protein present within EVs originating from muscle tissue. From the NTA and protein analysis results presented above, it was decided to pool fractions 7–10 for further sequencing and qPCR analysis.

### 3.5 Visualization of EVs: Scanning electron microscopy (SEM)

**FIGURE 4:**
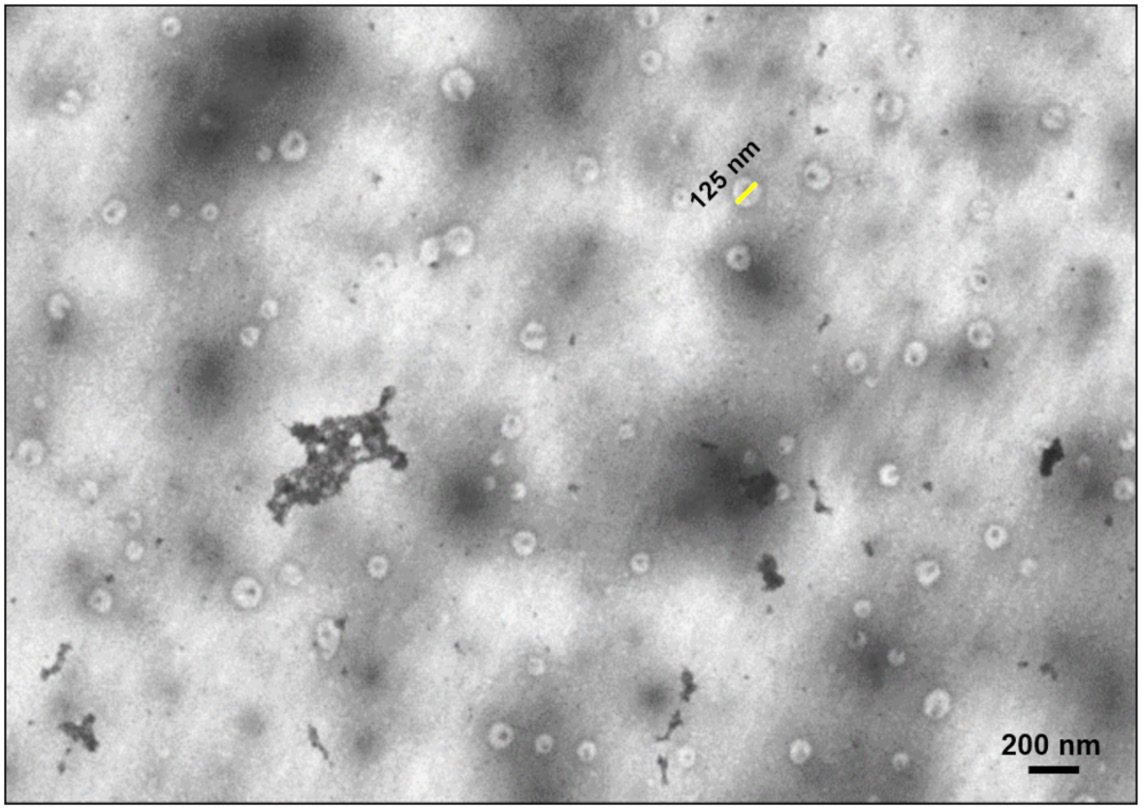
Visualization of qEV SEC enriched EVs using widefield scanning transmission electron microscopy. Extracellular vesicles, derived from qEV size exclusion columns, were visualized in widefield using scanning transmission electron microscopy. This representative image is of eluted fraction 8 obtained from baseline plasma. An example of a suspected extracellular vesicle of approximately 125 nm in diameter is highlighted by the yellow bar. The darker clumps of matter may represent abundant plasma proteins such as albumin and lipoproteins.

Previously published reports using qEV SEC show that fractions enriched with exosome‐sized vesicles (30–200 nm) are mostly free of the larger apoptotic bodies and larger microvesicles (100–1000 nm); membranous particles that bleb from the cell surface and are not known to contain selectively‐packaged cargo in the same way as smaller EVs (30–200 nm) (Frattini et al., 2017; Guescini et al., 2015; Jia et al., 2022; Pratesi et al., 2013). A SEM image of F8 from a random BL sample from the current study is presented in Figure 4; a number of circular particles with sizes corresponding to those of smaller EVs were seen in these images. The dark clumps within the SEM image likely represent abundant plasma‐derived proteins such as albumin and/or lipoproteins.

### 3.6 Effect of muscle‐damaging exercise on the number and diameter of small plasma‐derived EVs

**FIGURE 5:**
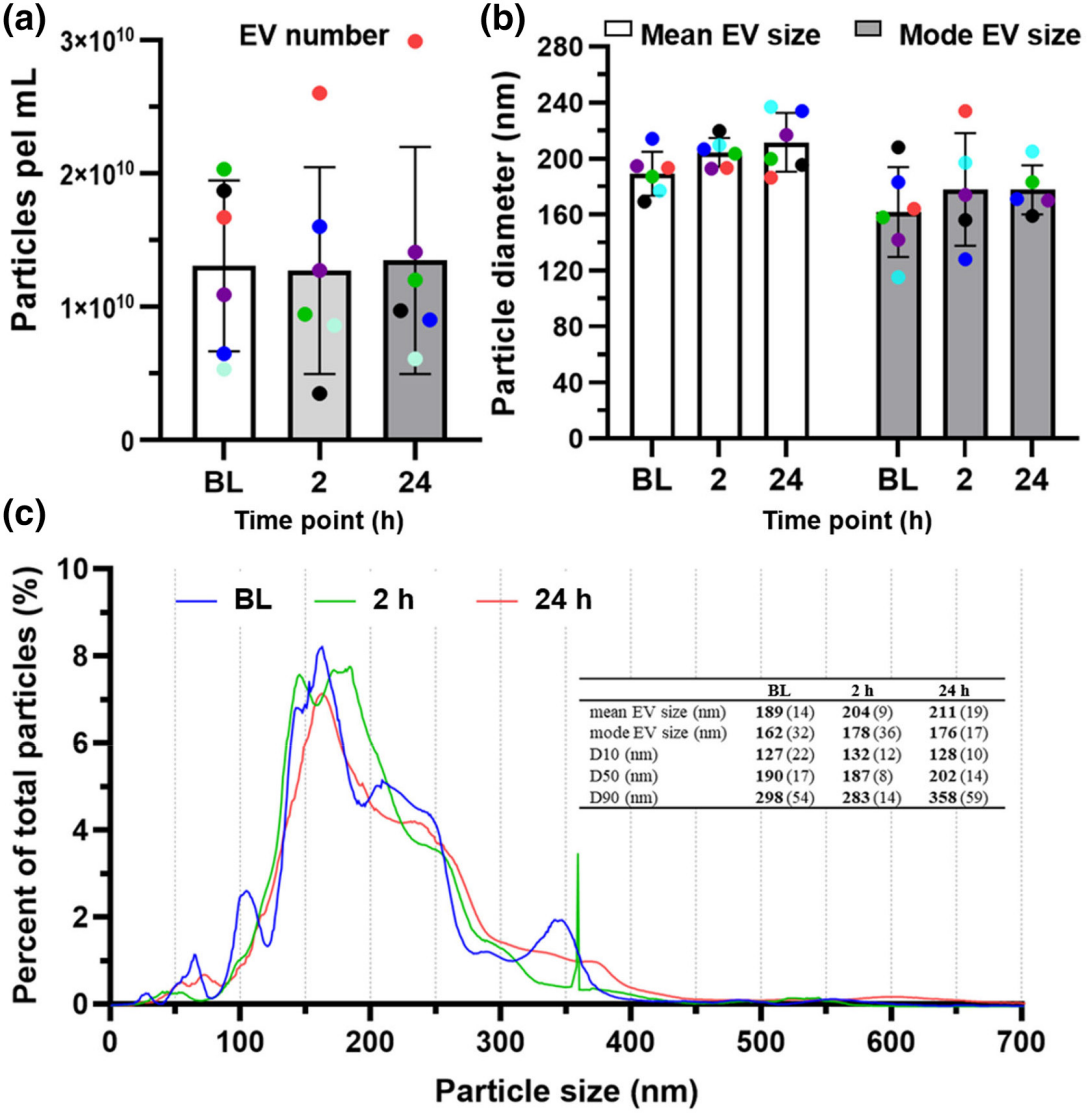
Pooled, plasma‐derived EV concentrations, mean diameters and size distributions following exercise‐induced muscle damage (pooled qEV SEC fractions 7–10). All data are expressed as means with standard deviations. qEV SEC fractions 7–10 were pooled for each time point. The colored dots denote individual participants. (a) Total EV counts within pooled EV‐enriched preparations at BL, 2 and 24 h following muscle‐damaging exercise (*n* = 6). (b) EV‐enriched isolate mean and mode particle sizes (*n* = 6). (c) Particle size distributions. The size distributions are displayed as percent of total particles to account for variability in the number of particles that were isolated from each participant No statistically significant differences in the number or size of the isolated particles were seen when comparing the three time points. Statistical significance was determined by the use of one‐way ANOVAs and the LSD post‐hoc test (*p* < 0.05).

To determine whether the two consecutive bouts of muscle‐damaging exercise had any effect on circulating EV number or size, the pooled samples from 6 participants at BL, 2 and 24 h post exercise were analyzed using NTA (pooled fractions 7–10; *n* = 6). The EV numbers (see Figure 5a) and size (see Figure 5b) are presented for the three time points. No statistically significant changes in pooled EV size or number were observed between any of the time points in response to the muscle damaging exercise. The numbers of EVs at the three time points were; 1.31 × 10^10^ (± 0.59 × 10^10^) (BL), 1.27 × 10^10^ (± 0.71 × 10^10^) (2 h) and 1.35 × 10^10^ (± 0.78 × 10^10^) (24 h). The average diameters of the enriched EVs were; 189 nm (± 14) (BL), 204 nm (± 9) (2 h) and 211 nm (± 19) (24 h) while the mode diameters were determined to be 162 nm (± 32) (BL), 178 nm (± 36) (2 h) and 176 nm (± 17) (24 h). The normalized size distribution plots (Figure 5c) reveal no obvious shift in these distributions following muscle damage. These size distributions were calculated as the percent of the total number of EVs to account for the variation in total particle counts between the participants' samples.

### 3.7 RNA sequencing of total RNA associated with pooled qEV SEC fractions 7–10

Given the emphasis in the literature on circulating EV RNA cargo, we first sought to gain a general idea of the number of small RNAs present within the small EV‐enriched preparations. To achieve this, small RNA sequencing was applied to a subset of participant samples at baseline and 24 h post muscle‐damaging exercise (*n* = 3): fractions 7–10 were pooled prior to microRNA isolation. The RNA sequencing protocol used in this study focused on sequencing fragments of 15 to 50 nucleotides in size. In this study, we focused on the small, non‐coding microRNA.

### 3.8 Total RNA concentration of pooled qEV SEC fractions 7–10 from pre‐ and post‐exercise samples

No statistically significant difference in total RNA concentration was found between the BL and 24 h post‐exercise samples (286 pg/μL ± 32 and 360 pg/μL ± 6, *p* = 0.196). The same was seen for the miR concentrations (114 pg/μL ± 27 vs. 154 pg/μL ± 8, *p* = 0.347).

### 3.9 Total number of miRs identified using RNA sequencing analysis

Using a detection limit of at least one miR read per million, 362 different miRs were detected across the six samples tested (three samples at baseline and three at the 24 h time point). When considering miRs detected in all samples and with at least 5 miR reads per million, 240 miRs were identified. The quality of the RNA samples used was determined by Norgen Biotek prior to sequencing; this analysis is available in the sequencing report attached as an additional file.

### 3.10 The 25 most abundant miRs within the EV‐enriched fractions

The most abundant miR in all samples was miR‐26a‐5p with roughly 200,000 and 260,000 reads per million at BL and 24 h post‐exercise, respectively. Due to the relatively small number of samples sequenced (*n* = 3 for each time point), no conclusions were drawn with regards to fold‐changes. The top 25 most abundant miRs detected in BL and 24 h samples are displayed in Table 1.

**TABLE 1:** The 25 most abundant miRs within the pooled EV‐enriched fractions (Banzet et al., 2013; Barnes et al., 2022; Bettio et al., 2023; Borg, 1982) at baseline and 24 h post‐exercise according to small RNA sequencing.

A	miR	BL—reads per million	24 h—reads per million
1(1)	**miR‐26a‐5p**	205,383 (21104)	261,005 (25812)
2(2)	miR‐191‐5p	83,922 (2939)	98,855 (7613)
3(4)	miR‐423‐5p	61,178 (2277)	57,328 (10172)
4(6)	**miR‐486‐5p**	60,400 (11786)	42,356 (7981)
5(3)	let‐7f‐5p	54,708 (18 15)	70,509 (10043)
6(5)	miR‐151b	49,298 (2234)	56,779 (5469)
7(7)	**miR‐146a‐5p**	39,978 (4245)	40,212 (4549)
8(8)	miR‐30d‐5p	26,219 (3240)	31,309 (2442)
9(10)	**miR‐21‐5p**	20,500 (4527)	23,320 (6328)
10(9)	let‐7a‐5p	18,168 (1728)	24,582 (3166)
11(12)	miR‐125a‐5p	16,903 (4157)	18,702 (3999)
12(11)	miR‐92a‐3p	14,895 (1089)	18,813 (721)
13(13)	miR‐22‐3p	9519 (19)	8142 (232)
14(17)	miR‐185‐5p	4893 (161)	3672 (77)
15(14)	miR‐30c‐5p	4556 (374)	5985 (197)
16(15)	let‐7d‐5p	3722 (147)	4421 (279)
17(16)	miR‐126‐5p	3454 (594)	4411 (696)
18(19)	miR‐186‐5p	2959 (162)	2173 (233)
19(23)	miR‐24‐3p	2030 (4)	1893 (53)
20(18)	miR‐199a‐3p	1814 (177)	2216 (190)
21(21)	miR‐151a‐5p	1695 (93)	1973 (9)
22(25)	miR‐1301‐3p	1694 (150)	1604 (157)
23(24)	miR‐584‐5p	1627 (142)	1839 (122)
24(22)	miR‐23b‐3p	1570 (229)	1933 (248)
25(20)	let‐7e‐5p	1514 (211)	2013 (278)

*Note*: The miRS have been arranged in descending order according to reads per million (column A) (*n* = 3 for each time point). The number in brackets in column A shows the order of the miR, according to read count, at the 24 h post‐exercise time point. Reads per million displayed as means (SD). The bold, underlined miRs were also analyzed in the qPCR section of the paper. miRs in red are known to regulate muscle function while miRs in blue are linked to immune responses.

### 3.11 qPCR analysis of specific miRs following exercise‐induced muscle damage

**FIGURE 6:**
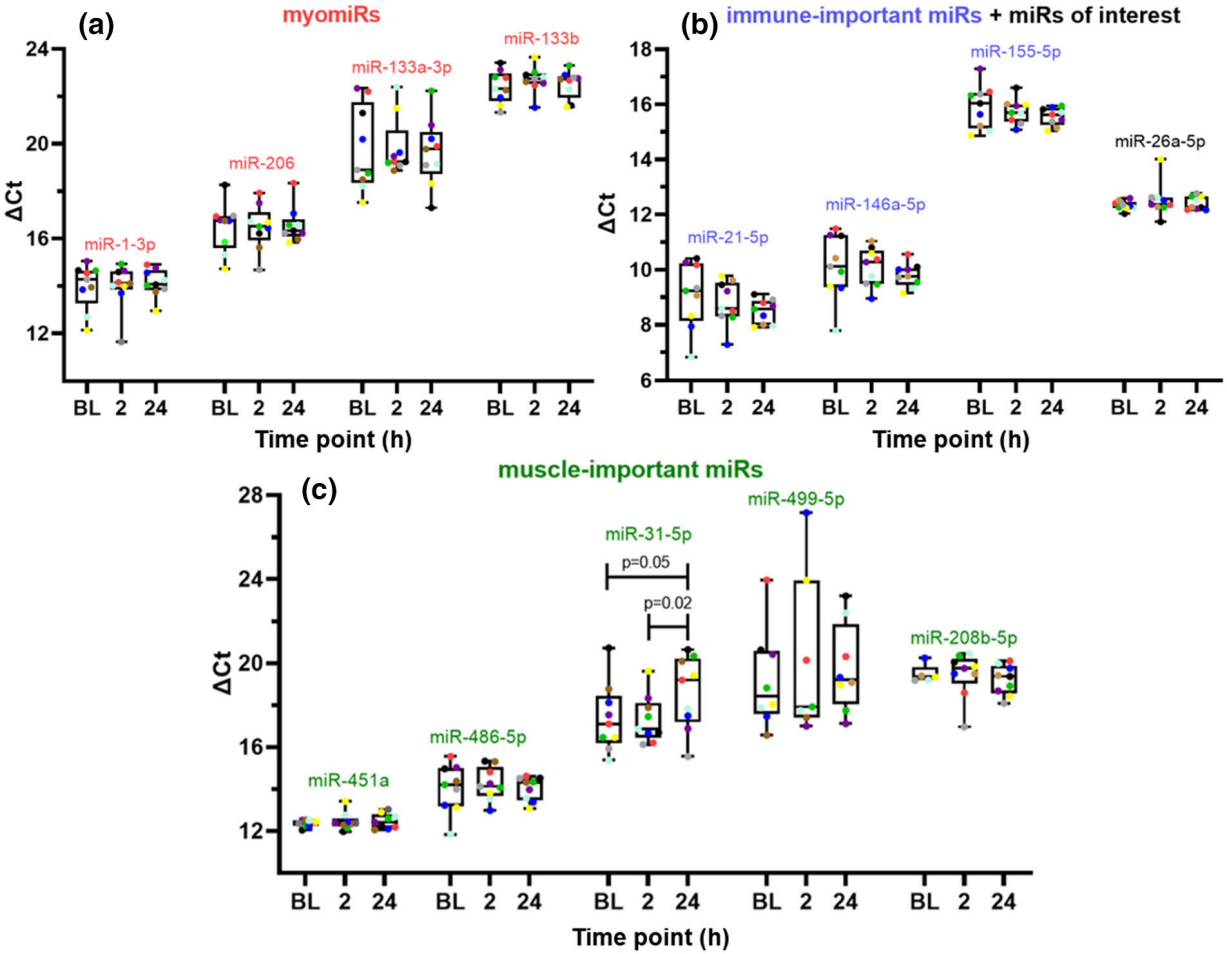
qPCR analysis of selected myomiR, muscle‐important, and immune‐important miR cargo within EV‐enriched preparations post exercise. Minimum to maximum box and whisker plots for miR ΔCt value; *n* = 9 for each miR except miR‐499‐5p (*n* = 8). (a) miRs in red are known myomiRs. (b) Green miRs are known to be muscle‐important. (c) miRs in blue are immune‐important. Statistically significant changes were determined by use of one‐way ANOVAs and the LSD post‐hoc test.

Following the small RNA sequencing, qPCR was used to more substantially determine the levels of 13 miRs of interest within the pooled EV‐enriched fractions (Banzet et al., 2013; Barnes et al., 2022; Bettio et al., 2023; Borg, 1982) from all nine participants and looking at each time point (BL, 2 and 24 h) (Figure 6) (*n* = 9). These 13 miRs included; 4 myomiRs (miR‐1‐3p, −206, −133a‐3p and ‐133b), 5 muscle‐important miRs (miR‐486‐5p, −31‐5p, −499‐5p, −451a and −208b‐5p), 3 immune‐important miRs (miR‐21‐5p, −146a‐5p and miR‐155‐5p) as well as miR‐26a‐5p. miR‐26a‐5p was analyzed as it was by far the most abundant miR detected by the small RNA sequencing. Of the miRs tested using qPCR, only miR‐31‐5p displayed a statistically significant change following exercise (Figure 6). ΔCt for miR‐31‐5p at the 24‐h time point, 18.60 (± 1.65), displayed a statistically significant increase when compared to the BL, 17.38 (± 1.54), and 2‐h time points, 17.31 (± 1.07). This means that total miR‐31‐5p levels decreased following exercise; with decreases of −0.57 (*p* = 0.05) and − 0.59‐fold (*p* = 0.02) when compared to BL and 2 h respectively (calculated using 2^−ΔΔCt^).

## 4 DISCUSSION

The main aim of this study was to determine if the miRs associated with plasma‐derived EVs change in response to skeletal muscle damage in healthy humans following a dynamic eccentric exercise intervention. Several miRs associated with skeletal muscle tissue and the immune cell response to tissue injury were analyzed by use of qPCR. Small RNA sequencing was also used to develop a more comprehensive understanding of the miRs associated with the EVs pre‐ and post‐muscle damage.

Indirect evidence for skeletal muscle damage was established for the eccentric exercise model by determining increases in serum CK levels and PMP scores following the exercise protocol. Although direct evidence of damage was not assessed, Macaluso et al. (2012) have visualized disruption of sarcomere ultrastructure following an acute bout of plyometric jumping. The number of plyometric jumps in their protocol was the same as was used here (10 sets of 10 jumps). The current study protocol involved plyometric jumping followed by bouts of DHR. DHR alone was shown by van de Vyver and Myburgh (2012) to result in a large peak in serum CK, with a concomitant increase in satellite cell activation 24 h post‐exercise (van de Vyver & Myburgh, 2012). From these data, it was concluded that the model of muscle‐damaging exercise induced by two consecutive eccentrically‐biased exercise protocols was sufficient to induce muscle damage.

When EV number is assessed immediately after performance of an aerobically based exercise intervention a significant increase in small EV number has been reported, which returns to baseline levels 90 min post‐exercise (Frühbeis et al., 2015). In the current investigation, blood draw time points were chosen in accordance with concentration changes of circulating molecules which have been investigated on a regular basis, such as CK, Mb, IL‐6 and which have been related to intramuscular events (Peake et al., 2005; van de Vyver & Myburgh, 2012). The 2 h. post‐exercise time point was chosen in accordance with elevation in the inflammatory cytokine, IL‐6, from a similar DHR protocol (van de Vyver & Myburgh, 2012), as well as a reported 2‐fold increase in EVs following maximal resistance exercise in samples taken 2 h post‐exercise (Annibalini et al., 2019). Similarly, the 24 h time point was chosen as this has been shown to be when the largest CK response occurs (Peake et al., 2005). Furthermore, the rate of satellite cell activation in response to muscle damage starts to climb in muscle tissue 24 h after a similar DHR regimen to our own (van de Vyver & Myburgh, 2012). According to our results, circulating EV size distribution and concentration did not change at 2 or 24 h post‐exercise (Figure 5c), a finding similar to that by Rigamonti et al. (2020).

Frühbeis et al. (2015) reported on small EVs after two types of endurance exercise, performed by the same volunteers. In that study the small EVs were isolated using differential ultracentrifugation and size assessed using nanoparticle tracking analysis. The means for plasma small EV diameters were between 100 and 130 nm after cycling or 140 to 190 nm after running (with time points immediately after exercise, at 90 and 360 min post‐exercise). In contrast, the exercise modality of the current study was eccentric muscle‐damaging exercise. Xhuti et al. (2023) reported small EV sizes prepared from blood samples obtained at rest; young and old volunteers did not differ for small EV sizes. These volunteers reported for blood sampling after overnight fasting. Similar to the current study, that study used size exclusion chromatography for the preparation of the EV‐enriched isolates. Unlike the current study, that study included an extra ultracentrifugation step after SEC. The volunteers in the current study also reported to the laboratory in the fasted state in the early morning. At rest, the mean particle diameters were 8% lower than at 2‐h post‐exercise, with the mode 10% different, although not statistically different. This is perhaps not surprising, given that the methodology for concentrating small EVs is inherently selective for size, thus narrowing the possibility to detect changes induced by exercise, without employing other methods. Despite the somewhat higher mean and mode for particle diameters found in the current study compared to the two studies mentioned above, the characterization of specific proteins (Alix, CD9 and TSG101) revealed the presence of these EV‐enriched molecules as well as almost very low levels of ApoA1 in the pooled fractions. The tetraspanin, CD9, was more enriched in the lower fractions, than Alix and TSG101 which are related to biogenesis. It has been noted previously in a number of studies that EV protein enrichment does not correspond precisely to the same fractions (Barnes et al., 2022; Crescitelli et al., 2020; Willms et al., 2016).

In our study, small RNA sequencing of the EV cargo revealed the following general results: (1) there are a wide range of miRs present within the EVs (±360 different miRs detected) with 240 of these short sequences being detected across all EV samples (six samples sequenced; *n* = 3, baseline and 24 h. time points), (2) miR‐26a‐5p was the most abundant miR by far, constituting roughly 23% of all reads when averaging the six samples (Table 1). According to the Human miRNA Tissue Atlas (Ludwig et al., 2016), miR‐26a‐5p is highly expressed in bone, brain tissue, muscle, the spinal cord and in the skin of humans. Findings by Dey et al. (2012) suggest that miR‐26a (not the mature miR‐26a‐5p sequence specifically) promotes the differentiation of mouse myoblasts in vivo by targeting the transcription factors Smad1 and Smad4.

Of the 8, muscle‐important assessed by qPCR (Figure 6), only miR‐31‐5p displayed a statistically significant change following exercise with 0.57 and 0.59‐fold decreases at 24 h post‐exercise when compared to the BL and 2 h time points. Despite not fitting the classic definition of being a myomiR that is, existing ≥20‐fold abundance in skeletal muscle when compared to other tissues, miR‐31‐5p has been reported as being involved in the regulation of skeletal muscle tissue (Crist et al., 2012). Indeed, with regard to myogenesis, Crist et al. (2012) showed that miR‐31‐5p sequesters the mRNA transcript of Myf5. This was found by in situ hybridization, which showed that both miR‐31 and Myf5 were co‐localized in messenger ribonucleoprotein (mRNP) granules in quiescent satellite cells. miR‐31‐5p causes the transient repression of translation of the Myf5 mRNA transcript into the Myf5 protein. Myf5 is present in skeletal muscle samples and in isolated satellite cells after muscle‐damaging exercise (Nederveen et al., 2019). The premise that miR‐31‐5p is involved in suppression of myogenesis is further supported by Dmitriev et al. (2013) using miR profiling of activated CD56+ myoblasts, these authors showed that miR‐31‐5p decreased and remained decreased through the differentiation process. Yet another study alluded to the involvement of miR‐31‐5p in regeneration, particularly with regard to muscular dystrophy (Nunes et al., 2021).

In this study, EDTA was used as the anticoagulant, presenting both advantages and disadvantages when compared to sodium citrate. While EDTA effectively prevents coagulation and typically results in higher yields of EVs, it also increases the risk of platelet activation, potentially leading to the release of additional, unwanted vesicles (Bettio et al., 2023; Buntsma et al., 2022; Lacroix et al., 2013). To mitigate this risk, blood samples were immediately centrifuged to remove platelets. Conversely, sodium citrate offers better preservation of vesicle integrity, though it is less effective as an anticoagulant and may yield lower EV numbers (Bettio et al., 2023; Buntsma et al., 2022; Lacroix et al., 2013).

Although the immune‐important miRs were evident in the EV isolates, no change was seen due to the exercise intervention, despite the known immune cell involvement during early phases post‐exercise and following muscle damage (Kanda et al., 2013; Schlagheck et al., 2020). miR‐21‐5p, 146a‐5p and 155‐5p were chosen due to their known involvement with immune cells (Eigsti et al., 2014). Specifically, miR‐21‐5p and miR‐155‐5p have been shown to target the mRNA of transcription factors important in monocyte differentiation (Schmeier et al., 2009), thereby inhibiting this process. The timing of immune events after damage, may only become apparent with a longer post‐exercise study time course when compared to myomiRs and skeletal muscle‐important miRs.

## 5 CONCLUSIONS

Systemic intercellular communication is not a new concept. A plethora of information on the dynamics of circulating hormones, cytokines and peptides is available for a host of different states in vivo. However, there remains a significant gap in our understanding of EV and miR dynamics, particularly in response to exercise‐induced muscle damage. This study addresses this gap, offering valuable insights and identifying several miRs of interest related to skeletal muscle damage.

## AUTHOR CONTRIBUTIONS

J. L. and K. H. M. conceptualized and designed the study. J. L. and R. S. M. carried out the labwork, analyzed the data and produced the text and figures for the manuscript. P. D. and I. V. provided their insights regarding the results as well as suggested revisions to the final manuscript. All the authors read, revised and approved the final version of the manuscript.

## FUNDING INFORMATION

This study was funded by a South African National Research Foundation (NRF) Grant and NRF Scholarships: Running expenses were funded by the SARChI grant (UID 98565); K.H.M. Research Chair is funded by DST/NRF (UID 98565); J.L. was funded by an NRF‐DAAD PhD scholarship; R.S.M. was funded by an NRF grant‐holder linked PhD scholarship.

## CONFLICT OF INTEREST STATEMENT

The authors have no conflict of interest to declare.

## ETHICS STATEMENT

This study was approved by Stellenbosch University's Health Research Ethics Committee (Study reference: S17/03/061) and was carried out in accordance with the guidelines of the South African Medical Research Council and the Declaration of Helsinki.

## Supporting information


**Data S1:** Supporting Information.

**Data S2:** Supporting Information.

**Figure S1:** Supporting Information.

## Data Availability

Additional supporting information can be found online in the Supporting Information section at the end of this article and/or via contact with the corresponding author (khm@sun.ac.za, KHM).
