# Supplementary material for: Analysis of plasma‐derived small extracellular vesicle characteristics and microRNA cargo following exercise‐induced skeletal muscle damage in men
Source: Physiol Rep. 2024 Sep 20;12(18):e70056. doi: 10.14814/phy2.70056 (PMC11415274; doi:10.14814/phy2.70056)
Supplement: Supplementary file 1 — Data S1: Supporting Information. [file PHY2-12-e70056-s002.pdf]

SERVICE REPORT

Small RNA NGS Profiling Data Report

SV18362

SERVICE REPORT

Customer: Professor Kathy Myburgh
Company/Institute: Stellenbosch University
Date: February 25, 2020

Performed by:
Norgen Biotek Corp.
3430 Schmon Pkwy, Thorold, ON
Canada L2V 4Y6

NORGEN BIOTEK CORP.

TABLE OF CONTENTS

Report Summary 3
Small RNA-Seq Data Analysis Workflow Used 4
Sources of Small RNA Reference Sequences 4
Downloading Your Files 4
Structure of Files Provided 5
Sample Raw Read File Information 5
Quantification of RNA used for Small RNA-Seq 6
Mapping and Biotype Summary 6
Help and Technical Support 8
Small RNA Analysis (Additional Library Summary) 9

Report Summary

**Client Name**	Professor Kathy Myburgh
**Norgen Order Number**	SV18362
**Number of Samples to Process**	6 Samples
**Sample Type**	Plasma exosomes (Human)
**Sequencing Service Provided**	Small RNA-Seq
**Sequencing Platform**	Illumina NextSeq 500
**Sequencing Platform Reagent**	NextSeq 500/550 High Output Kit v2 (51 Cycles using a 75-Cycle Kit)
**Product Used for Library Preparation**	Norgen Biotek Small RNA Library Prep Kit (Cat. 63600)

**Date and Time of Release of Report:**		February 25, 2020	
**Report Prepared by:**	Dr. Mohamed El-Mogy	**Date:**	February 25, 2020
**Report Approved by:**	Dr. Yousef Haj-Ahmad	**Date:**	February 25, 2020

Small RNA-Seq Data Analysis Workflow Used

exceRpt small RNA-seq Pipeline (v4.6.2)
Link: http://genboree.org/theCommons/projects/exrna-tools-may2014/wiki/Small_RNA-seq_Pipeline

Sources of Small RNA Reference Sequences

Small RNA Species	Reference or Database Sequences Used
miRNAs	miRBase version 21
tRNAs	gtRNAdb
piRNAs	RNAdb
Genome	Gencode version 21 (hg38)

Downloading Your Files

All files pertaining to this service have been uploaded to our servers. These files will be available for up to 1 month after the completion of the project. Please make sure to download all files before then.

To access your files, visit the link below and enter the user name and password provided. Click the Download button to start the download. If you have any issues accessing the files, please contact us for help.

Please note that all fields are case-sensitive.

**Download Link:**	https:\\services.norgenbiotek.com\sv18362
**Username:**	user-sv18362
**Password:**	TD-sP3b*JU
**Expiration:**	May 21, 2020

Structure of Files Provided

Inside the UBS hard drive provided, you will find the following file structure that contains the analysis results.

- Raw Reads
  - Contains compressed original FASTQ file of each sample
- Small RNA Analysis
  - Post Processed Results
    - Contains excerpt-generated summary of read counts of each small RNA species (*.txt format)
    - Mapping summary provided in the file: excerpt_readMappingSummary
  - Intermediate Mapping Files
    - Contains intermediate mapping files of each sample
  - Processed Files for Individual Samples
    - Contains compressed folder of excerpt-generated data files of each sample

Sample Raw Read File Information

Norgen ID	Sample ID	Group ID	Index	Associated FASTQ File Name
SV18362-0001	A	1	RPI19	SV18362-0001_S1_R1_001
SV18362-0002	B	1	RPI20	SV18362-0002_S2_R1_001
SV18362-0003	C	1	RPI21	SV18362-0003_S3_R1_001
SV18362-0004	D	2	RPI22	SV18362-0004_S4_R1_001
SV18362-0005	E	2	RPI23	SV18362-0005_S5_R1_001
SV18362-0006	F	2	RPI24	SV18362-0006_S6_R1_001

Quantification of RNA used for Small RNA-Seq

Sample ID	RNA ID	RNA Conc., pg/uL (Bioanalyzer)
SV18362-0001	SV18362-0001_0001-RNA	240.80
SV18362-0002	SV18362-0002_0002-RNA	268.20
SV18362-0003	SV18362-0003_0003-RNA	348.90
SV18362-0004	SV18362-0004_0004-RNA	370.30
SV18362-0005	SV18362-0005_0005-RNA	359.50
SV18362-0006	SV18362-0006_0006-RNA	348.80

Amount of RNA is in line with observed yield of similar RNA samples. It meets the minimal requirement for library prep and sequencing.

Mapping and Biotype Summary

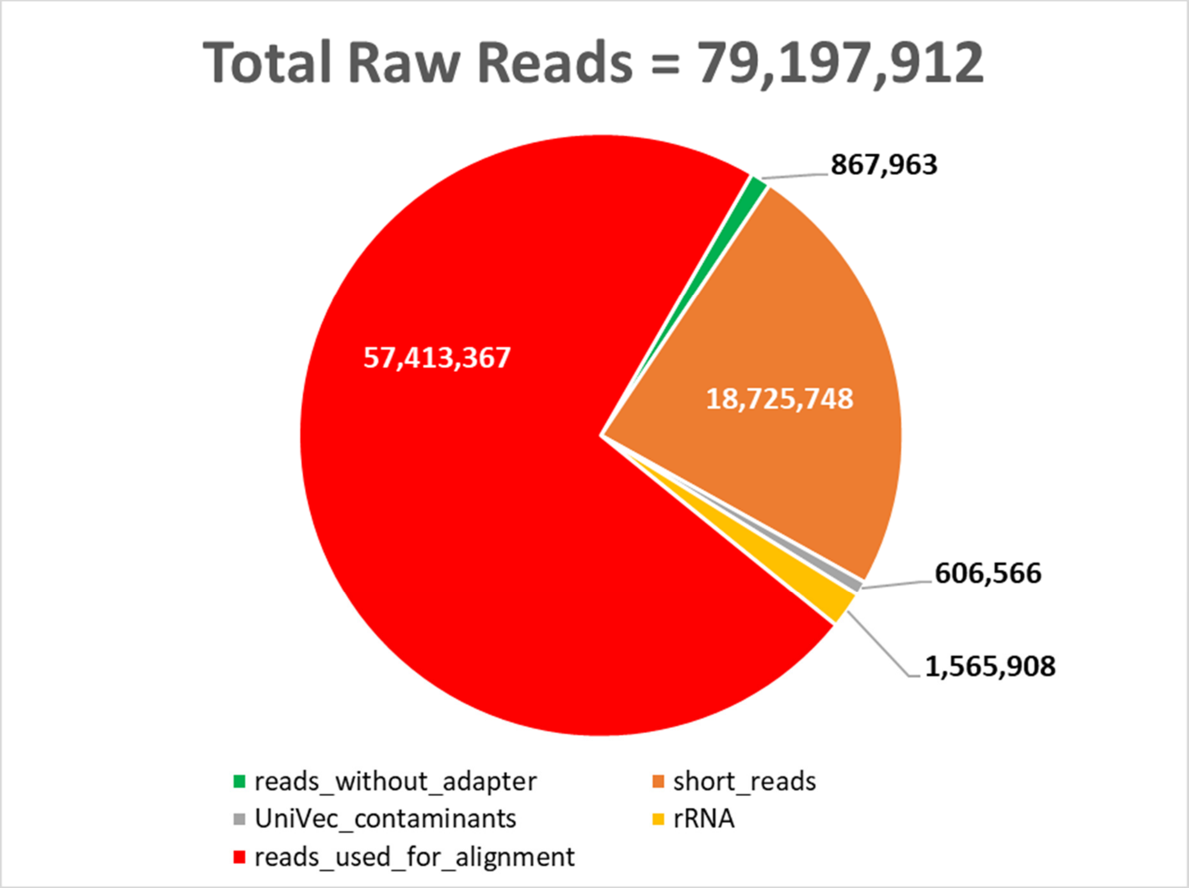

	Total Sample	SV18362-0001	SV18362-0002	SV18362-0003	SV18362-0004	SV18362-0005	SV18362-0006
**Total Raw Read**	**79,197,912**	13,760,245	13,455,935	12,012,745	13,919,922	11,387,899	14,661,166
reads_without_adapter	**867,963**	161,421	143,263	129,836	155,779	117,342	160,322
short_reads	**18,725,748**	4,100,431	4,407,504	2,542,376	2,940,206	1,698,703	3,036,528
passed_initial_qc	**59,585,841**	9,495,386	8,902,503	9,337,638	10,820,635	9,568,910	11,460,769
rRNA	**1,565,908**	265,509	309,824	231,033	204,102	183,986	371,454
**Total Mappable Reads**	**57,413,367**	9,068,732	8,554,326	9,037,792	10,536,304	9,322,044	10,894,169
**reads mapped to genome**	**23,300,123**	2,984,488	1,879,663	3,313,663	4,628,382	5,182,676	5,311,251
**not_mapped_to_genome_or_libs**	**34,113,244**	6,084,244	6,674,663	5,724,129	5,907,922	4,139,368	5,582,918
**miRNA_sense**	**9,869,214**	1,411,469	837,736	1,121,775	2,122,372	2,517,729	1,858,133
miRNA_antisense	**0**	0	0	0	0	0	0
miRNAprecursor_sense	**67,969**	12,227	6,402	7,460	15,860	16,027	9,993
miRNAprecursor_antisense	**148**	1	60	0	32	49	6
tRNA_sense	**522,120**	97,236	48,179	67,666	77,491	121,771	109,777
tRNA_antisense	**228**	37	21	24	64	41	41
piRNA_sense	**2,657,343**	316,927	156,423	457,972	546,864	462,318	716,839
piRNA_antisense	**48**	0	9	6	33	0	0
gencode_sense	**8,266,312**	820,814	510,146	1,340,794	1,522,405	1,809,584	2,262,569
gencode_antisense	**86,672**	12,726	18,904	12,280	17,737	10,969	14,056
circularRNA_sense	**87,718**	14,628	22,350	16,385	13,798	7,817	12,740
circularRNA_antisense	**34,089**	5,846	4,663	6,495	5,606	4,225	7,254

Sample ID	SV18362-0001	SV18362-0002	SV18362-0003	SV18362-0004	SV18362-0005	SV18362-0006
**Number of detected miRNAs at ≥ 5 raw reads**	349	373	376	404	415	366
**Number of detected miRNAs at > 1 raw read**	359	402	399	417	428	379

Help and Technical Support

For any questions or additional help regarding this report, please contact our Technical Support Team between the hours of 9:00 AM and 5:30 PM (Eastern Standard Time) at (905) 227-8848 or Toll Free at 1-866-667-4362. Technical support can also be obtained through email at techsupport@norgenbiotek.com.

Norgen Biotek Corp.
3430 Schmon Parkway, Thorold, ON Canada L2V 4Y6
Copyright © 2020

NORGEN BIOTEK CORP.

Small RNA Analysis (Additional Library Summary)

Read length distribution of sequenced samples based on number of raw reads

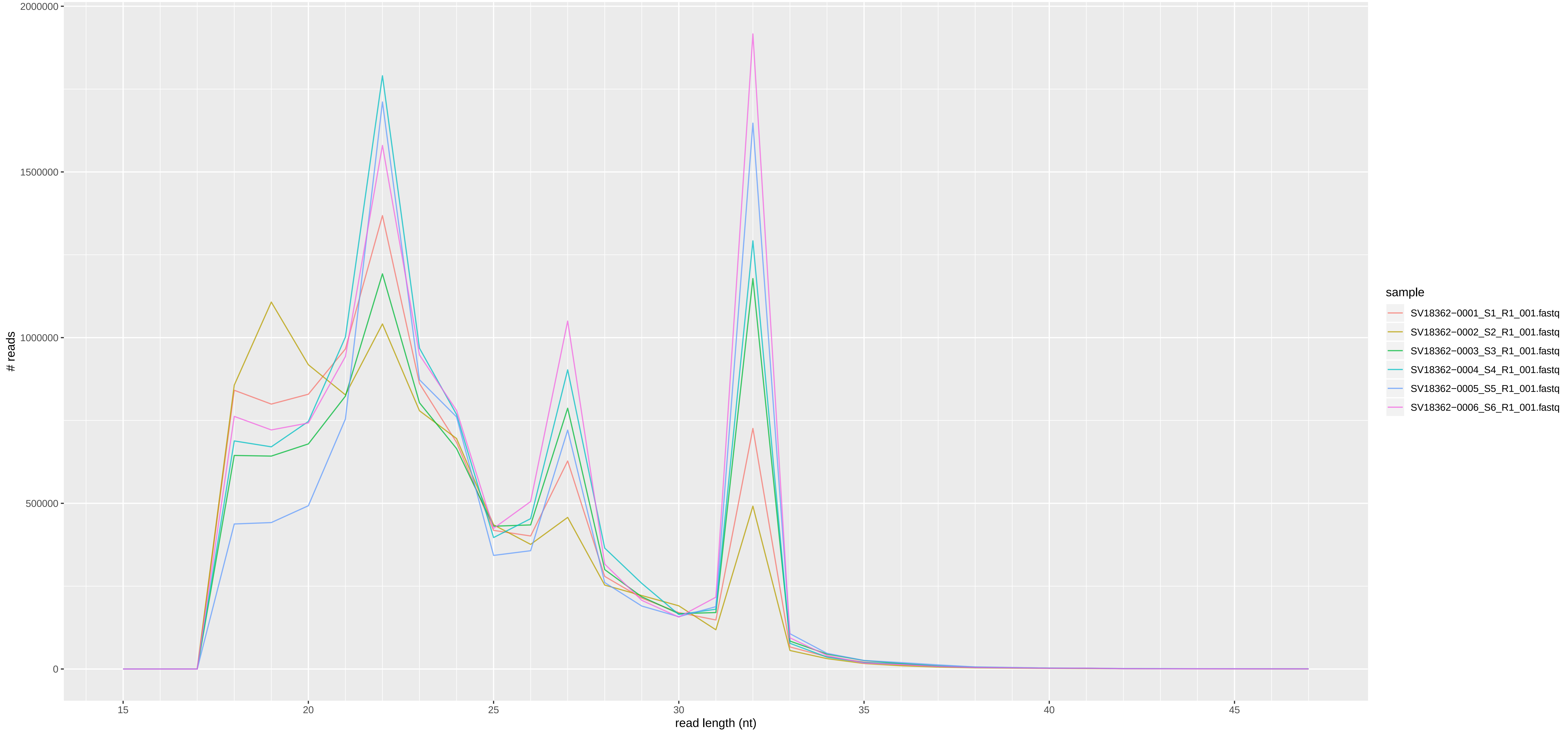

Read length is plotted against number of reads at each specific size (nucleotides) to indicate predominant molecules of nucleotide length corresponding to a specific biotype.

Read length distribution of sequenced samples based on normalized read fractions

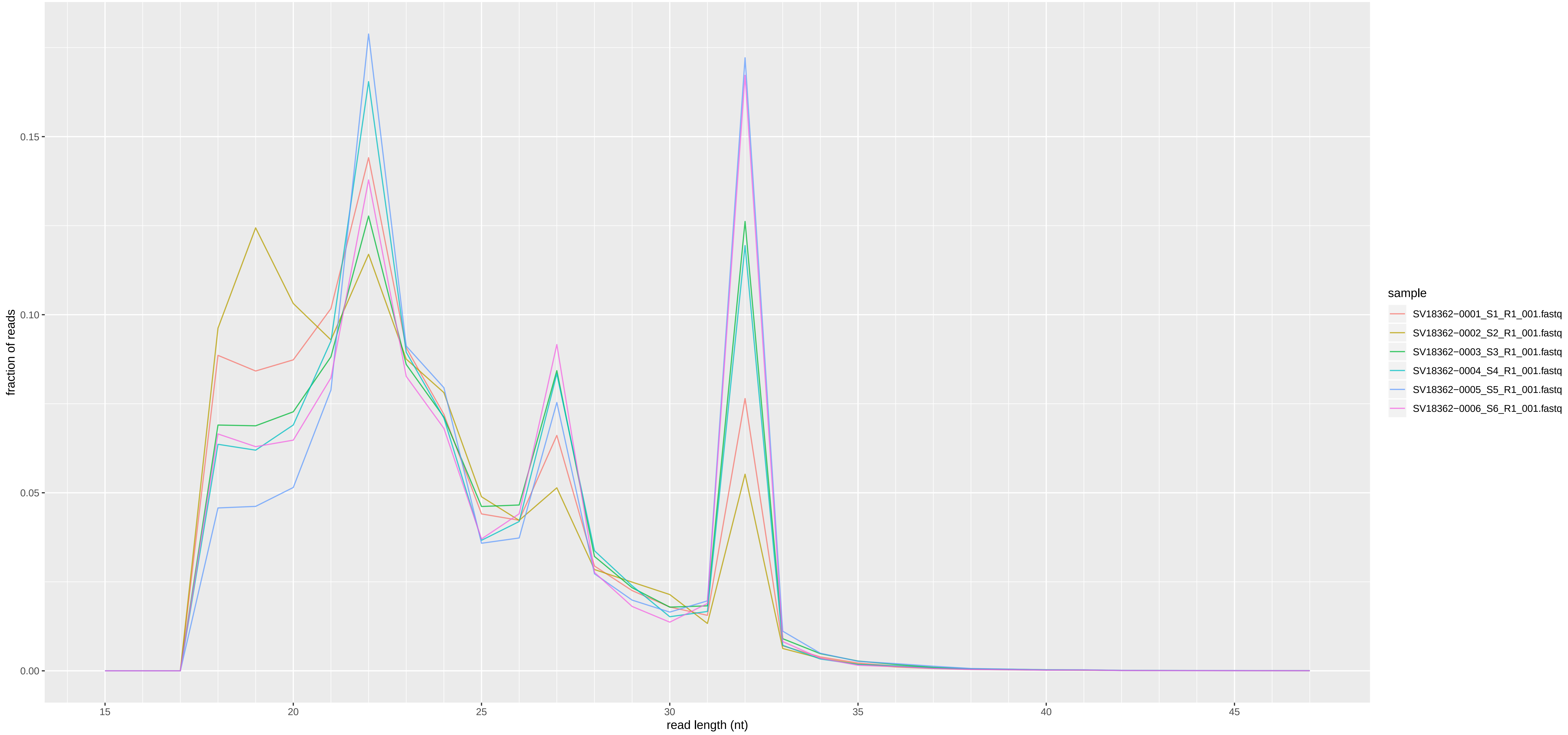

Read length is plotted against normalized reads at each specific size (nucleotides) to indicate predominant molecules of nucleotide length corresponding to a specific biotype.

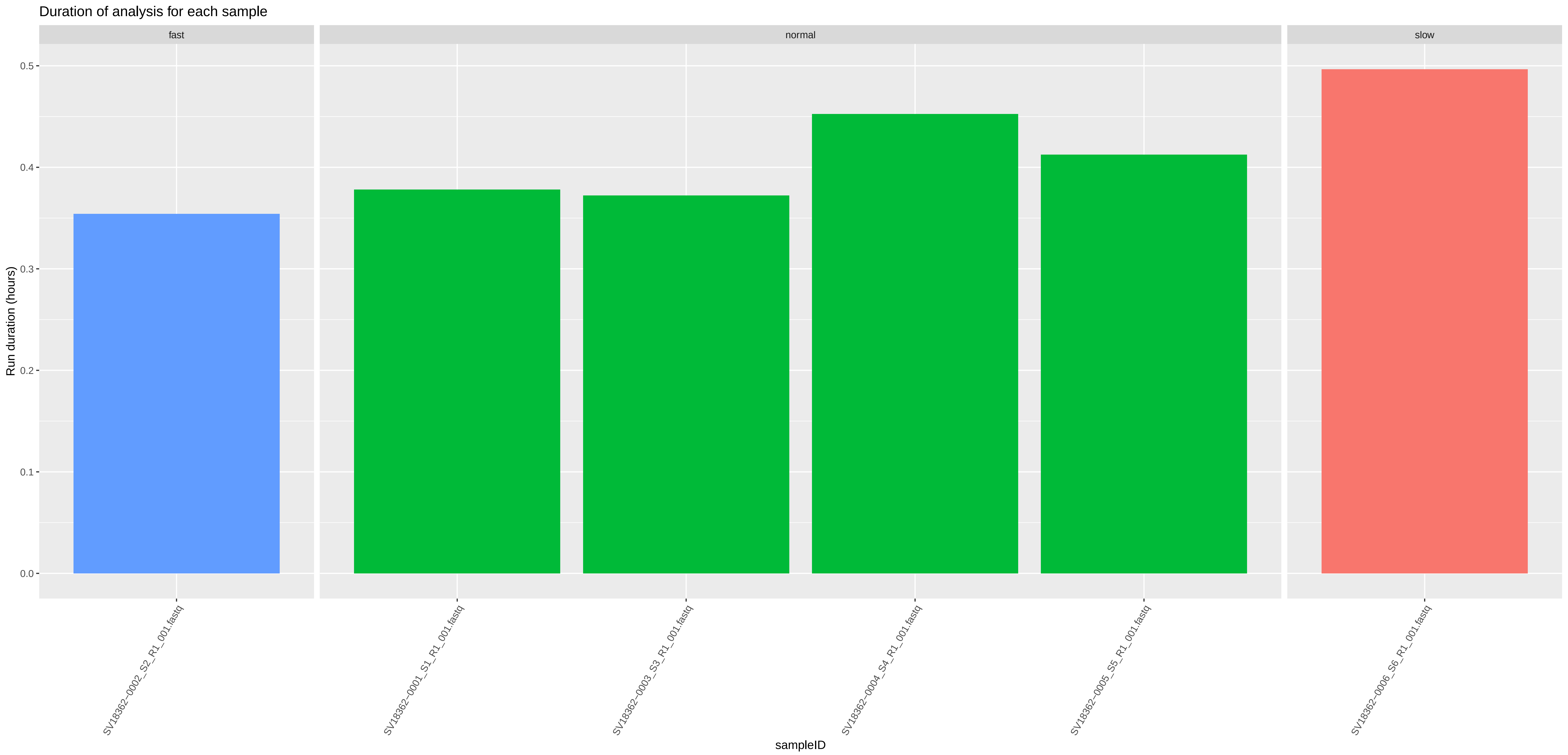

The run duration in hours is illustrated to indicate the time (hours) spent by the analysis pipeline to complete the analysis.

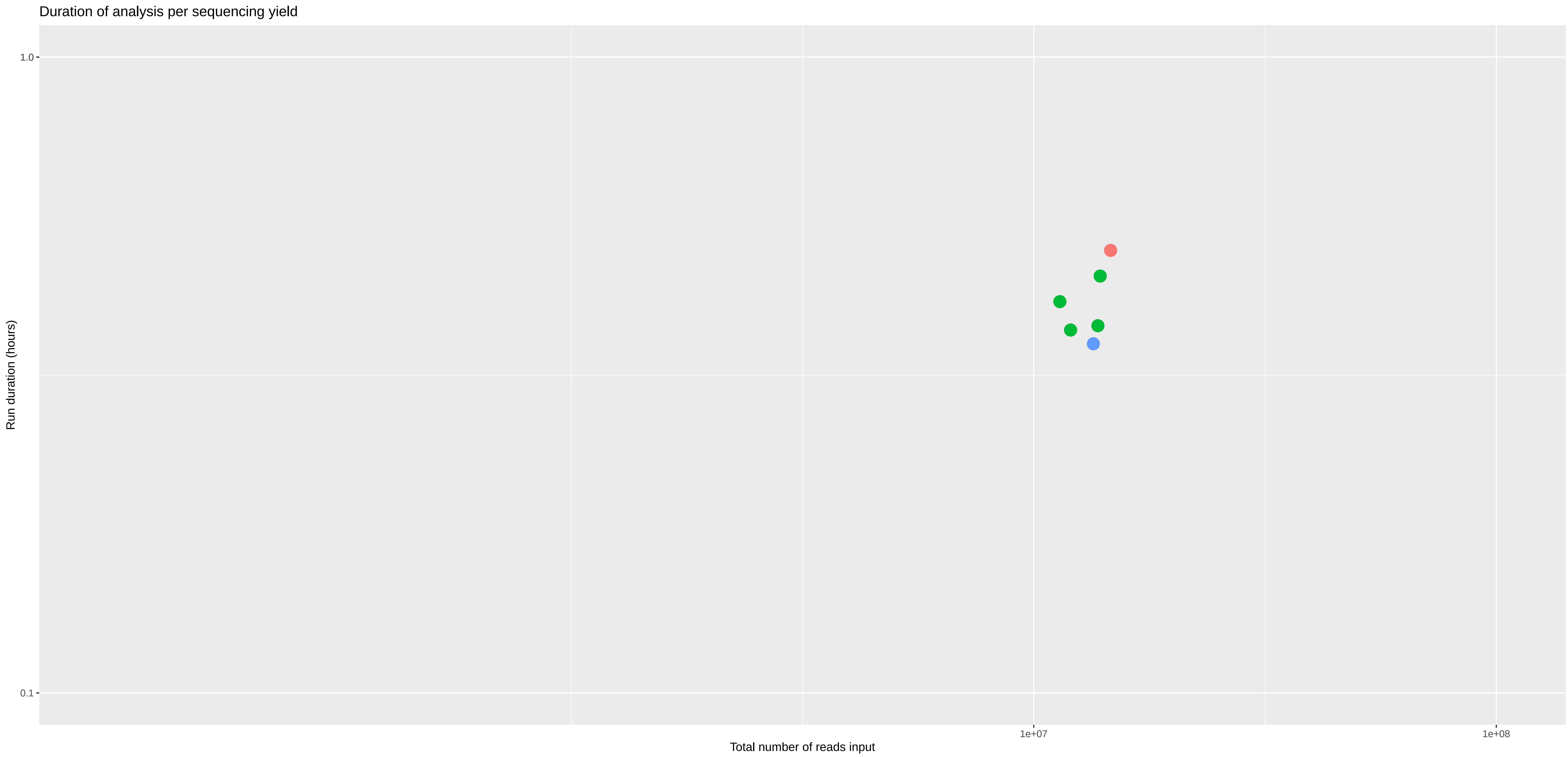

The run duration in hours is illustrated to indicate the time (hours) spent by the analysis pipeline to complete the analysis per total number of reads input.

Library size (all mapped reads)

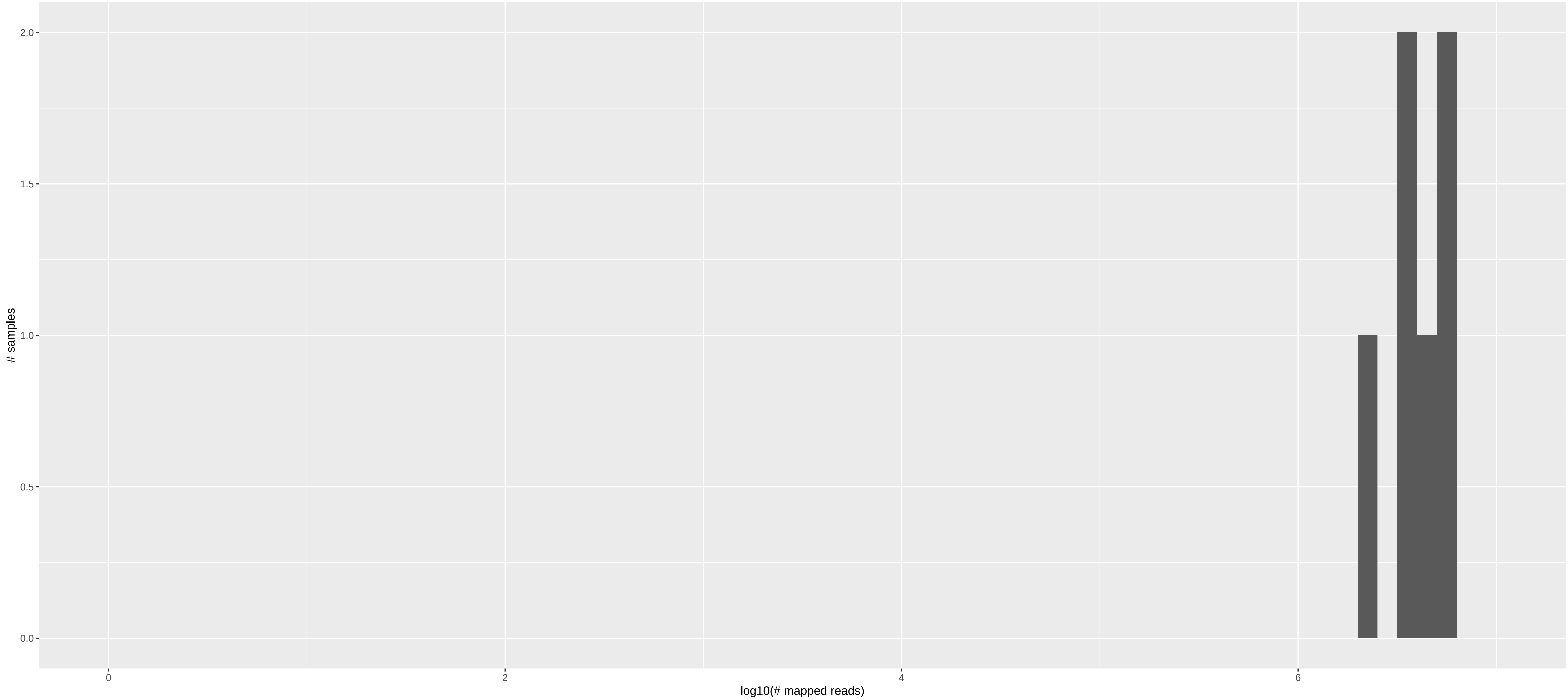

A histogram of library sizes. This plot provides an easy way to visualize the distribution of samples according to the number of their mapped reads. Ideally a consistent and robust library preparation and sequencing run should result in a tight distribution provided that samples have the same biological origin and have been handled with the same procedures prior to their library preparation (collection, storage and isolation).

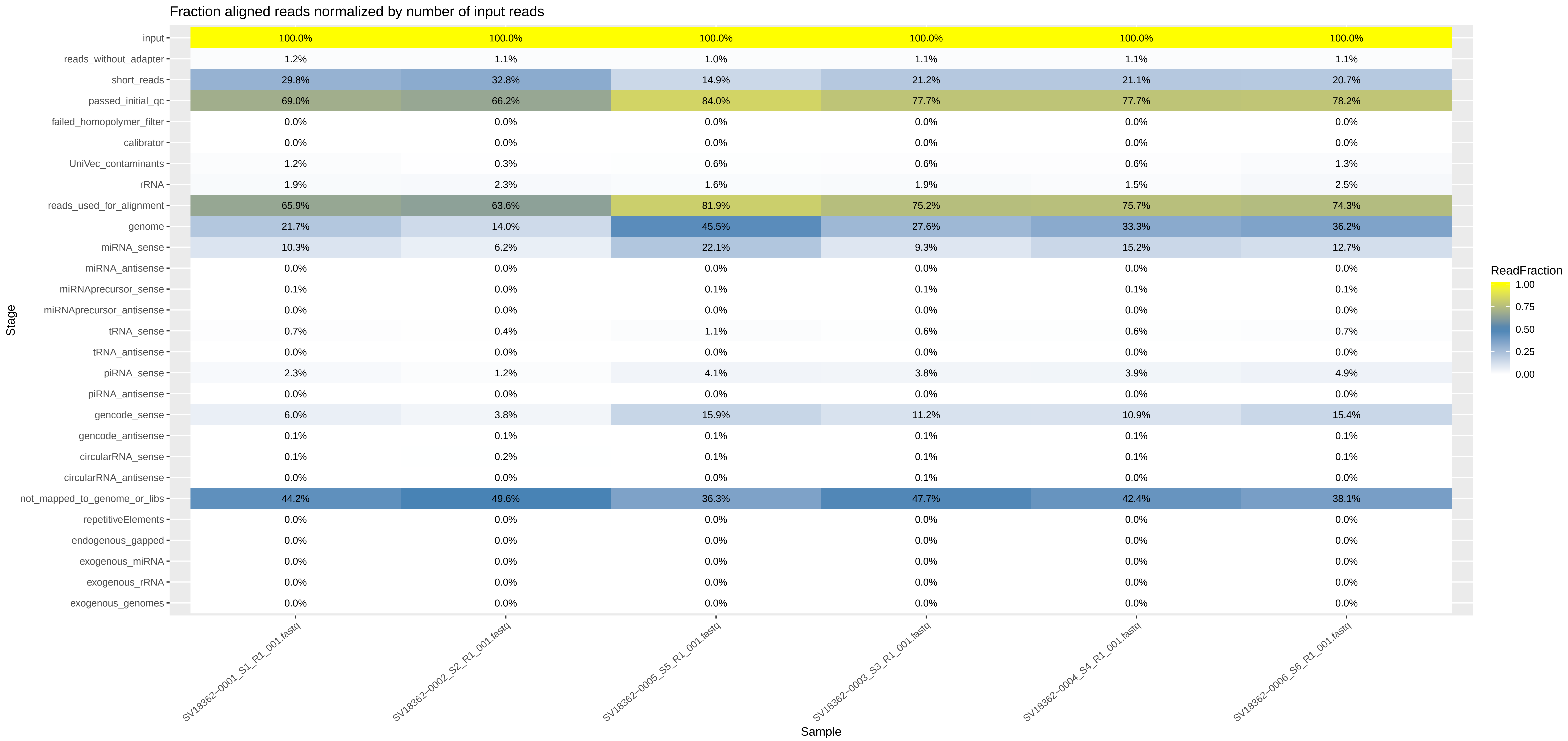

The percentage of reads is illustrated in a graphical table with a colored scheme that indicates fraction size and to allow for comparing fractions within a sample or across samples to oversee sample variations and their relative biotype distribution.

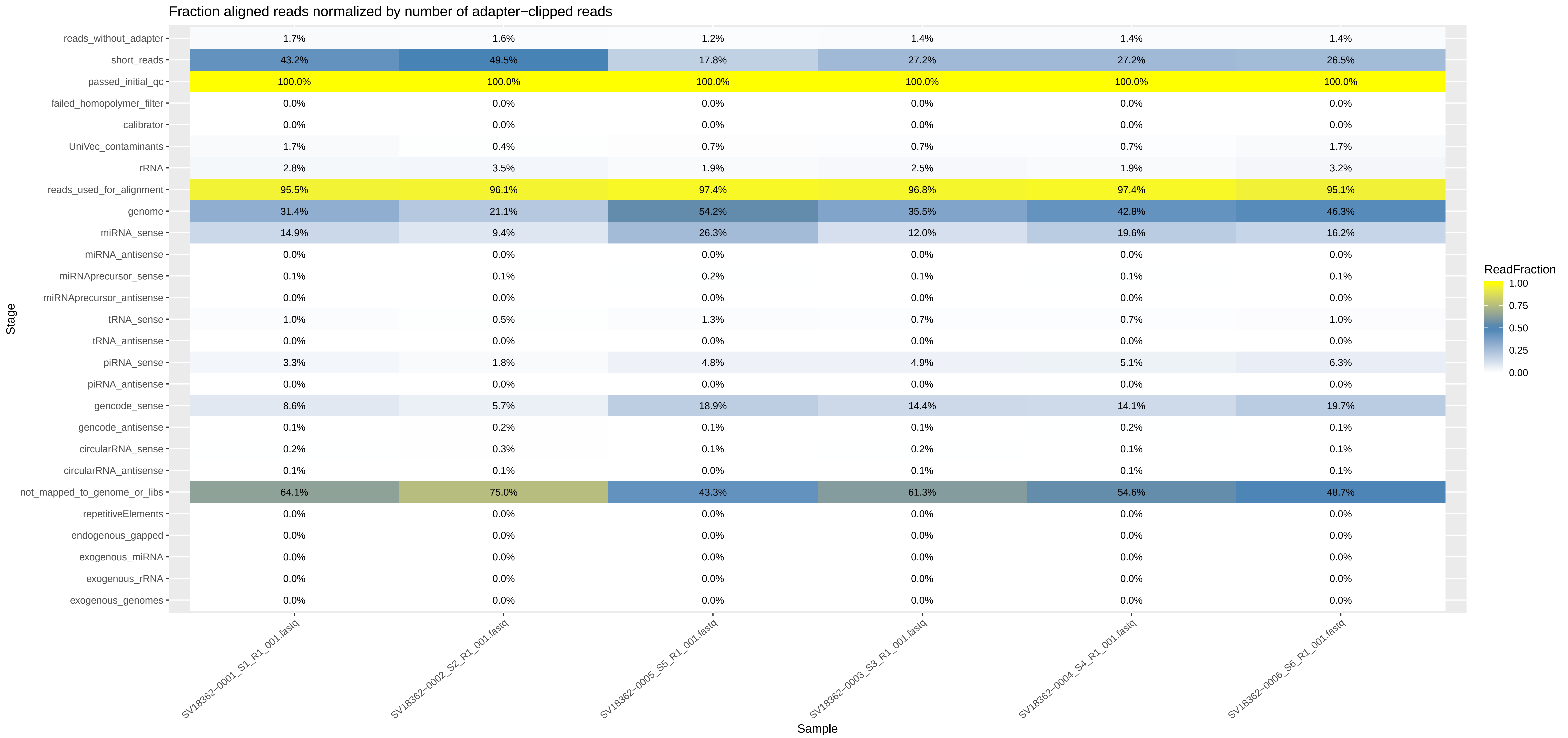

The percentage of reads is illustrated in a graphical table with a colored scheme that indicates fraction size and to allow for comparing fractions within a sample or across samples to oversee sample variations and their relative biotype distribution.

Fraction aligned reads normalized by number of non−contaminant reads

Stage	SV18362−0001_S1_R1_001.fastq	SV18362−0002_S2_R1_001.fastq	SV18362−0005_S5_R1_001.fastq	SV18362−0003_S3_R1_001.fastq	SV18362−0004_S4_R1_001.fastq	SV18362−0006_S6_R1_001.fastq
rRNA	2.9%	3.6%	2.0%	2.6%	1.9%	3.4%
reads_used_for_alignment	100.0%	100.0%	100.0%	100.0%	100.0%	100.0%
genome	32.9%	22.0%	55.6%	36.7%	43.9%	48.8%
miRNA_sense	15.6%	9.8%	27.0%	12.4%	20.1%	17.1%
miRNA_antisense	0.0%	0.0%	0.0%	0.0%	0.0%	0.0%
miRNAprecursor_sense	0.1%	0.1%	0.2%	0.1%	0.2%	0.1%
miRNAprecursor_antisense	0.0%	0.0%	0.0%	0.0%	0.0%	0.0%
tRNA_sense	1.1%	0.6%	1.3%	0.7%	0.7%	1.0%
tRNA_antisense	0.0%	0.0%	0.0%	0.0%	0.0%	0.0%
piRNA_sense	3.5%	1.8%	5.0%	5.1%	5.2%	6.6%
piRNA_antisense	0.0%	0.0%	0.0%	0.0%	0.0%	0.0%
gencode_sense	9.1%	6.0%	19.4%	14.8%	14.4%	20.8%
gencode_antisense	0.1%	0.2%	0.1%	0.1%	0.2%	0.1%
circularRNA_sense	0.2%	0.3%	0.1%	0.2%	0.1%	0.1%
circularRNA_antisense	0.1%	0.1%	0.0%	0.1%	0.1%	0.1%
not_mapped_to_genome_or_libs	67.1%	78.0%	44.4%	63.3%	56.1%	51.2%
repetitiveElements	0.0%	0.0%	0.0%	0.0%	0.0%	0.0%
endogenous_gapped	0.0%	0.0%	0.0%	0.0%	0.0%	0.0%
exogenous_miRNA	0.0%	0.0%	0.0%	0.0%	0.0%	0.0%
exogenous_rRNA	0.0%	0.0%	0.0%	0.0%	0.0%	0.0%
exogenous_genomes	0.0%	0.0%	0.0%	0.0%	0.0%	0.0%

Sample

ReadFraction: 1.00, 0.75, 0.50, 0.25, 0.00

The percentage of reads is illustrated in a graphical table with a colored scheme that indicates fraction size and to allow for comparing fractions within a sample or across samples to oversee sample variations and their relative biotype distribution.

Overall QC Results of Small RNA Analysis

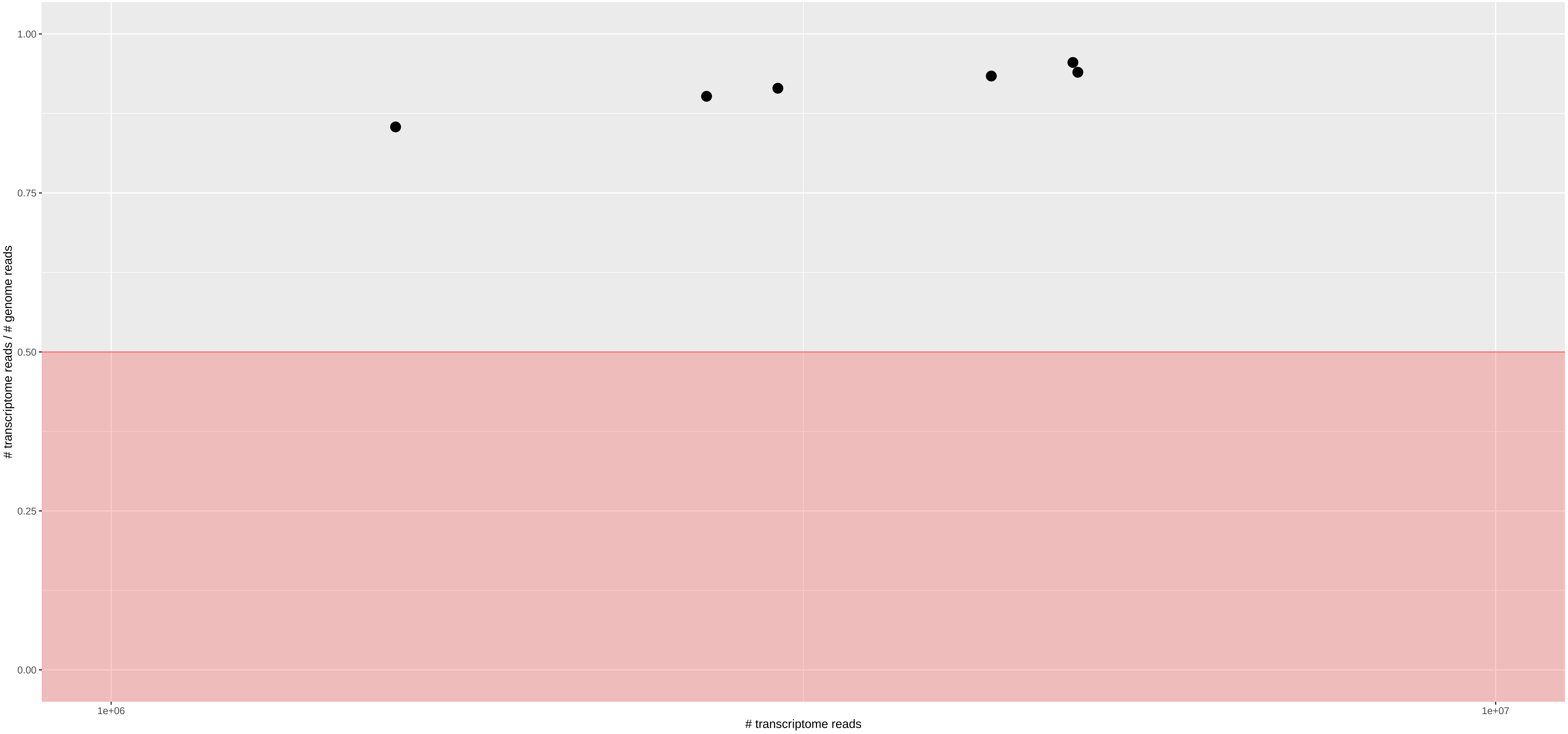

The graph indicates sample quality based on the small RNA analysis pipeline. Samples that pass QC have a number of transcriptome reads that exceeds 100,000 and transcriptome to genome ratio of > 0.5.

Small RNA analysis QC results per sample

	InputReads	GenomeReads	TranscriptomeReads	TranscriptomeGenomeRatio	TranscriptomeComplexity
SV18362−0006_S6_R1_001.fastq	14661166	5311251	4991408	0.94	0.003
SV18362−0004_S4_R1_001.fastq	13919922	4628382	4322262	0.93	0.004
SV18362−0003_S3_R1_001.fastq	12012745	3313663	3030857	0.91	0.005
SV18362−0005_S5_R1_001.fastq	11387899	5182676	4950530	0.96	0.003
SV18362−0002_S2_R1_001.fastq	13455935	1879663	1604893	0.85	0.008
SV18362−0001_S1_R1_001.fastq	13760245	2984488	2691911	0.9	0.005

The graph provides per sample analysis in an illustrated table where transcriptome reads and transcriptome genome ratio in addition to transcriptome complexity.
These values are highlighted to indicate if they do pass or fail QC.

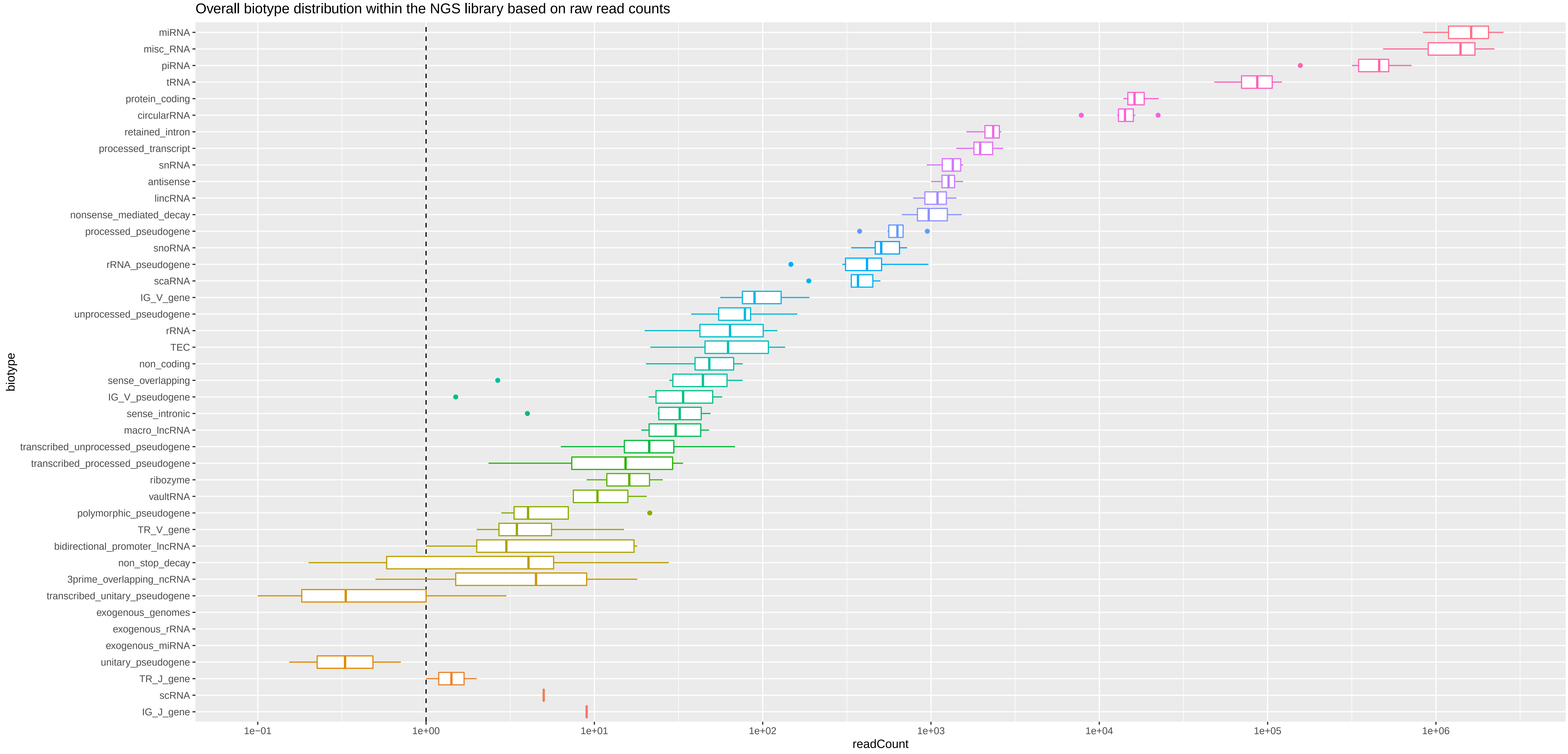

This analysis provides biotype fractions and their proportions across all samples in the NGS library.

Overall biotype distribution within the NGS library based on normalized counts

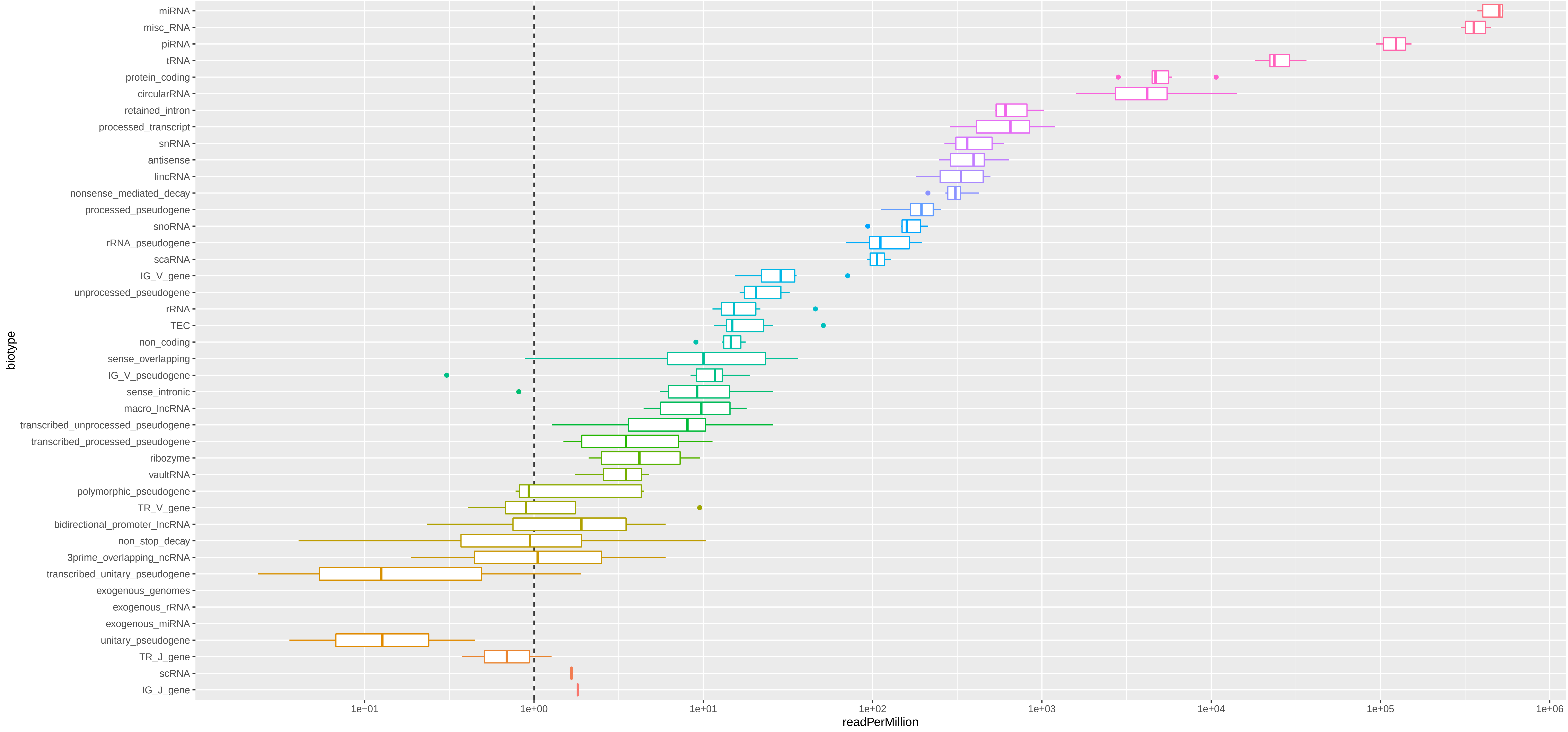

This analysis provides biotype fractions and their proportions across all samples in the NGS library.

Biotype distribution within the NGS library samples relative to reads used for alignment

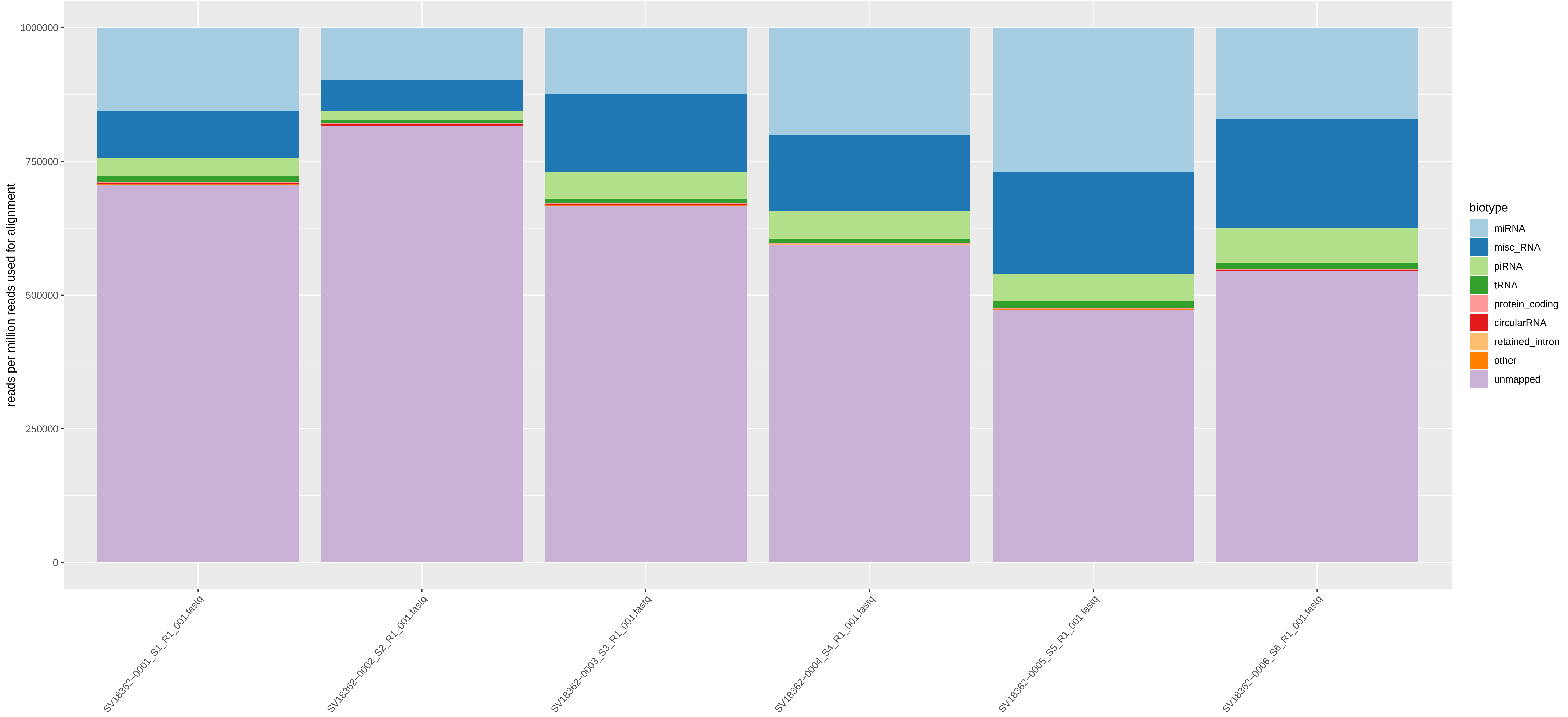

This graph provides biotype fractions within each sample as well as between samples.

Biotype distribution within the NGS library samples relative to mapped reads

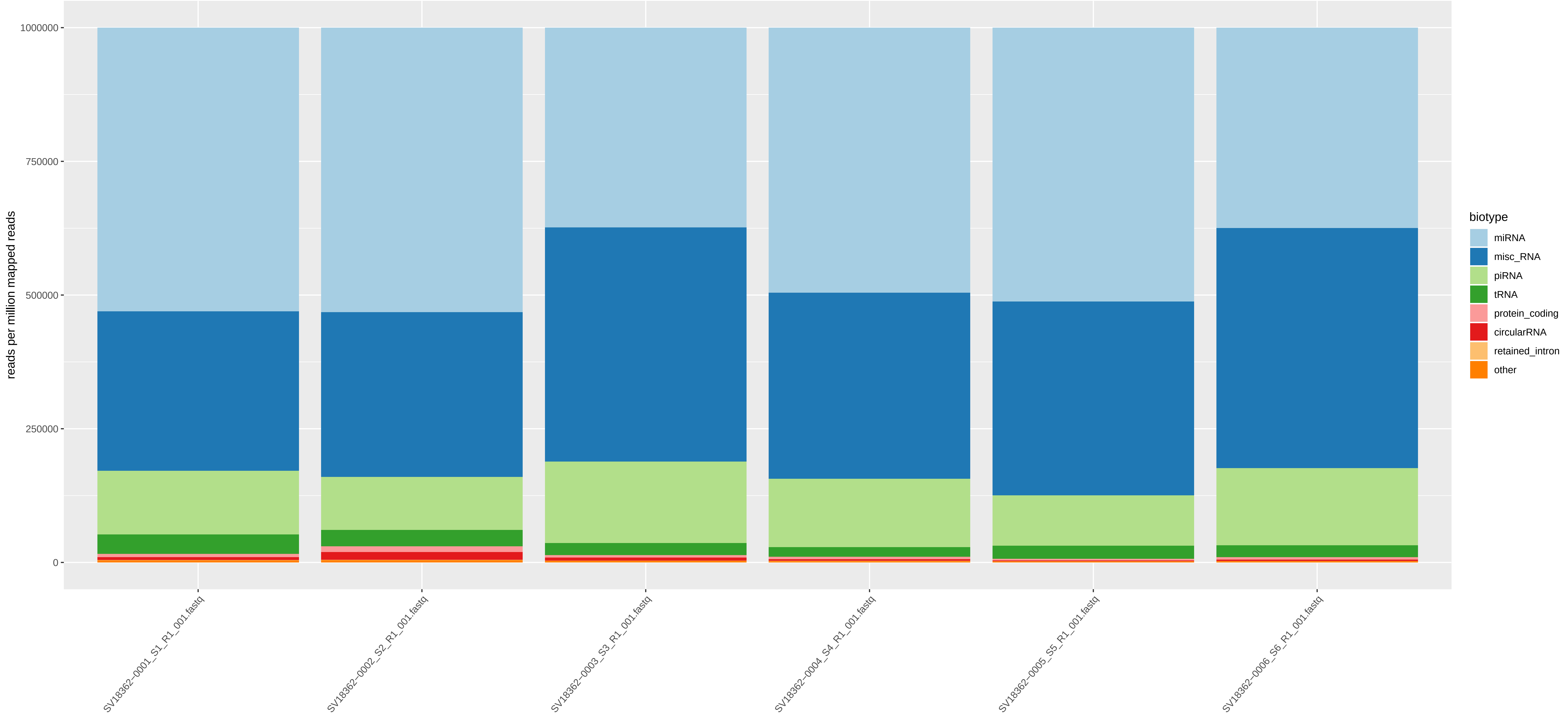

This graph provides biotype fractions within each sample as well as between samples.

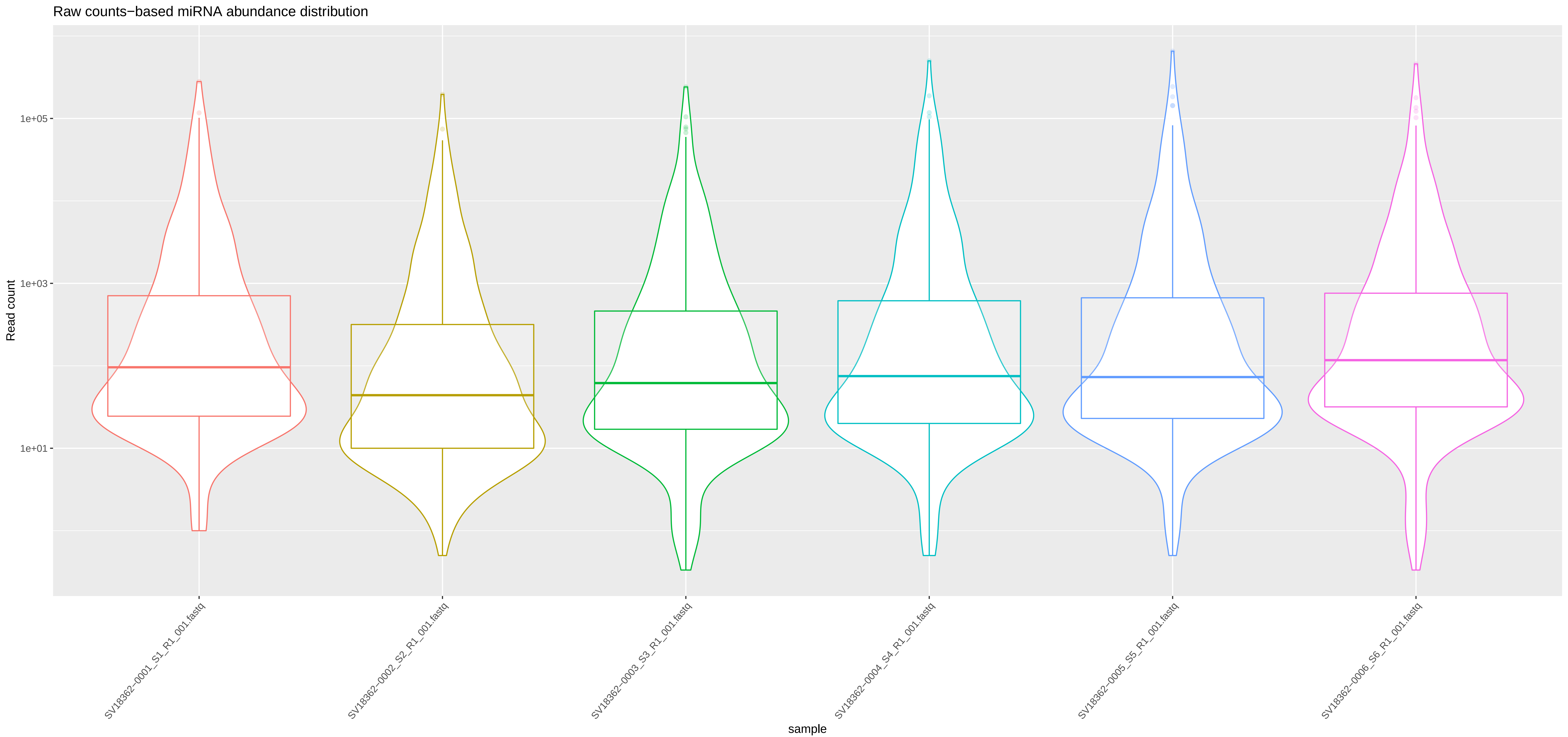

Abundance of miRNA categorizes the raw count of miRNAs within each sample, based on the number of miRNAs at each count. The normal distribution of the number of miRNAs in a sample is reversibly proportional to read counts.

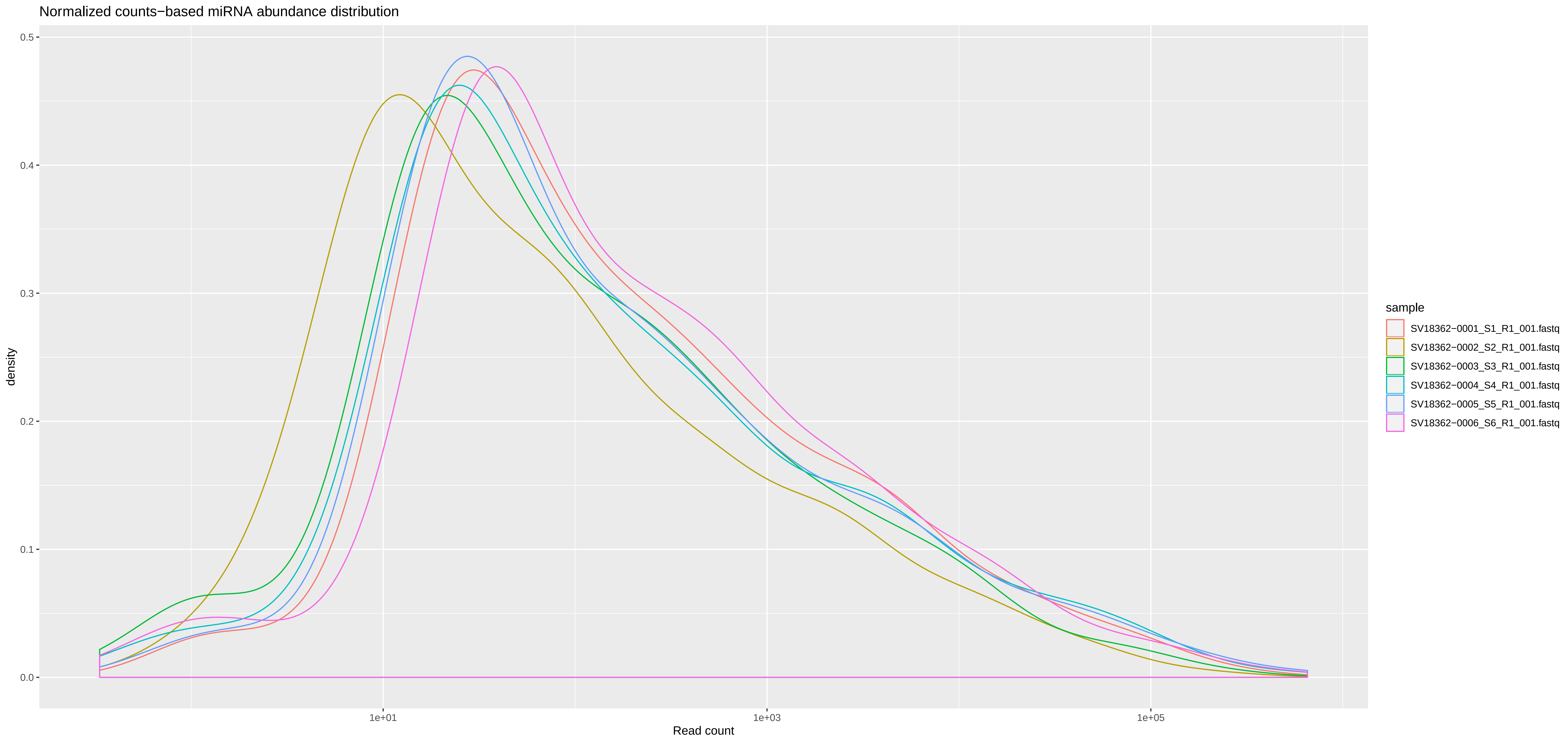

Abundance of miRNA categorizes the RPM normalized counts of miRNAs within each sample, based on the number of miRNAs at each count. The normal distribution of the number of miRNAs in a sample is reversibly proportional to read counts.

Density of miRNA abundance distribution based on raw counts

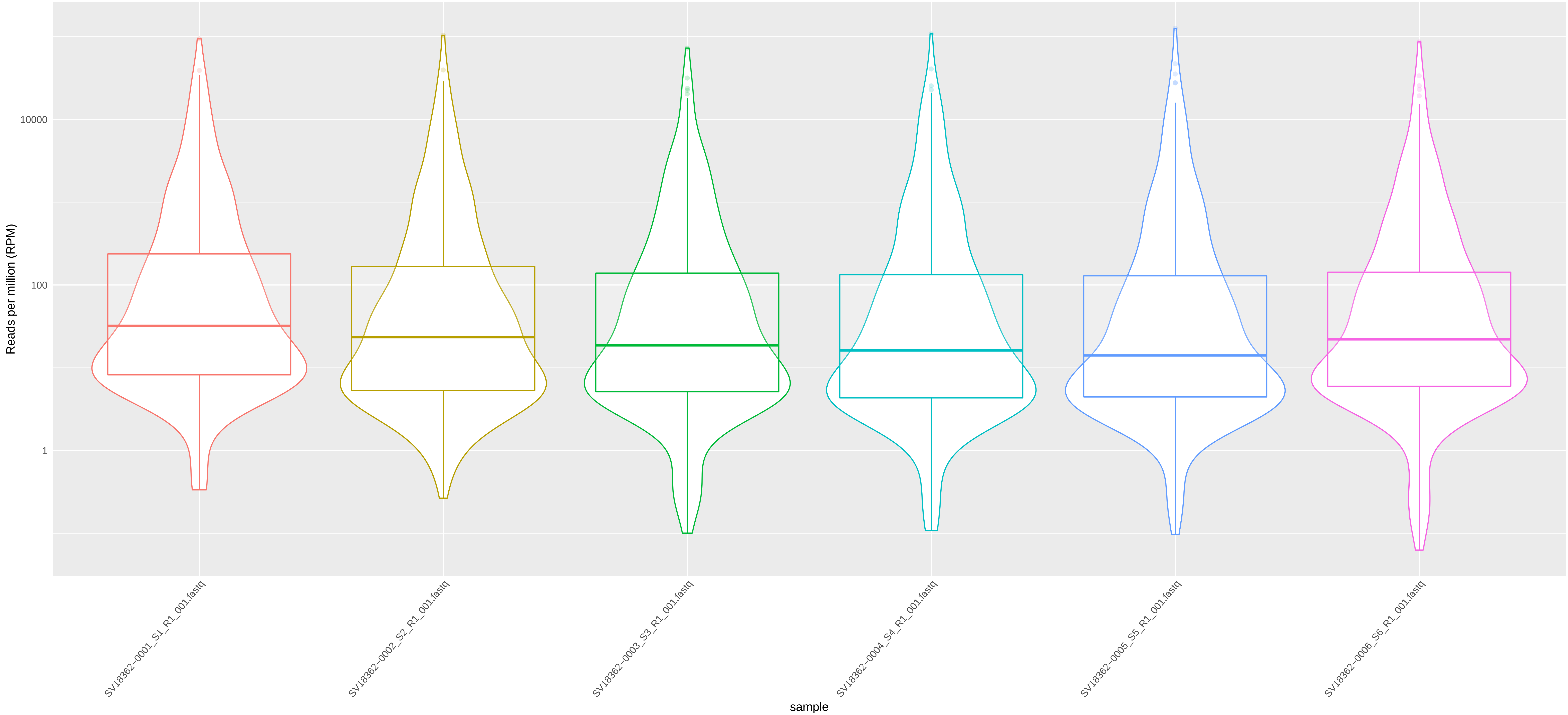

miRNA density in a sample is the number of miRNAs at a particular read count over the total number of miRNAs in a sample. Most miRNAs in a sample have low read counts and the density decreases at higher read counts.

Density of miRNA abundance distribution based on normalized counts

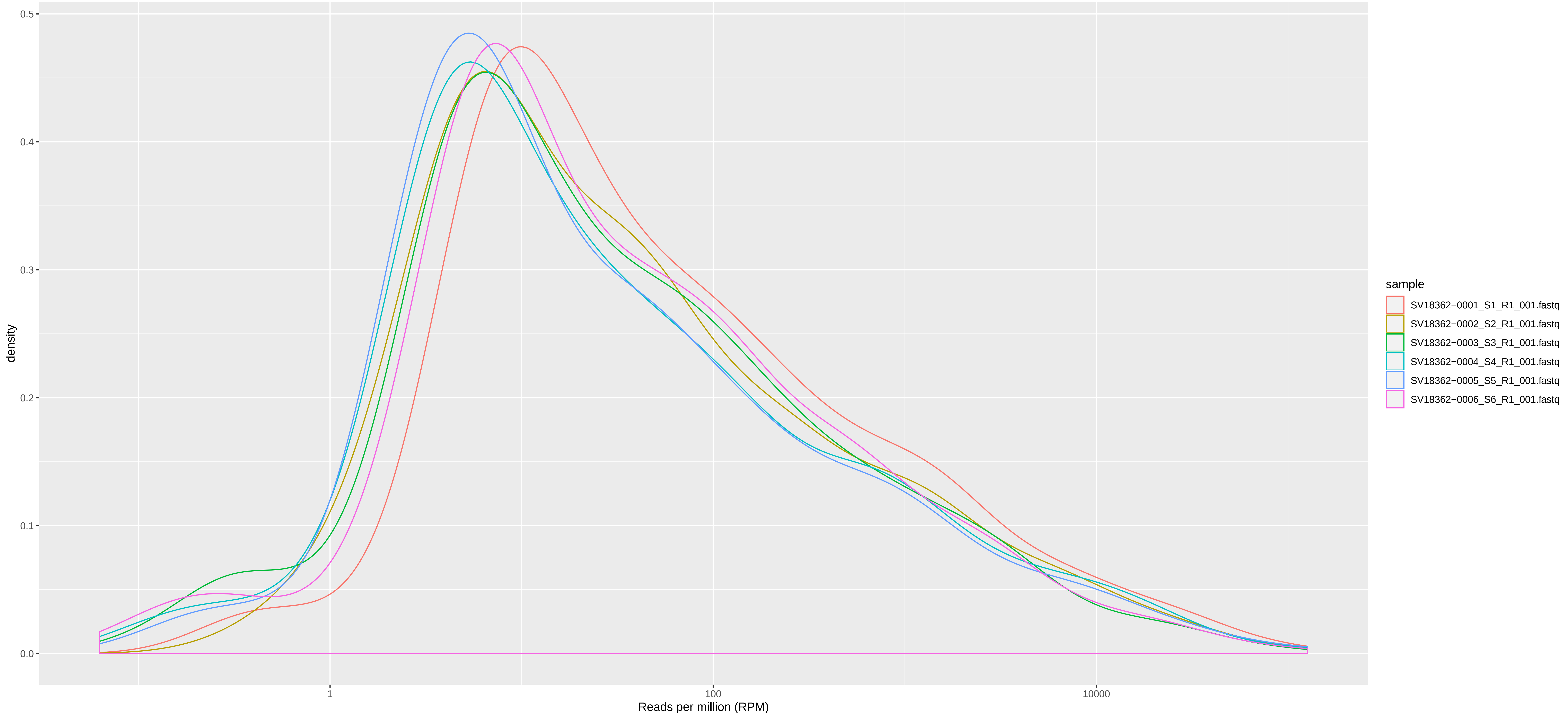

miRNA density in a sample is the number of miRNAs at a particular read count over the total number of miRNAs in a sample. Most miRNAs in a sample have low read counts and the density decreases at higher read counts.
