# Supplementary figures and images for: Analysis of plasma‐derived small extracellular vesicle characteristics and microRNA cargo following exercise‐induced skeletal muscle damage in men
Source: Physiol Rep. 2024 Sep 20;12(18):e70056. doi: 10.14814/phy2.70056 (PMC11415274; doi:10.14814/phy2.70056)

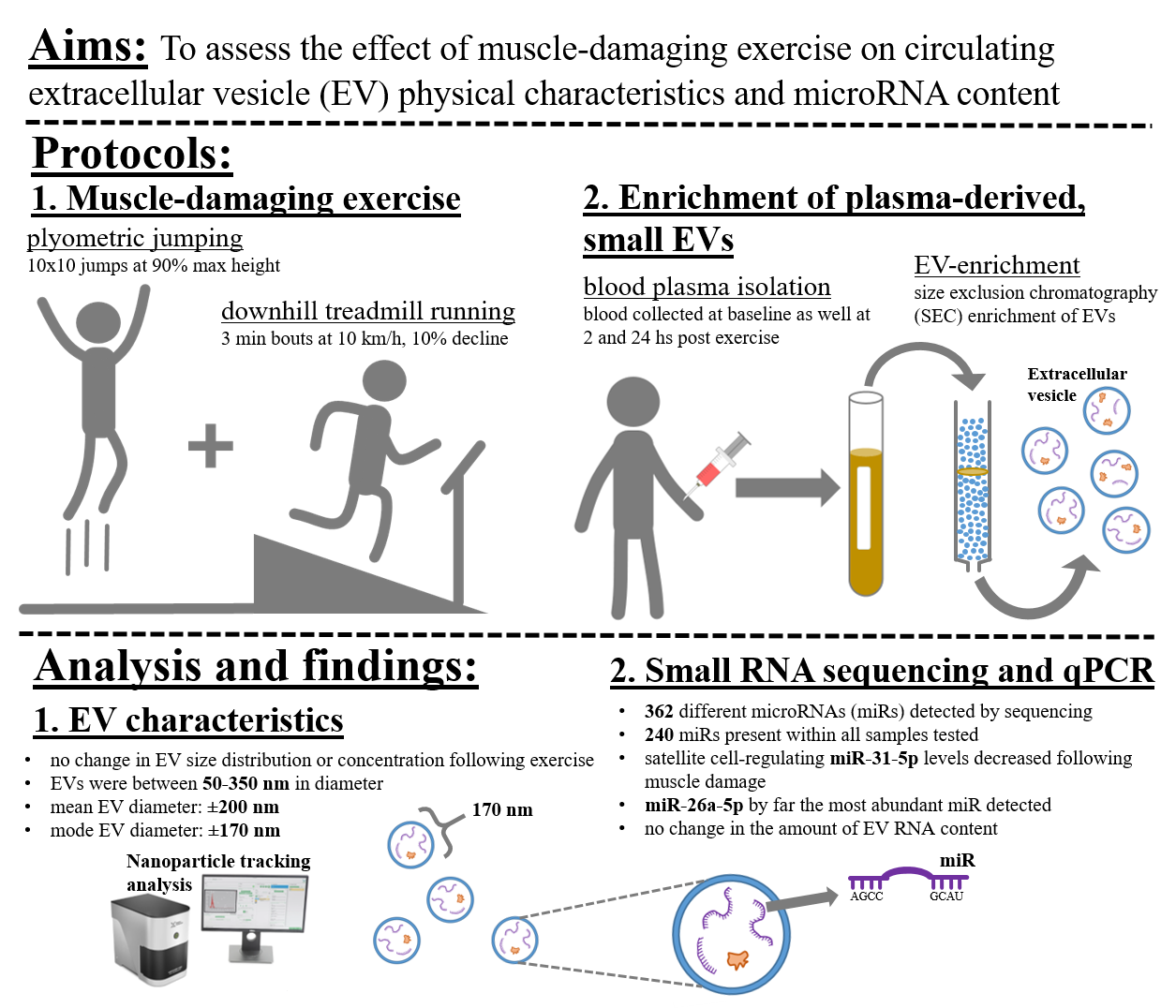

Supplement: Supplementary file 3 — Figure S1: Supporting Information. [file PHY2-12-e70056-s003.tif]
