# Supplementary material for: Analysis of plasma‐derived small extracellular vesicle characteristics and microRNA cargo following exercise‐induced skeletal muscle damage in men
Source: Physiol Rep. 2024 Sep 20;12(18):e70056. doi: 10.14814/phy2.70056 (PMC11415274; doi:10.14814/phy2.70056)
Supplement: Supplementary file 2 — Data S2: Supporting Information. [file PHY2-12-e70056-s001.zip › Processed Files For Individual Samples/SV18362-0001_S1_R1_001.fastq_CORE_RESULTS_v4.6.2.tgz!/SV18362-0001_S1_R1_001.fastq/SV18362-0001_S1_R1_001.fastq.clipped.trimmed.filtered_fastqc.zip!/SV18362-0001_S1_R1_001.fastq.clipped.trimmed.filtered_fastqc/fastqc_report.html]

SV18362-0001\_S1\_R1\_001.fastq.clipped.trimmed.filtered.fastq.gz FastQC Report 

FastQC Report

Fri 21 Feb 2020  
SV18362-0001\_S1\_R1\_001.fastq.clipped.trimmed.filtered.fastq.gz

## Summary

- Basic Statistics
- Per base sequence quality
- Per tile sequence quality
- Per sequence quality scores
- Per base sequence content
- Per sequence GC content
- Per base N content
- Sequence Length Distribution
- Sequence Duplication Levels
- Overrepresented sequences
- Adapter Content

## Basic Statistics

| Measure | Value |
| --- | --- |
| Filename | SV18362-0001\_S1\_R1\_001.fastq.clipped.trimmed.filtered.fastq.gz |
| File type | Conventional base calls |
| Encoding | Sanger / Illumina 1.9 |
| Total Sequences | 9495386 |
| Sequences flagged as poor quality | 0 |
| Sequence length | 18-47 |
| %GC | 53 |

## Per base sequence quality

## Per tile sequence quality

## Per sequence quality scores

## Per base sequence content

## Per sequence GC content

## Per base N content

## Sequence Length Distribution

## Sequence Duplication Levels

## Overrepresented sequences

| Sequence | Count | Percentage | Possible Source |
| --- | --- | --- | --- |
| GGCTGGTCCGATGGTAGTGGGTTATCAGAACT | 586648 | 6.178242780230314 | No Hit |
| CCCCCCACTGCTAAATTTGACTGGCTT | 295975 | 3.1170402130045054 | No Hit |
| TTCAAGTAATCCAGGATAGGCT | 260294 | 2.741268232802753 | No Hit |
| TGAGGTAGTAGATTGTATAGTT | 53951 | 0.5681812198050716 | No Hit |
| CCCCCACTGCTAAATTTGACTGGCTT | 53880 | 0.5674334882225957 | No Hit |
| TCGAGGAGCTCACAGTCTAGT | 53665 | 0.5651692306136896 | No Hit |
| TGAGGGGCAGAGAGCGAGACTTT | 52355 | 0.5513730563454714 | No Hit |
| CAACGGAATCCCAAAAGCAGCT | 46431 | 0.4889848606470553 | No Hit |
| TCCTGTACTGAGCTGCCCCGAG | 41666 | 0.4388025931752537 | No Hit |
| CAACGGAATCCCAAAAGCAGCTG | 39123 | 0.41202116480572776 | No Hit |
| AAATGATACGGCGACCACCGA | 36962 | 0.38926274297853714 | No Hit |
| TACCCTGTAGAACCGAATTTGT | 36238 | 0.3816379871234303 | No Hit |
| GGCTGGTCCGATGGTAGTGGGTTATCAGAAC | 30007 | 0.31601664218811115 | No Hit |
| CCCCCCACTGCTAAATTTGACTGGCTTT | 27726 | 0.2919944486722288 | No Hit |
| TGTAAACATCCCCGACTGGAAGCT | 26680 | 0.2809785721191324 | No Hit |
| TGAGAACTGAATTCCATGGGTT | 24292 | 0.25582951551416655 | No Hit |
| AGGCGCTTGAGAGAACTCGGG | 22114 | 0.2328920593644113 | No Hit |
| TCCCTGAGACCCTTTAACCTGT | 20795 | 0.21900110221954117 | No Hit |
| TGAGGTAGTAGGTTGTATAGTT | 20086 | 0.21153431782552073 | No Hit |
| CAGAGTTCTACAGTCCGACGATC | 19639 | 0.20682676828514399 | Illumina DpnII expression Sequencing Primer (100% over 23bp) |
| AATGATACGGCGACCACCGA | 18686 | 0.19679031479078365 | No Hit |
| TCCTGTACTGAGCTGCCCCGA | 18269 | 0.1923987081725798 | No Hit |
| TCCTGTACTGAGCTGCCCCGAGA | 17232 | 0.18147761449613528 | No Hit |
| AGAGTTCTACAGTCCGACGATC | 15939 | 0.16786047455048167 | Illumina DpnII expression Sequencing Primer (100% over 22bp) |
| TACCCTGTAGATCCGAATTTGT | 15819 | 0.16659670286178993 | No Hit |
| ACGTCGTGGAGGACCGAACC | 15654 | 0.16485901678983877 | No Hit |
| TGTAAACATCCTTGACTGGAAGCT | 15509 | 0.16333195933266959 | No Hit |
| GCATTGGTGGTTCAGTGGTAGAATTCTCGC | 14875 | 0.1566550322440815 | No Hit |
| ATCGGTGGTTCAGTGGTAGAATGC | 14828 | 0.15616005499934388 | No Hit |
| TCGAGGAGCTCACAGTCTAGTA | 14647 | 0.15425386603556715 | No Hit |
| GTAGGTAATCGTCAGGCT | 14566 | 0.15340082014570025 | No Hit |
| TGGCAACTTTGAGGTTCTAAC | 13586 | 0.1430800180213843 | No Hit |
| GCGCCCGTAGCTCAACGGATAGAGCACTTGAC | 13585 | 0.14306948659064517 | No Hit |
| TCCTGTACTGAGCTGCCCCGAGT | 13446 | 0.14160561771791058 | No Hit |
| TGAGAACTGAATTCCATGGGTTG | 12995 | 0.1368559424545774 | No Hit |
| TAGCTTATCAGACTGATGTTGACT | 12974 | 0.13663478240905635 | No Hit |
| AAAGTAGGTAATCGTCAGGCT | 12617 | 0.1328750616351984 | No Hit |
| ATTAAGCGAACCCGGGGAACTGA | 12476 | 0.13139012990098559 | No Hit |
| TGAGGTAGTAGATTGTATAGT | 12232 | 0.12882046080064571 | No Hit |
| GGCTGGTCCGATGGTAGTGGGTTATCAGAACTT | 12092 | 0.127346060497172 | No Hit |
| GAAAGTAGGTAATCGTCAGGCT | 11718 | 0.12340730540074939 | No Hit |
| GGCTGGTCCGATGGTAGTGGGTTATCAGAACA | 11599 | 0.12215406514279673 | No Hit |
| TGAGGGGCAGAGAGCGAGACTTTT | 11490 | 0.12100613919223506 | No Hit |
| GGCCACTTGGGACTCCTAAC | 11111 | 0.11701472694211695 | No Hit |
| AAGTAGGTAATCGTCAGGCT | 11096 | 0.11685675548103049 | No Hit |
| TGAAAGTAGGTAATCGTCAGGCT | 11086 | 0.11675144117363949 | No Hit |
| TTCTACAGTCCGACGATC | 11068 | 0.11656187542033573 | Illumina DpnII expression Sequencing Primer (100% over 18bp) |
| GTTCTACAGTCCGACGATC | 10758 | 0.11329713189121539 | Illumina DpnII expression Sequencing Primer (100% over 19bp) |
| AAAATGATACGGCGACCACCGA | 10618 | 0.11182273158774167 | No Hit |
| CTTGGTGAAGCGAACCCGGGGAACTGAAA | 10490 | 0.11047470845313714 | No Hit |
| AGTAGGTAATCGTCAGGCT | 10465 | 0.11021142268465968 | No Hit |
| TGAGAACTGAATTCCATAGGCTGT | 10057 | 0.10591459894310773 | No Hit |
| TGAGGTAGTAGGTTGTGTGGTT | 9968 | 0.10497730160732802 | No Hit |
| TTCACAGTGGCTAAGTTCTGC | 9770 | 0.10289207832098664 | No Hit |

## Adapter Content

Produced by FastQC (version 0.11.8)
