# Supplementary material for: Analysis of plasma‐derived small extracellular vesicle characteristics and microRNA cargo following exercise‐induced skeletal muscle damage in men
Source: Physiol Rep. 2024 Sep 20;12(18):e70056. doi: 10.14814/phy2.70056 (PMC11415274; doi:10.14814/phy2.70056)
Supplement: Supplementary file 2 — Data S2: Supporting Information. [file PHY2-12-e70056-s001.zip › Processed Files For Individual Samples/SV18362-0001_S1_R1_001.fastq_CORE_RESULTS_v4.6.2.tgz!/SV18362-0001_S1_R1_001.fastq/SV18362-0001_S1_R1_001.fastq.clipped.trimmed.filtered.noRiboRNA_fastqc.zip!/SV18362-0001_S1_R1_001.fastq.clipped.trimmed.filtered.noRiboRNA_fastqc/fastqc_report.html]

SV18362-0001\_S1\_R1\_001.fastq.clipped.trimmed.filtered.noRiboRNA.fastq.gz FastQC Report 

FastQC Report

Fri 21 Feb 2020  
SV18362-0001\_S1\_R1\_001.fastq.clipped.trimmed.filtered.noRiboRNA.fastq.gz

## Summary

- Basic Statistics
- Per base sequence quality
- Per tile sequence quality
- Per sequence quality scores
- Per base sequence content
- Per sequence GC content
- Per base N content
- Sequence Length Distribution
- Sequence Duplication Levels
- Overrepresented sequences
- Adapter Content

## Basic Statistics

| Measure | Value |
| --- | --- |
| Filename | SV18362-0001\_S1\_R1\_001.fastq.clipped.trimmed.filtered.noRiboRNA.fastq.gz |
| File type | Conventional base calls |
| Encoding | Sanger / Illumina 1.9 |
| Total Sequences | 9068732 |
| Sequences flagged as poor quality | 0 |
| Sequence length | 18-47 |
| %GC | 53 |

## Per base sequence quality

## Per tile sequence quality

## Per sequence quality scores

## Per base sequence content

## Per sequence GC content

## Per base N content

## Sequence Length Distribution

## Sequence Duplication Levels

## Overrepresented sequences

| Sequence | Count | Percentage | Possible Source |
| --- | --- | --- | --- |
| GGCTGGTCCGATGGTAGTGGGTTATCAGAACT | 586648 | 6.46890877357496 | No Hit |
| CCCCCCACTGCTAAATTTGACTGGCTT | 295975 | 3.263686698427079 | No Hit |
| TTCAAGTAATCCAGGATAGGCT | 260294 | 2.8702358830319388 | No Hit |
| TGAGGTAGTAGATTGTATAGTT | 53951 | 0.5949122766005215 | No Hit |
| CCCCCACTGCTAAATTTGACTGGCTT | 53880 | 0.5941293667074956 | No Hit |
| TCGAGGAGCTCACAGTCTAGT | 53665 | 0.5917585832286145 | No Hit |
| TGAGGGGCAGAGAGCGAGACTTT | 52355 | 0.5773133443572928 | No Hit |
| CAACGGAATCCCAAAAGCAGCT | 46431 | 0.5119899893391932 | No Hit |
| TCCTGTACTGAGCTGCCCCGAG | 41666 | 0.45944681130724785 | No Hit |
| CAACGGAATCCCAAAAGCAGCTG | 39123 | 0.43140540485704065 | No Hit |
| TACCCTGTAGAACCGAATTTGT | 36238 | 0.3995927986404273 | No Hit |
| GGCTGGTCCGATGGTAGTGGGTTATCAGAAC | 30007 | 0.33088418535248365 | No Hit |
| CCCCCCACTGCTAAATTTGACTGGCTTT | 27726 | 0.3057318266765409 | No Hit |
| TGTAAACATCCCCGACTGGAAGCT | 26680 | 0.294197689379287 | No Hit |
| TGAGAACTGAATTCCATGGGTT | 24292 | 0.26786545241385457 | No Hit |
| AGGCGCTTGAGAGAACTCGGG | 22114 | 0.2438488644277943 | No Hit |
| TCCCTGAGACCCTTTAACCTGT | 20795 | 0.22930438345735654 | No Hit |
| TGAGGTAGTAGGTTGTATAGTT | 20086 | 0.22148631142699993 | No Hit |
| TCCTGTACTGAGCTGCCCCGA | 18269 | 0.20145043430547954 | No Hit |
| TCCTGTACTGAGCTGCCCCGAGA | 17232 | 0.1900155391073416 | No Hit |
| TACCCTGTAGATCCGAATTTGT | 15819 | 0.17443452954613722 | No Hit |
| ACGTCGTGGAGGACCGAACC | 15654 | 0.17261509106234477 | No Hit |
| TGTAAACATCCTTGACTGGAAGCT | 15509 | 0.17101619057658776 | No Hit |
| GCATTGGTGGTTCAGTGGTAGAATTCTCGC | 14875 | 0.1640251360388641 | No Hit |
| ATCGGTGGTTCAGTGGTAGAATGC | 14828 | 0.1635068717434808 | No Hit |
| TCGAGGAGCTCACAGTCTAGTA | 14647 | 0.16151100286126 | No Hit |
| GTAGGTAATCGTCAGGCT | 14566 | 0.16061782396921642 | No Hit |
| TGGCAACTTTGAGGTTCTAAC | 13586 | 0.1498114620654795 | No Hit |
| GCGCCCGTAGCTCAACGGATAGAGCACTTGAC | 13585 | 0.14980043516557773 | No Hit |
| TCCTGTACTGAGCTGCCCCGAGT | 13446 | 0.14826769607923135 | No Hit |
| TGAGAACTGAATTCCATGGGTTG | 12995 | 0.143294564223532 | No Hit |
| TAGCTTATCAGACTGATGTTGACT | 12974 | 0.1430629993255948 | No Hit |
| AAAGTAGGTAATCGTCAGGCT | 12617 | 0.13912639606066207 | No Hit |
| ATTAAGCGAACCCGGGGAACTGA | 12476 | 0.13757160317451217 | No Hit |
| TGAGGTAGTAGATTGTATAGT | 12232 | 0.1348810395984797 | No Hit |
| GGCTGGTCCGATGGTAGTGGGTTATCAGAACTT | 12092 | 0.13333727361223158 | No Hit |
| GAAAGTAGGTAATCGTCAGGCT | 11718 | 0.1292132130489687 | No Hit |
| GGCTGGTCCGATGGTAGTGGGTTATCAGAACA | 11599 | 0.12790101196065778 | No Hit |
| TGAGGGGCAGAGAGCGAGACTTTT | 11490 | 0.12669907987136458 | No Hit |
| GGCCACTTGGGACTCCTAAC | 11111 | 0.12251988480859285 | No Hit |
| AAGTAGGTAATCGTCAGGCT | 11096 | 0.12235448131006628 | No Hit |
| TGAAAGTAGGTAATCGTCAGGCT | 11086 | 0.12224421231104855 | No Hit |
| CTTGGTGAAGCGAACCCGGGGAACTGAAA | 10490 | 0.11567217996959223 | No Hit |
| AGTAGGTAATCGTCAGGCT | 10465 | 0.11539650747204792 | No Hit |
| TGAGAACTGAATTCCATAGGCTGT | 10057 | 0.11089753231212478 | No Hit |
| TGAGGTAGTAGGTTGTGTGGTT | 9968 | 0.10991613822086704 | No Hit |
| TTCACAGTGGCTAAGTTCTGC | 9770 | 0.10773281204031612 | No Hit |
| GGCGCTTGAGAGAACTCGGG | 9383 | 0.1034654017783302 | No Hit |

## Adapter Content

Produced by FastQC (version 0.11.8)
