# Supplementary material for: Analysis of plasma‐derived small extracellular vesicle characteristics and microRNA cargo following exercise‐induced skeletal muscle damage in men
Source: Physiol Rep. 2024 Sep 20;12(18):e70056. doi: 10.14814/phy2.70056 (PMC11415274; doi:10.14814/phy2.70056)
Supplement: Supplementary file 2 — Data S2: Supporting Information. [file PHY2-12-e70056-s001.zip › Processed Files For Individual Samples/SV18362-0001_S1_R1_001.fastq_CORE_RESULTS_v4.6.2.tgz!/SV18362-0001_S1_R1_001.fastq/SV18362-0001_S1_R1_001.fastq.trim_adapter.fastq.tmp_fastqc.zip!/SV18362-0001_S1_R1_001.fastq.trim_adapter.fastq.tmp_fastqc/fastqc_report.html]

SV18362-0001\_S1\_R1\_001.fastq.trim\_adapter.fastq.tmp FastQC Report 

FastQC Report

Fri 21 Feb 2020  
SV18362-0001\_S1\_R1\_001.fastq.trim\_adapter.fastq.tmp

## Summary

- Basic Statistics
- Per base sequence quality
- Per tile sequence quality
- Per sequence quality scores
- Per base sequence content
- Per sequence GC content
- Per base N content
- Sequence Length Distribution
- Sequence Duplication Levels
- Overrepresented sequences
- Adapter Content

## Basic Statistics

| Measure | Value |
| --- | --- |
| Filename | SV18362-0001\_S1\_R1\_001.fastq.trim\_adapter.fastq.tmp |
| File type | Conventional base calls |
| Encoding | Sanger / Illumina 1.9 |
| Total Sequences | 13598824 |
| Sequences flagged as poor quality | 0 |
| Sequence length | 0-47 |
| %GC | 55 |

## Per base sequence quality

## Per tile sequence quality

## Per sequence quality scores

## Per base sequence content

## Per sequence GC content

## Per base N content

## Sequence Length Distribution

## Sequence Duplication Levels

## Overrepresented sequences

| Sequence | Count | Percentage | Possible Source |
| --- | --- | --- | --- |
| GGCTGGTCCGATGGTAGTGGGTTATCAGAACT | 586648 | 4.313961266062418 | No Hit |
| CCCCCCACTGCTAAATTTGACTGGCTT | 295975 | 2.1764749657764524 | No Hit |
| TTCAAGTAATCCAGGATAGGCT | 260294 | 1.9140919832479633 | No Hit |
|  | 161572 | 1.1881321502506392 | No Hit |
| TGAGGTAGTAGATTGTATAGTT | 53951 | 0.39673283513339097 | No Hit |
| CCCCCACTGCTAAATTTGACTGGCTT | 53880 | 0.39621073116322414 | No Hit |
| TCGAGGAGCTCACAGTCTAGT | 53665 | 0.3946297120986344 | No Hit |
| TGAGGGGCAGAGAGCGAGACTTT | 52355 | 0.38499652617020413 | No Hit |
| CAACGGAATCCCAAAAGCAGCT | 46431 | 0.34143393575797437 | No Hit |
| TCCTGTACTGAGCTGCCCCGAG | 41666 | 0.30639414114043984 | No Hit |
| CAACGGAATCCCAAAAGCAGCTG | 39123 | 0.28769399471601365 | No Hit |
| AAATGATACGGCGACCACCGA | 36962 | 0.271802914722626 | No Hit |
| TACCCTGTAGAACCGAATTTGT | 36238 | 0.2664789249423333 | No Hit |
| GGCTGGTCCGATGGTAGTGGGTTATCAGAAC | 30007 | 0.2206587863774103 | No Hit |
| AGGCCGAGGACTTACC | 28407 | 0.20889306310604505 | No Hit |
| CCCCCCACTGCTAAATTTGACTGGCTTT | 27726 | 0.20388527713867022 | No Hit |
| TGTAAACATCCCCGACTGGAAGCT | 26680 | 0.19619343555001523 | No Hit |
| TGAGAACTGAATTCCATGGGTT | 24292 | 0.1786330935675026 | No Hit |
| AGGCGCTTGAGAGAACTCGGG | 22114 | 0.16261700276435667 | No Hit |
| CGCGGGGTGGAGC | 21692 | 0.1595137932515341 | No Hit |
| TCTACAGTCCGACGATC | 21186 | 0.15579288326696486 | Illumina DpnII expression Sequencing Primer (100% over 17bp) |
| TCCCTGAGACCCTTTAACCTGT | 20795 | 0.15291763464252497 | No Hit |
| TGAGGTAGTAGGTTGTATAGTT | 20086 | 0.14770394851790125 | No Hit |
| CAGAGTTCTACAGTCCGACGATC | 19639 | 0.1444168995789636 | Illumina DpnII expression Sequencing Primer (100% over 23bp) |
| AATGATACGGCGACCACCGA | 18686 | 0.13740894065545667 | No Hit |
| TCCTGTACTGAGCTGCCCCGA | 18269 | 0.1343424990278571 | No Hit |
| TCCTGTACTGAGCTGCCCCGAGA | 17232 | 0.12671683963260352 | No Hit |
| GCGCCCGTAGCTCAAC | 16807 | 0.12359156938864713 | No Hit |
| AGAGTTCTACAGTCCGACGATC | 15939 | 0.1172086645139315 | Illumina DpnII expression Sequencing Primer (100% over 22bp) |
| TACCCTGTAGATCCGAATTTGT | 15819 | 0.11632623526857912 | No Hit |
| ACGTCGTGGAGGACCGAACC | 15654 | 0.11511289505621958 | No Hit |
| TGTAAACATCCTTGACTGGAAGCT | 15509 | 0.1140466263847521 | No Hit |
| GCATTGGTGGTTCAGTGGTAGAATTCTCGC | 14875 | 0.10938445853847362 | No Hit |
| ATCGGTGGTTCAGTGGTAGAATGC | 14828 | 0.10903884041737727 | No Hit |
| TCGAGGAGCTCACAGTCTAGTA | 14647 | 0.10770784297230408 | No Hit |
| GTAGGTAATCGTCAGGCT | 14566 | 0.10711220323169121 | No Hit |

## Adapter Content

Produced by FastQC (version 0.11.8)
