# Supplementary material for: Analysis of plasma‐derived small extracellular vesicle characteristics and microRNA cargo following exercise‐induced skeletal muscle damage in men
Source: Physiol Rep. 2024 Sep 20;12(18):e70056. doi: 10.14814/phy2.70056 (PMC11415274; doi:10.14814/phy2.70056)
Supplement: Supplementary file 2 — Data S2: Supporting Information. [file PHY2-12-e70056-s001.zip › Processed Files For Individual Samples/SV18362-0005_S5_R1_001.fastq_CORE_RESULTS_v4.6.2.tgz!/SV18362-0005_S5_R1_001.fastq/SV18362-0005_S5_R1_001.fastq.clipped.trimmed.filtered_fastqc.zip!/SV18362-0005_S5_R1_001.fastq.clipped.trimmed.filtered_fastqc/fastqc_report.html]

SV18362-0005\_S5\_R1\_001.fastq.clipped.trimmed.filtered.fastq.gz FastQC Report 

FastQC Report

Fri 21 Feb 2020  
SV18362-0005\_S5\_R1\_001.fastq.clipped.trimmed.filtered.fastq.gz

## Summary

- Basic Statistics
- Per base sequence quality
- Per tile sequence quality
- Per sequence quality scores
- Per base sequence content
- Per sequence GC content
- Per base N content
- Sequence Length Distribution
- Sequence Duplication Levels
- Overrepresented sequences
- Adapter Content

## Basic Statistics

| Measure | Value |
| --- | --- |
| Filename | SV18362-0005\_S5\_R1\_001.fastq.clipped.trimmed.filtered.fastq.gz |
| File type | Conventional base calls |
| Encoding | Sanger / Illumina 1.9 |
| Total Sequences | 9568910 |
| Sequences flagged as poor quality | 0 |
| Sequence length | 18-47 |
| %GC | 51 |

## Per base sequence quality

## Per tile sequence quality

## Per sequence quality scores

## Per base sequence content

## Per sequence GC content

## Per base N content

## Sequence Length Distribution

## Sequence Duplication Levels

## Overrepresented sequences

| Sequence | Count | Percentage | Possible Source |
| --- | --- | --- | --- |
| GGCTGGTCCGATGGTAGTGGGTTATCAGAACT | 1433441 | 14.980191056243605 | No Hit |
| TTCAAGTAATCCAGGATAGGCT | 610610 | 6.3811865719293 | No Hit |
| CCCCCCACTGCTAAATTTGACTGGCTT | 432545 | 4.5203163160694375 | No Hit |
| TGAGGTAGTAGATTGTATAGTT | 128463 | 1.3425040051583723 | No Hit |
| TCGAGGAGCTCACAGTCTAGT | 109966 | 1.1492009016700961 | No Hit |
| CAACGGAATCCCAAAAGCAGCT | 93013 | 0.9720333872928055 | No Hit |
| TGAGGGGCAGAGAGCGAGACTTT | 91066 | 0.9516862422156755 | No Hit |
| CAACGGAATCCCAAAAGCAGCTG | 85392 | 0.8923900423350204 | No Hit |
| CCCCCACTGCTAAATTTGACTGGCTT | 75028 | 0.7840809454786386 | No Hit |
| TGTAAACATCCCCGACTGGAAGCT | 63454 | 0.663126730212741 | No Hit |
| GGCTGGTCCGATGGTAGTGGGTTATCAGAAC | 49057 | 0.5126707221616673 | No Hit |
| TGAGGTAGTAGGTTGTATAGTT | 48084 | 0.5025023748786435 | No Hit |
| CCCCCCACTGCTAAATTTGACTGGCTTT | 44751 | 0.4676708214415226 | No Hit |
| TAGCTTATCAGACTGATGTTGACT | 40045 | 0.4184907162884801 | No Hit |
| GCATTGGTGGTTCAGTGGTAGAATTCTCGCCT | 38668 | 0.40410036252822945 | No Hit |
| TGTAAACATCCTTGACTGGAAGCT | 35590 | 0.37193368941708094 | No Hit |
| GGCTGGTCCGATGGTAGTGGGTTATCAGAACTT | 34361 | 0.3590900112970025 | No Hit |
| TGAGAACTGAATTCCATGGGTT | 31902 | 0.3333922045457633 | No Hit |
| TCGAGGAGCTCACAGTCTAGTA | 30084 | 0.31439317539824285 | No Hit |
| AAATGATACGGCGACCACCGA | 29251 | 0.3056878996667332 | No Hit |
| TCCTGTACTGAGCTGCCCCGAG | 24977 | 0.26102241530122033 | No Hit |
| TACCCTGTAGAACCGAATTTGT | 24760 | 0.25875465439637324 | No Hit |
| TGAGGGGCAGAGAGCGAGACTTTT | 24381 | 0.2547939106962026 | No Hit |
| TGAGGTAGTAGATTGTATAGTTT | 24122 | 0.2520872283259013 | No Hit |
| TGAGAACTGAATTCCATAGGCTGT | 23910 | 0.24987171997646546 | No Hit |
| TTCACAGTGGCTAAGTTCTGC | 22094 | 0.23089359185110947 | No Hit |
| GTAGGTAATCGTCAGGCT | 22032 | 0.23024566016401032 | No Hit |
| TCCCTGAGACCCTTTAACCTGT | 21736 | 0.22715230888366594 | No Hit |
| TGAGGTAGTAGATTGTATAGT | 20922 | 0.21864559286271895 | No Hit |
| GGCTGGTCCGATGGTAGTGGGTTATCAGAACA | 20525 | 0.2144967399630679 | No Hit |
| TAGCTTATCAGACTGATGTTGAC | 20412 | 0.21331583221077427 | No Hit |
| TCAGTGCATCACAGAACTTTGTTT | 19898 | 0.20794426951450062 | No Hit |
| TGAAAGTAGGTAATCGTCAGGCT | 18529 | 0.19363751984290792 | No Hit |
| AAAGTAGGTAATCGTCAGGCT | 18106 | 0.1892169536551185 | No Hit |
| GCTGGTCCGATGGTAGTGGGTTATCAGAACT | 17094 | 0.1786410364398871 | No Hit |
| CAACGGAATCCCAAAAGCAGCTGT | 16924 | 0.17686444955590552 | No Hit |
| TCCTGTACTGAGCTGCCCCGAGT | 16646 | 0.17395920747504157 | No Hit |
| AGTAGGTAATCGTCAGGCT | 16447 | 0.17187955576967492 | No Hit |
| AAGTAGGTAATCGTCAGGCT | 16179 | 0.16907881879963338 | No Hit |
| TACCCTGTAGATCCGAATTTGT | 16154 | 0.16881755602257728 | No Hit |
| GAAAGTAGGTAATCGTCAGGCT | 16127 | 0.16853539222335667 | No Hit |
| TGAGGTAGTAGGTTGTGTGGTT | 15815 | 0.1652748327656964 | No Hit |
| CAACGGAATCCCAAAAGCAGCTT | 15106 | 0.15786542040838508 | No Hit |
| CACCCGTAGAACCGACCTTGCG | 14262 | 0.14904518905497074 | No Hit |
| AATGATACGGCGACCACCGA | 14106 | 0.1474149093261406 | No Hit |
| AGCTCGGTCTGAGGCCCCTCAGT | 13782 | 0.14402894373549338 | No Hit |
| GTGAAAGTAGGTAATCGTCAGGCT | 13622 | 0.14235686196233427 | No Hit |
| TGAGAACTGAATTCCATGGGTTG | 13547 | 0.14157307363116592 | No Hit |
| TATTGCACTTGTCCCGGCCTGTAT | 13153 | 0.13745557226476163 | No Hit |
| TCCTGTACTGAGCTGCCCCGAGA | 12498 | 0.1306104875058915 | No Hit |
| GTGTGAAAGTAGGTAATCGTCAGGCT | 12260 | 0.1281232658683173 | No Hit |
| TGTAAACATCCTACACTCTCAGCT | 11779 | 0.1230965700377577 | No Hit |
| TCCTGTACTGAGCTGCCCCGA | 11628 | 0.12151854286433879 | No Hit |
| CCCCCCACTGCTAAATTTGACTGGCTA | 11618 | 0.12141403775351634 | No Hit |
| CCGTGTGAAAGTAGGTAATCGTCAGGCT | 11500 | 0.12018087744581148 | No Hit |
| TGAGGTAGTAGTTTGTGCTGTT | 11366 | 0.11878050896079073 | No Hit |
| TGAGAACTGAATTCCATGGGTTGT | 11160 | 0.11662770367784837 | No Hit |
| TATTGCACTTGTCCCGGCCTGT | 11007 | 0.11502877548226495 | No Hit |
| TTCAAGTAATCCAGGATAGGC | 10991 | 0.11486156730494905 | No Hit |
| AGTTGGTCCGAGTGTTGTGGGTTATTGTTAA | 10845 | 0.11333579268694134 | No Hit |
| TGTGAAAGTAGGTAATCGTCAGGCT | 10673 | 0.11153830478079531 | No Hit |
| CGTGTGAAAGTAGGTAATCGTCAGGCT | 10237 | 0.10698188194893671 | No Hit |
| CGGAATGTAGCGCAGTTGGTAGC | 9784 | 0.10224780042867997 | No Hit |

## Adapter Content

Produced by FastQC (version 0.11.8)
