# Supplementary material for: Analysis of plasma‐derived small extracellular vesicle characteristics and microRNA cargo following exercise‐induced skeletal muscle damage in men
Source: Physiol Rep. 2024 Sep 20;12(18):e70056. doi: 10.14814/phy2.70056 (PMC11415274; doi:10.14814/phy2.70056)
Supplement: Supplementary file 2 — Data S2: Supporting Information. [file PHY2-12-e70056-s001.zip › Processed Files For Individual Samples/SV18362-0005_S5_R1_001.fastq_CORE_RESULTS_v4.6.2.tgz!/SV18362-0005_S5_R1_001.fastq/SV18362-0005_S5_R1_001.fastq.clipped.trimmed.filtered.noRiboRNA_fastqc.zip!/SV18362-0005_S5_R1_001.fastq.clipped.trimmed.filtered.noRiboRNA_fastqc/fastqc_report.html]

SV18362-0005\_S5\_R1\_001.fastq.clipped.trimmed.filtered.noRiboRNA.fastq.gz FastQC Report 

FastQC Report

Fri 21 Feb 2020  
SV18362-0005\_S5\_R1\_001.fastq.clipped.trimmed.filtered.noRiboRNA.fastq.gz

## Summary

- Basic Statistics
- Per base sequence quality
- Per tile sequence quality
- Per sequence quality scores
- Per base sequence content
- Per sequence GC content
- Per base N content
- Sequence Length Distribution
- Sequence Duplication Levels
- Overrepresented sequences
- Adapter Content

## Basic Statistics

| Measure | Value |
| --- | --- |
| Filename | SV18362-0005\_S5\_R1\_001.fastq.clipped.trimmed.filtered.noRiboRNA.fastq.gz |
| File type | Conventional base calls |
| Encoding | Sanger / Illumina 1.9 |
| Total Sequences | 9322044 |
| Sequences flagged as poor quality | 0 |
| Sequence length | 18-47 |
| %GC | 51 |

## Per base sequence quality

## Per tile sequence quality

## Per sequence quality scores

## Per base sequence content

## Per sequence GC content

## Per base N content

## Sequence Length Distribution

## Sequence Duplication Levels

## Overrepresented sequences

| Sequence | Count | Percentage | Possible Source |
| --- | --- | --- | --- |
| GGCTGGTCCGATGGTAGTGGGTTATCAGAACT | 1433441 | 15.376895882491008 | No Hit |
| TTCAAGTAATCCAGGATAGGCT | 610610 | 6.550172901994456 | No Hit |
| CCCCCCACTGCTAAATTTGACTGGCTT | 432545 | 4.640023153720365 | No Hit |
| TGAGGTAGTAGATTGTATAGTT | 128463 | 1.3780561430518887 | No Hit |
| TCGAGGAGCTCACAGTCTAGT | 109966 | 1.1796339944329806 | No Hit |
| CAACGGAATCCCAAAAGCAGCT | 93013 | 0.9977747369568305 | No Hit |
| TGAGGGGCAGAGAGCGAGACTTT | 91066 | 0.976888759589635 | No Hit |
| CAACGGAATCCCAAAAGCAGCTG | 85392 | 0.9160222800922201 | No Hit |
| CCCCCACTGCTAAATTTGACTGGCTT | 75028 | 0.8048449460225676 | No Hit |
| TGTAAACATCCCCGACTGGAAGCT | 63454 | 0.6806876260184999 | No Hit |
| GGCTGGTCCGATGGTAGTGGGTTATCAGAAC | 49057 | 0.5262472479211641 | No Hit |
| TGAGGTAGTAGGTTGTATAGTT | 48084 | 0.5158096228681177 | No Hit |
| CCCCCCACTGCTAAATTTGACTGGCTTT | 44751 | 0.4800556616124103 | No Hit |
| TAGCTTATCAGACTGATGTTGACT | 40045 | 0.42957317086252755 | No Hit |
| GCATTGGTGGTTCAGTGGTAGAATTCTCGCCT | 38668 | 0.41480173232394096 | No Hit |
| TGTAAACATCCTTGACTGGAAGCT | 35590 | 0.3817832226494533 | No Hit |
| GGCTGGTCCGATGGTAGTGGGTTATCAGAACTT | 34361 | 0.3685994187540844 | No Hit |
| TGAGAACTGAATTCCATGGGTT | 31902 | 0.34222108370224386 | No Hit |
| TCGAGGAGCTCACAGTCTAGTA | 30084 | 0.3227189230173125 | No Hit |
| TCCTGTACTGAGCTGCCCCGAG | 24977 | 0.2679348005651979 | No Hit |
| TACCCTGTAGAACCGAATTTGT | 24760 | 0.26560698490588547 | No Hit |
| TGAGGGGCAGAGAGCGAGACTTTT | 24381 | 0.2615413529479157 | No Hit |
| TGAGGTAGTAGATTGTATAGTTT | 24122 | 0.25876299232228467 | No Hit |
| TGAGAACTGAATTCCATAGGCTGT | 23910 | 0.2564888129684863 | No Hit |
| TTCACAGTGGCTAAGTTCTGC | 22094 | 0.23700810680576065 | No Hit |
| GTAGGTAATCGTCAGGCT | 22032 | 0.23634301661738566 | No Hit |
| TCCCTGAGACCCTTTAACCTGT | 21736 | 0.23316774733095016 | No Hit |
| TGAGGTAGTAGATTGTATAGT | 20922 | 0.22443575679325262 | No Hit |
| GGCTGGTCCGATGGTAGTGGGTTATCAGAACA | 20525 | 0.2201770341354321 | No Hit |
| TAGCTTATCAGACTGATGTTGAC | 20412 | 0.21896485363081314 | No Hit |
| TCAGTGCATCACAGAACTTTGTTT | 19898 | 0.2134510414239624 | No Hit |
| TGAAAGTAGGTAATCGTCAGGCT | 18529 | 0.19876542097419836 | No Hit |
| AAAGTAGGTAATCGTCAGGCT | 18106 | 0.19422778952770445 | No Hit |
| GCTGGTCCGATGGTAGTGGGTTATCAGAACT | 17094 | 0.18337180129164807 | No Hit |
| CAACGGAATCCCAAAAGCAGCTGT | 16924 | 0.18154816690416825 | No Hit |
| TCCTGTACTGAGCTGCCCCGAGT | 16646 | 0.17856598831758355 | No Hit |
| AGTAGGTAATCGTCAGGCT | 16447 | 0.17643126335812187 | No Hit |
| AAGTAGGTAATCGTCAGGCT | 16179 | 0.17355635738256547 | No Hit |
| TACCCTGTAGATCCGAATTTGT | 16154 | 0.1732881758549949 | No Hit |
| GAAAGTAGGTAATCGTCAGGCT | 16127 | 0.17299853980521868 | No Hit |
| TGAGGTAGTAGGTTGTGTGGTT | 15815 | 0.16965163434113806 | No Hit |
| CAACGGAATCCCAAAAGCAGCTT | 15106 | 0.1620460062192369 | No Hit |
| CACCCGTAGAACCGACCTTGCG | 14262 | 0.1529921978484547 | No Hit |
| AGCTCGGTCTGAGGCCCCTCAGT | 13782 | 0.1478431125190999 | No Hit |
| GTGAAAGTAGGTAATCGTCAGGCT | 13622 | 0.14612675074264828 | No Hit |
| TGAGAACTGAATTCCATGGGTTG | 13547 | 0.1453222061599366 | No Hit |
| TATTGCACTTGTCCCGGCCTGTAT | 13153 | 0.14109566528542453 | No Hit |
| TCCTGTACTGAGCTGCCCCGAGA | 12498 | 0.13406930926307578 | No Hit |
| GTGTGAAAGTAGGTAATCGTCAGGCT | 12260 | 0.13151622112060402 | No Hit |
| TGTAAACATCCTACACTCTCAGCT | 11779 | 0.1263564085301464 | No Hit |
| TCCTGTACTGAGCTGCCCCGA | 11628 | 0.1247365921036202 | No Hit |
| CCCCCCACTGCTAAATTTGACTGGCTA | 11618 | 0.12462931949259197 | No Hit |
| CCGTGTGAAAGTAGGTAATCGTCAGGCT | 11500 | 0.12336350268245891 | No Hit |
| TGAGGTAGTAGTTTGTGCTGTT | 11366 | 0.12192604969468068 | No Hit |
| TGAGAACTGAATTCCATGGGTTGT | 11160 | 0.11971623390749926 | No Hit |
| TATTGCACTTGTCCCGGCCTGT | 11007 | 0.11807496295876742 | No Hit |
| TTCAAGTAATCCAGGATAGGC | 10991 | 0.11790332678112225 | No Hit |
| AGTTGGTCCGAGTGTTGTGGGTTATTGTTAA | 10845 | 0.11633714666011016 | No Hit |
| TGTGAAAGTAGGTAATCGTCAGGCT | 10673 | 0.11449205775042469 | No Hit |
| CGTGTGAAAGTAGGTAATCGTCAGGCT | 10237 | 0.10981497190959409 | No Hit |
| CGGAATGTAGCGCAGTTGGTAGC | 9784 | 0.10495552263001548 | No Hit |

## Adapter Content

Produced by FastQC (version 0.11.8)
