# Supplementary material for: Analysis of plasma‐derived small extracellular vesicle characteristics and microRNA cargo following exercise‐induced skeletal muscle damage in men
Source: Physiol Rep. 2024 Sep 20;12(18):e70056. doi: 10.14814/phy2.70056 (PMC11415274; doi:10.14814/phy2.70056)
Supplement: Supplementary file 2 — Data S2: Supporting Information. [file PHY2-12-e70056-s001.zip › Processed Files For Individual Samples/SV18362-0005_S5_R1_001.fastq_CORE_RESULTS_v4.6.2.tgz!/SV18362-0005_S5_R1_001.fastq/SV18362-0005_S5_R1_001.fastq.trim_adapter.fastq.tmp_fastqc.zip!/SV18362-0005_S5_R1_001.fastq.trim_adapter.fastq.tmp_fastqc/fastqc_report.html]

SV18362-0005\_S5\_R1\_001.fastq.trim\_adapter.fastq.tmp FastQC Report 

FastQC Report

Fri 21 Feb 2020  
SV18362-0005\_S5\_R1\_001.fastq.trim\_adapter.fastq.tmp

## Summary

- Basic Statistics
- Per base sequence quality
- Per tile sequence quality
- Per sequence quality scores
- Per base sequence content
- Per sequence GC content
- Per base N content
- Sequence Length Distribution
- Sequence Duplication Levels
- Overrepresented sequences
- Adapter Content

## Basic Statistics

| Measure | Value |
| --- | --- |
| Filename | SV18362-0005\_S5\_R1\_001.fastq.trim\_adapter.fastq.tmp |
| File type | Conventional base calls |
| Encoding | Sanger / Illumina 1.9 |
| Total Sequences | 11270557 |
| Sequences flagged as poor quality | 0 |
| Sequence length | 0-47 |
| %GC | 52 |

## Per base sequence quality

## Per tile sequence quality

## Per sequence quality scores

## Per base sequence content

## Per sequence GC content

## Per base N content

## Sequence Length Distribution

## Sequence Duplication Levels

## Overrepresented sequences

| Sequence | Count | Percentage | Possible Source |
| --- | --- | --- | --- |
| GGCTGGTCCGATGGTAGTGGGTTATCAGAACT | 1433441 | 12.718457481737595 | No Hit |
| TTCAAGTAATCCAGGATAGGCT | 610610 | 5.417744659824709 | No Hit |
| CCCCCCACTGCTAAATTTGACTGGCTT | 432545 | 3.837831617372593 | No Hit |
| TGAGGTAGTAGATTGTATAGTT | 128463 | 1.1398105701430727 | No Hit |
| TCGAGGAGCTCACAGTCTAGT | 109966 | 0.9756926831566531 | No Hit |
| CAACGGAATCCCAAAAGCAGCT | 93013 | 0.8252742078319643 | No Hit |
| TGAGGGGCAGAGAGCGAGACTTT | 91066 | 0.8079991077637069 | No Hit |
| CAACGGAATCCCAAAAGCAGCTG | 85392 | 0.7576555444420361 | No Hit |
| CCCCCACTGCTAAATTTGACTGGCTT | 75028 | 0.6656991309302637 | No Hit |
|  | 67062 | 0.5950193943387181 | No Hit |
| TGTAAACATCCCCGACTGGAAGCT | 63454 | 0.5630067795229642 | No Hit |
| GGCTGGTCCGATGGTAGTGGGTTATCAGAAC | 49057 | 0.4352668639180832 | No Hit |
| TGAGGTAGTAGGTTGTATAGTT | 48084 | 0.4266337502219278 | No Hit |
| CCCCCCACTGCTAAATTTGACTGGCTTT | 44751 | 0.3970611212915209 | No Hit |
| TAGCTTATCAGACTGATGTTGACT | 40045 | 0.3553063082862719 | No Hit |
| GCATTGGTGGTTCAGTGGTAGAATTCTCGCCT | 38668 | 0.343088633507643 | No Hit |
| TGTAAACATCCTTGACTGGAAGCT | 35590 | 0.3157785369436489 | No Hit |
| GGCTGGTCCGATGGTAGTGGGTTATCAGAACTT | 34361 | 0.30487401820513393 | No Hit |
| TGAGAACTGAATTCCATGGGTT | 31902 | 0.2830561080521575 | No Hit |
| TCGAGGAGCTCACAGTCTAGTA | 30084 | 0.26692558318102644 | No Hit |
| AAATGATACGGCGACCACCGA | 29251 | 0.25953464411741145 | No Hit |
| TCCTGTACTGAGCTGCCCCGAG | 24977 | 0.22161282712114405 | No Hit |
| TACCCTGTAGAACCGAATTTGT | 24760 | 0.2196874564407065 | No Hit |
| TGAGGGGCAGAGAGCGAGACTTTT | 24381 | 0.2163247122569009 | No Hit |
| TGAGGTAGTAGATTGTATAGTTT | 24122 | 0.21402668918670126 | No Hit |
| TGAGAACTGAATTCCATAGGCTGT | 23910 | 0.21214568188599728 | No Hit |
| TTCACAGTGGCTAAGTTCTGC | 22094 | 0.1960329023667597 | No Hit |
| GTAGGTAATCGTCAGGCT | 22032 | 0.19548279645806324 | No Hit |
| TCCCTGAGACCCTTTAACCTGT | 21736 | 0.1928564843778351 | No Hit |
| TGAGGTAGTAGATTGTATAGT | 20922 | 0.18563412615720767 | No Hit |
| GGCTGGTCCGATGGTAGTGGGTTATCAGAACA | 20525 | 0.18211167380636112 | No Hit |
| TAGCTTATCAGACTGATGTTGAC | 20412 | 0.18110906142438213 | No Hit |
| TCAGTGCATCACAGAACTTTGTTT | 19898 | 0.17654850598776972 | No Hit |
| TGAAAGTAGGTAATCGTCAGGCT | 18529 | 0.1644018126167145 | No Hit |
| AAAGTAGGTAATCGTCAGGCT | 18106 | 0.16064867069125333 | No Hit |
| GCTGGTCCGATGGTAGTGGGTTATCAGAACT | 17094 | 0.15166952263317598 | No Hit |
| CAACGGAATCCCAAAAGCAGCTGT | 16924 | 0.15016116772223412 | No Hit |
| TCCTGTACTGAGCTGCCCCGAGT | 16646 | 0.14769456380904689 | No Hit |
| AGTAGGTAATCGTCAGGCT | 16447 | 0.14592890129565025 | No Hit |
| AAGTAGGTAATCGTCAGGCT | 16179 | 0.14355102414193016 | No Hit |
| TACCCTGTAGATCCGAATTTGT | 16154 | 0.14332920724326226 | No Hit |
| GAAAGTAGGTAATCGTCAGGCT | 16127 | 0.14308964499270088 | No Hit |
| TGAGGTAGTAGGTTGTGTGGTT | 15815 | 0.14032137009732526 | No Hit |
| CAACGGAATCCCAAAAGCAGCTT | 15106 | 0.1340306428511031 | No Hit |
| CACCCGTAGAACCGACCTTGCG | 14262 | 0.1265421043520742 | No Hit |
| AATGATACGGCGACCACCGA | 14106 | 0.12515796690438635 | No Hit |
| AGCTCGGTCTGAGGCCCCTCAGT | 13782 | 0.12228321989765013 | No Hit |
| GTGAAAGTAGGTAATCGTCAGGCT | 13622 | 0.12086359174617546 | No Hit |
| TGAGAACTGAATTCCATGGGTTG | 13547 | 0.1201981410501717 | No Hit |
| TATTGCACTTGTCCCGGCCTGTAT | 13153 | 0.11670230672716529 | No Hit |
| TCCTGTACTGAGCTGCCCCGAGA | 12498 | 0.11089070398206584 | No Hit |
| GTGTGAAAGTAGGTAATCGTCAGGCT | 12260 | 0.10877900710674726 | No Hit |
| TGTAAACATCCTACACTCTCAGCT | 11779 | 0.10451124997637651 | No Hit |
| TCCTGTACTGAGCTGCCCCGA | 11628 | 0.10317147590842228 | No Hit |
| CCCCCCACTGCTAAATTTGACTGGCTA | 11618 | 0.10308274914895511 | No Hit |
| CCGTGTGAAAGTAGGTAATCGTCAGGCT | 11500 | 0.10203577338724253 | No Hit |
| TGAGGTAGTAGTTTGTGCTGTT | 11366 | 0.10084683481038248 | No Hit |

## Adapter Content

Produced by FastQC (version 0.11.8)
