# Supplementary material for: Analysis of plasma‐derived small extracellular vesicle characteristics and microRNA cargo following exercise‐induced skeletal muscle damage in men
Source: Physiol Rep. 2024 Sep 20;12(18):e70056. doi: 10.14814/phy2.70056 (PMC11415274; doi:10.14814/phy2.70056)
Supplement: Supplementary file 2 — Data S2: Supporting Information. [file PHY2-12-e70056-s001.zip › Processed Files For Individual Samples/SV18362-0006_S6_R1_001.fastq_CORE_RESULTS_v4.6.2.tgz!/SV18362-0006_S6_R1_001.fastq/SV18362-0006_S6_R1_001.fastq.clipped.trimmed.filtered_fastqc.zip!/SV18362-0006_S6_R1_001.fastq.clipped.trimmed.filtered_fastqc/fastqc_report.html]

SV18362-0006\_S6\_R1\_001.fastq.clipped.trimmed.filtered.fastq.gz FastQC Report 

FastQC Report

Fri 21 Feb 2020  
SV18362-0006\_S6\_R1\_001.fastq.clipped.trimmed.filtered.fastq.gz

## Summary

- Basic Statistics
- Per base sequence quality
- Per tile sequence quality
- Per sequence quality scores
- Per base sequence content
- Per sequence GC content
- Per base N content
- Sequence Length Distribution
- Sequence Duplication Levels
- Overrepresented sequences
- Adapter Content

## Basic Statistics

| Measure | Value |
| --- | --- |
| Filename | SV18362-0006\_S6\_R1\_001.fastq.clipped.trimmed.filtered.fastq.gz |
| File type | Conventional base calls |
| Encoding | Sanger / Illumina 1.9 |
| Total Sequences | 11460769 |
| Sequences flagged as poor quality | 0 |
| Sequence length | 18-47 |
| %GC | 52 |

## Per base sequence quality

## Per tile sequence quality

## Per sequence quality scores

## Per base sequence content

## Per sequence GC content

## Per base N content

## Sequence Length Distribution

## Sequence Duplication Levels

## Overrepresented sequences

| Sequence | Count | Percentage | Possible Source |
| --- | --- | --- | --- |
| GGCTGGTCCGATGGTAGTGGGTTATCAGAACT | 1713094 | 14.947461204392132 | No Hit |
| CCCCCCACTGCTAAATTTGACTGGCTT | 663942 | 5.793171470430998 | No Hit |
| TTCAAGTAATCCAGGATAGGCT | 427806 | 3.7327861681881904 | No Hit |
| CCCCCACTGCTAAATTTGACTGGCTT | 133326 | 1.1633250787970684 | No Hit |
| TGAGGTAGTAGATTGTATAGTT | 98686 | 0.8610765996592376 | No Hit |
| GGCTGGTCCGATGGTAGTGGGTTATCAGAAC | 89691 | 0.7825914648484757 | No Hit |
| TGAGGGGCAGAGAGCGAGACTTT | 78521 | 0.6851285459117098 | No Hit |
| TCGAGGAGCTCACAGTCTAGT | 77682 | 0.6778079202189661 | No Hit |
| CAACGGAATCCCAAAAGCAGCTG | 67396 | 0.5880582707844474 | No Hit |
| CAACGGAATCCCAAAAGCAGCT | 67138 | 0.5858071129432938 | No Hit |
| CCCCCCACTGCTAAATTTGACTGGCTTT | 61634 | 0.5377824123320172 | No Hit |
| GTAGGTAATCGTCAGGCT | 50136 | 0.43745755629486993 | No Hit |
| TGTAAACATCCCCGACTGGAAGCT | 48731 | 0.42519834402037066 | No Hit |
| AAATGATACGGCGACCACCGA | 48605 | 0.42409894135376086 | No Hit |
| AAAGTAGGTAATCGTCAGGCT | 40192 | 0.35069199981257804 | No Hit |
| TGAAAGTAGGTAATCGTCAGGCT | 39649 | 0.3459540978445687 | No Hit |
| AGTAGGTAATCGTCAGGCT | 36470 | 0.31821599405764134 | No Hit |
| AAGTAGGTAATCGTCAGGCT | 36181 | 0.31569434825882975 | No Hit |
| GGCTGGTCCGATGGTAGTGGGTTATCAGAACTT | 35930 | 0.31350426834359896 | No Hit |
| GAAAGTAGGTAATCGTCAGGCT | 34824 | 0.30385395604780097 | No Hit |
| GGCTGGTCCGATGGTAGTGGGTTATCAGAACA | 32786 | 0.2860715541862854 | No Hit |
| TGAGGTAGTAGGTTGTATAGTT | 32287 | 0.28171757060979064 | No Hit |
| TGAGAACTGAATTCCATGGGTT | 31278 | 0.2729136238589226 | No Hit |
| GTGAAAGTAGGTAATCGTCAGGCT | 27477 | 0.23974831008285744 | No Hit |
| TCCTGTACTGAGCTGCCCCGAG | 27372 | 0.23883214119401588 | No Hit |
| TGTAAACATCCTTGACTGGAAGCT | 27007 | 0.23564736362804276 | No Hit |
| AATGATACGGCGACCACCGA | 23452 | 0.20462850267726362 | No Hit |
| CCCCCCACTGCTAAATTTGACTGGCTA | 22506 | 0.19637425725970045 | No Hit |
| GTGTGAAAGTAGGTAATCGTCAGGCT | 22452 | 0.19590308468829623 | No Hit |
| TCGAGGAGCTCACAGTCTAGTA | 22449 | 0.1958769084343293 | No Hit |
| TGTGAAAGTAGGTAATCGTCAGGCT | 21520 | 0.1877709951225786 | No Hit |
| TAGCTTATCAGACTGATGTTGACT | 20879 | 0.18217800219165048 | No Hit |
| CAGAGTTCTACAGTCCGACGATC | 20219 | 0.176419226318932 | Illumina DpnII expression Sequencing Primer (100% over 23bp) |
| CCGTGTGAAAGTAGGTAATCGTCAGGCT | 19869 | 0.1733653300227934 | No Hit |
| TGAGGGGCAGAGAGCGAGACTTTT | 19714 | 0.17201289023450347 | No Hit |
| CGTGTGAAAGTAGGTAATCGTCAGGCT | 18829 | 0.1642908953142673 | No Hit |
| GCCCAGGTCGGGGAGCCA | 17435 | 0.15212766263764674 | No Hit |
| GCTGGTCCGATGGTAGTGGGTTATCAGAACT | 17048 | 0.14875092587591635 | No Hit |
| AGAGTTCTACAGTCCGACGATC | 17011 | 0.14842808541032454 | Illumina DpnII expression Sequencing Primer (100% over 22bp) |
| TGAGAACTGAATTCCATAGGCTGT | 16451 | 0.14354185133650282 | No Hit |
| TCCTGTACTGAGCTGCCCCGAGT | 15766 | 0.13756494001406014 | No Hit |
| TGAGGTAGTAGATTGTATAGTTT | 15427 | 0.13460702331580018 | No Hit |
| GCATTGGTGGTTCAGTGGTAGAATTCTCGCCT | 15096 | 0.131718909961452 | No Hit |
| TGAGAACTGAATTCCATGGGTTG | 14796 | 0.12910128456476175 | No Hit |
| TCCTGTACTGAGCTGCCCCGA | 14739 | 0.1286039357393906 | No Hit |
| TTCTACAGTCCGACGATC | 14373 | 0.12541043275542854 | Illumina DpnII expression Sequencing Primer (100% over 18bp) |
| TGAGGTAGTAGATTGTATAGT | 14223 | 0.12410162005708342 | No Hit |
| GTTCTACAGTCCGACGATC | 13757 | 0.1200355752742246 | Illumina DpnII expression Sequencing Primer (100% over 19bp) |
| AAAATGATACGGCGACCACCGA | 13277 | 0.11584737463952027 | No Hit |
| AGCTCGGTCTGAGGCCCCTCAGT | 13025 | 0.11364856930630048 | No Hit |
| TAGCTTATCAGACTGATGTTGAC | 12787 | 0.11157191982492623 | No Hit |
| TCAGTGCATCACAGAACTTTGTTT | 12445 | 0.10858782687269937 | No Hit |
| TGAACTGAAGAGTTTGATCCTGGCTC | 12177 | 0.10624941485165612 | No Hit |
| CCCGTGTGAAAGTAGGTAATCGTCAGGCT | 12138 | 0.10590912355008639 | No Hit |
| CAACGGAATCCCAAAAGCAGCTGT | 12104 | 0.1056124593384615 | No Hit |
| TCCTGTACTGAGCTGCCCCGAGA | 11875 | 0.10361433861898796 | No Hit |
| TCCCTGAGACCCTTTAACCTGT | 11807 | 0.10302101019573817 | No Hit |
| TTCACAGTGGCTAAGTTCTGC | 11711 | 0.10218337006879731 | No Hit |

## Adapter Content

Produced by FastQC (version 0.11.8)
