# Supplementary material for: Analysis of plasma‐derived small extracellular vesicle characteristics and microRNA cargo following exercise‐induced skeletal muscle damage in men
Source: Physiol Rep. 2024 Sep 20;12(18):e70056. doi: 10.14814/phy2.70056 (PMC11415274; doi:10.14814/phy2.70056)
Supplement: Supplementary file 2 — Data S2: Supporting Information. [file PHY2-12-e70056-s001.zip › Processed Files For Individual Samples/SV18362-0006_S6_R1_001.fastq_CORE_RESULTS_v4.6.2.tgz!/SV18362-0006_S6_R1_001.fastq/SV18362-0006_S6_R1_001.fastq.clipped.trimmed.filtered.noRiboRNA_fastqc.zip!/SV18362-0006_S6_R1_001.fastq.clipped.trimmed.filtered.noRiboRNA_fastqc/fastqc_report.html]

SV18362-0006\_S6\_R1\_001.fastq.clipped.trimmed.filtered.noRiboRNA.fastq.gz FastQC Report 

FastQC Report

Fri 21 Feb 2020  
SV18362-0006\_S6\_R1\_001.fastq.clipped.trimmed.filtered.noRiboRNA.fastq.gz

## Summary

- Basic Statistics
- Per base sequence quality
- Per tile sequence quality
- Per sequence quality scores
- Per base sequence content
- Per sequence GC content
- Per base N content
- Sequence Length Distribution
- Sequence Duplication Levels
- Overrepresented sequences
- Adapter Content

## Basic Statistics

| Measure | Value |
| --- | --- |
| Filename | SV18362-0006\_S6\_R1\_001.fastq.clipped.trimmed.filtered.noRiboRNA.fastq.gz |
| File type | Conventional base calls |
| Encoding | Sanger / Illumina 1.9 |
| Total Sequences | 10894169 |
| Sequences flagged as poor quality | 0 |
| Sequence length | 18-47 |
| %GC | 52 |

## Per base sequence quality

## Per tile sequence quality

## Per sequence quality scores

## Per base sequence content

## Per sequence GC content

## Per base N content

## Sequence Length Distribution

## Sequence Duplication Levels

## Overrepresented sequences

| Sequence | Count | Percentage | Possible Source |
| --- | --- | --- | --- |
| GGCTGGTCCGATGGTAGTGGGTTATCAGAACT | 1713094 | 15.724870800150063 | No Hit |
| CCCCCCACTGCTAAATTTGACTGGCTT | 663942 | 6.094471271741791 | No Hit |
| TTCAAGTAATCCAGGATAGGCT | 427806 | 3.9269264135704156 | No Hit |
| CCCCCACTGCTAAATTTGACTGGCTT | 133326 | 1.2238290043049636 | No Hit |
| TGAGGTAGTAGATTGTATAGTT | 98686 | 0.9058607407320375 | No Hit |
| GGCTGGTCCGATGGTAGTGGGTTATCAGAAC | 89691 | 0.8232936353383171 | No Hit |
| TGAGGGGCAGAGAGCGAGACTTT | 78521 | 0.7207617212473939 | No Hit |
| TCGAGGAGCTCACAGTCTAGT | 77682 | 0.7130603536625878 | No Hit |
| CAACGGAATCCCAAAAGCAGCTG | 67396 | 0.6186428721639989 | No Hit |
| CAACGGAATCCCAAAAGCAGCT | 67138 | 0.6162746327875032 | No Hit |
| CCCCCCACTGCTAAATTTGACTGGCTTT | 61634 | 0.5657521927555925 | No Hit |
| GTAGGTAATCGTCAGGCT | 50136 | 0.460209493720907 | No Hit |
| TGTAAACATCCCCGACTGGAAGCT | 48731 | 0.4473126862636333 | No Hit |
| AAAGTAGGTAATCGTCAGGCT | 40192 | 0.3689313062795336 | No Hit |
| TGAAAGTAGGTAATCGTCAGGCT | 39649 | 0.36394698852202495 | No Hit |
| AGTAGGTAATCGTCAGGCT | 36470 | 0.33476624054574516 | No Hit |
| AAGTAGGTAATCGTCAGGCT | 36181 | 0.3321134452751743 | No Hit |
| GGCTGGTCCGATGGTAGTGGGTTATCAGAACTT | 35930 | 0.3298094604554051 | No Hit |
| GAAAGTAGGTAATCGTCAGGCT | 34824 | 0.31965724049259747 | No Hit |
| GGCTGGTCCGATGGTAGTGGGTTATCAGAACA | 32786 | 0.30094998526275846 | No Hit |
| TGAGGTAGTAGGTTGTATAGTT | 32287 | 0.29636955329038867 | No Hit |
| TGAGAACTGAATTCCATGGGTT | 31278 | 0.28710771789936435 | No Hit |
| GTGAAAGTAGGTAATCGTCAGGCT | 27477 | 0.25221749359680395 | No Hit |
| TCCTGTACTGAGCTGCCCCGAG | 27372 | 0.2512536752459045 | No Hit |
| TGTAAACATCCTTGACTGGAAGCT | 27007 | 0.24790325907373018 | No Hit |
| CCCCCCACTGCTAAATTTGACTGGCTA | 22506 | 0.20658757909850672 | No Hit |
| GTGTGAAAGTAGGTAATCGTCAGGCT | 22452 | 0.20609190108947273 | No Hit |
| TCGAGGAGCTCACAGTCTAGTA | 22449 | 0.20606436342230416 | No Hit |
| TGTGAAAGTAGGTAATCGTCAGGCT | 21520 | 0.19753686582244132 | No Hit |
| TAGCTTATCAGACTGATGTTGACT | 20879 | 0.19165298427075989 | No Hit |
| CCGTGTGAAAGTAGGTAATCGTCAGGCT | 19869 | 0.18238196965734604 | No Hit |
| TGAGGGGCAGAGAGCGAGACTTTT | 19714 | 0.18095919018697065 | No Hit |
| CGTGTGAAAGTAGGTAATCGTCAGGCT | 18829 | 0.17283557837224664 | No Hit |
| GCCCAGGTCGGGGAGCCA | 17435 | 0.16003974236125765 | No Hit |
| GCTGGTCCGATGGTAGTGGGTTATCAGAACT | 17048 | 0.15648738329651393 | No Hit |
| TGAGAACTGAATTCCATAGGCTGT | 16451 | 0.1510073875299713 | No Hit |
| TCCTGTACTGAGCTGCCCCGAGT | 15766 | 0.14471962019315104 | No Hit |
| TGAGGTAGTAGATTGTATAGTTT | 15427 | 0.14160786380310422 | No Hit |
| GCATTGGTGGTTCAGTGGTAGAATTCTCGCCT | 15096 | 0.13856954119217355 | No Hit |
| TGAGAACTGAATTCCATGGGTTG | 14796 | 0.13581577447531795 | No Hit |
| TCCTGTACTGAGCTGCCCCGA | 14739 | 0.1352925587991154 | No Hit |
| TGAGGTAGTAGATTGTATAGT | 14223 | 0.13055608004612376 | No Hit |
| AGCTCGGTCTGAGGCCCCTCAGT | 13025 | 0.1195593716234804 | No Hit |
| TAGCTTATCAGACTGATGTTGAC | 12787 | 0.11737471669477498 | No Hit |
| TCAGTGCATCACAGAACTTTGTTT | 12445 | 0.11423542263755959 | No Hit |
| TGAACTGAAGAGTTTGATCCTGGCTC | 12177 | 0.1117753910371686 | No Hit |
| CCCGTGTGAAAGTAGGTAATCGTCAGGCT | 12138 | 0.11141740136397736 | No Hit |
| CAACGGAATCCCAAAAGCAGCTGT | 12104 | 0.11110530780273373 | No Hit |
| TCCTGTACTGAGCTGCCCCGAGA | 11875 | 0.10900326587553397 | No Hit |
| TCCCTGAGACCCTTTAACCTGT | 11807 | 0.10837907875304668 | No Hit |
| TTCACAGTGGCTAAGTTCTGC | 11711 | 0.10749787340365291 | No Hit |
| TGAGGTAGTAGTTTGTGCTGTT | 11417 | 0.10479918202113442 | No Hit |
| TTCGACTCCTATCTCCGGCACCA | 11345 | 0.10413827800908908 | No Hit |
| TGAACTGAAGAGTTTGATCCT | 11030 | 0.1012468229563907 | No Hit |

## Adapter Content

Produced by FastQC (version 0.11.8)
