# Supplementary material for: Analysis of plasma‐derived small extracellular vesicle characteristics and microRNA cargo following exercise‐induced skeletal muscle damage in men
Source: Physiol Rep. 2024 Sep 20;12(18):e70056. doi: 10.14814/phy2.70056 (PMC11415274; doi:10.14814/phy2.70056)
Supplement: Supplementary file 2 — Data S2: Supporting Information. [file PHY2-12-e70056-s001.zip › Processed Files For Individual Samples/SV18362-0006_S6_R1_001.fastq_CORE_RESULTS_v4.6.2.tgz!/SV18362-0006_S6_R1_001.fastq/SV18362-0006_S6_R1_001.fastq.trim_adapter.fastq.tmp_fastqc.zip!/SV18362-0006_S6_R1_001.fastq.trim_adapter.fastq.tmp_fastqc/fastqc_report.html]

SV18362-0006\_S6\_R1\_001.fastq.trim\_adapter.fastq.tmp FastQC Report 

FastQC Report

Fri 21 Feb 2020  
SV18362-0006\_S6\_R1\_001.fastq.trim\_adapter.fastq.tmp

## Summary

- Basic Statistics
- Per base sequence quality
- Per tile sequence quality
- Per sequence quality scores
- Per base sequence content
- Per sequence GC content
- Per base N content
- Sequence Length Distribution
- Sequence Duplication Levels
- Overrepresented sequences
- Adapter Content

## Basic Statistics

| Measure | Value |
| --- | --- |
| Filename | SV18362-0006\_S6\_R1\_001.fastq.trim\_adapter.fastq.tmp |
| File type | Conventional base calls |
| Encoding | Sanger / Illumina 1.9 |
| Total Sequences | 14500844 |
| Sequences flagged as poor quality | 0 |
| Sequence length | 0-47 |
| %GC | 53 |

## Per base sequence quality

## Per tile sequence quality

## Per sequence quality scores

## Per base sequence content

## Per sequence GC content

## Per base N content

## Sequence Length Distribution

## Sequence Duplication Levels

## Overrepresented sequences

| Sequence | Count | Percentage | Possible Source |
| --- | --- | --- | --- |
| GGCTGGTCCGATGGTAGTGGGTTATCAGAACT | 1713094 | 11.813753737368666 | No Hit |
| CCCCCCACTGCTAAATTTGACTGGCTT | 663942 | 4.578643836179467 | No Hit |
| TTCAAGTAATCCAGGATAGGCT | 427806 | 2.9502144840672724 | No Hit |
| CCCCCACTGCTAAATTTGACTGGCTT | 133326 | 0.9194361376482638 | No Hit |
|  | 130648 | 0.9009682470896176 | No Hit |
| TGAGGTAGTAGATTGTATAGTT | 98686 | 0.6805534905416539 | No Hit |
| GGCTGGTCCGATGGTAGTGGGTTATCAGAAC | 89691 | 0.6185226184075906 | No Hit |
| TGAGGGGCAGAGAGCGAGACTTT | 78521 | 0.5414926193261578 | No Hit |
| TCGAGGAGCTCACAGTCTAGT | 77682 | 0.5357067492071496 | No Hit |
| CAACGGAATCCCAAAAGCAGCTG | 67396 | 0.4647729470091534 | No Hit |
| CAACGGAATCCCAAAAGCAGCT | 67138 | 0.4629937402264309 | No Hit |
| CCCCCCACTGCTAAATTTGACTGGCTTT | 61634 | 0.4250373288616856 | No Hit |
| GTAGGTAATCGTCAGGCT | 50136 | 0.3457453924750863 | No Hit |
| TGTAAACATCCCCGACTGGAAGCT | 48731 | 0.3360563012746017 | No Hit |
| AAATGATACGGCGACCACCGA | 48605 | 0.3351873863342023 | No Hit |
| AAAGTAGGTAATCGTCAGGCT | 40192 | 0.2771700736867454 | No Hit |
| TGAAAGTAGGTAATCGTCAGGCT | 39649 | 0.2734254640626435 | No Hit |
| AGTAGGTAATCGTCAGGCT | 36470 | 0.251502602193362 | No Hit |
| AAGTAGGTAATCGTCAGGCT | 36181 | 0.24950961475070002 | No Hit |
| GGCTGGTCCGATGGTAGTGGGTTATCAGAACTT | 35930 | 0.247778681020222 | No Hit |
| GAAAGTAGGTAATCGTCAGGCT | 34824 | 0.24015153876560563 | No Hit |
| GGCTGGTCCGATGGTAGTGGGTTATCAGAACA | 32786 | 0.2260971844121625 | No Hit |
| TGAGGTAGTAGGTTGTATAGTT | 32287 | 0.22265600540216832 | No Hit |
| TGAGAACTGAATTCCATGGGTT | 31278 | 0.21569778972865303 | No Hit |
| GTGAAAGTAGGTAATCGTCAGGCT | 27477 | 0.18948552235993987 | No Hit |
| TCCTGTACTGAGCTGCCCCGAG | 27372 | 0.18876142657627376 | No Hit |
| TGTAAACATCCTTGACTGGAAGCT | 27007 | 0.18624433170924395 | No Hit |
| TCTACAGTCCGACGATC | 24452 | 0.1686246676400353 | Illumina DpnII expression Sequencing Primer (100% over 17bp) |
| AATGATACGGCGACCACCGA | 23452 | 0.16172851731940568 | No Hit |
| CCCCCCACTGCTAAATTTGACTGGCTA | 22506 | 0.15520475911609008 | No Hit |
| GTGTGAAAGTAGGTAATCGTCAGGCT | 22452 | 0.15483236699877606 | No Hit |
| TCGAGGAGCTCACAGTCTAGTA | 22449 | 0.15481167854781416 | No Hit |
| TGTGAAAGTAGGTAATCGTCAGGCT | 21520 | 0.14840515489994927 | No Hit |
| TAGCTTATCAGACTGATGTTGACT | 20879 | 0.1439847225444257 | No Hit |
| CAGAGTTCTACAGTCCGACGATC | 20219 | 0.13943326333281014 | Illumina DpnII expression Sequencing Primer (100% over 23bp) |
| CCGTGTGAAAGTAGGTAATCGTCAGGCT | 19869 | 0.13701961072058977 | No Hit |
| TGAGGGGCAGAGAGCGAGACTTTT | 19714 | 0.1359507074208922 | No Hit |
| CGTGTGAAAGTAGGTAATCGTCAGGCT | 18829 | 0.12984761438713496 | No Hit |
| TAGGTAATCGTCAGGCT | 18317 | 0.12631678542297262 | No Hit |
| GCCCAGGTCGGGGAGCCA | 17435 | 0.1202343808401773 | No Hit |
| GGTAATCGTCAGGCT | 17275 | 0.11913099678887656 | No Hit |
| GCTGGTCCGATGGTAGTGGGTTATCAGAACT | 17048 | 0.11756557066609365 | No Hit |
| AGAGTTCTACAGTCCGACGATC | 17011 | 0.11731041310423035 | Illumina DpnII expression Sequencing Primer (100% over 22bp) |
| CGCGGGGTGGAGC | 16471 | 0.11358649193109036 | No Hit |
| TGAGAACTGAATTCCATAGGCTGT | 16451 | 0.11344856892467776 | No Hit |
| TCCTGTACTGAGCTGCCCCGAGT | 15766 | 0.10872470595504648 | No Hit |
| TGAGGTAGTAGATTGTATAGTTT | 15427 | 0.10638691099635303 | No Hit |
| GCATTGGTGGTTCAGTGGTAGAATTCTCGCCT | 15096 | 0.10410428524022462 | No Hit |
| TGAGAACTGAATTCCATGGGTTG | 14796 | 0.10203544014403575 | No Hit |
| TCCTGTACTGAGCTGCCCCGA | 14739 | 0.10164235957575987 | No Hit |

## Adapter Content

Produced by FastQC (version 0.11.8)
