# Supplementary material for: Analysis of plasma‐derived small extracellular vesicle characteristics and microRNA cargo following exercise‐induced skeletal muscle damage in men
Source: Physiol Rep. 2024 Sep 20;12(18):e70056. doi: 10.14814/phy2.70056 (PMC11415274; doi:10.14814/phy2.70056)
Supplement: Supplementary file 2 — Data S2: Supporting Information. [file PHY2-12-e70056-s001.zip › Processed Files For Individual Samples/SV18362-0002_S2_R1_001.fastq_CORE_RESULTS_v4.6.2/SV18362-0002_S2_R1_001.fastq/SV18362-0002_S2_R1_001.fastq.trim_adapter.fastq.tmp_fastqc.zip!/SV18362-0002_S2_R1_001.fastq.trim_adapter.fastq.tmp_fastqc/fastqc_report.html]

SV18362-0002\_S2\_R1\_001.fastq.trim\_adapter.fastq.tmp FastQC Report 

FastQC Report

Fri 21 Feb 2020  
SV18362-0002\_S2\_R1\_001.fastq.trim\_adapter.fastq.tmp

## Summary

- Basic Statistics
- Per base sequence quality
- Per tile sequence quality
- Per sequence quality scores
- Per base sequence content
- Per sequence GC content
- Per base N content
- Sequence Length Distribution
- Sequence Duplication Levels
- Overrepresented sequences
- Adapter Content

## Basic Statistics

| Measure | Value |
| --- | --- |
| Filename | SV18362-0002\_S2\_R1\_001.fastq.trim\_adapter.fastq.tmp |
| File type | Conventional base calls |
| Encoding | Sanger / Illumina 1.9 |
| Total Sequences | 13312672 |
| Sequences flagged as poor quality | 0 |
| Sequence length | 0-47 |
| %GC | 59 |

## Per base sequence quality

## Per tile sequence quality

## Per sequence quality scores

## Per base sequence content

## Per sequence GC content

## Per base N content

## Sequence Length Distribution

## Sequence Duplication Levels

## Overrepresented sequences

| Sequence | Count | Percentage | Possible Source |
| --- | --- | --- | --- |
| GGCTGGTCCGATGGTAGTGGGTTATCAGAACT | 378941 | 2.8464683874131356 | No Hit |
| GCGGGGCGAAGCCAGAGGA | 273177 | 2.0520072904973548 | No Hit |
| TTCAAGTAATCCAGGATAGGCT | 181957 | 1.366795486285548 | No Hit |
| CCCCCCACTGCTAAATTTGACTGGCTT | 145635 | 1.0939576968470341 | No Hit |
| GCGGGGCGAAGCCAGAGGAA | 119391 | 0.8968222157054572 | No Hit |
| CCCGGCTGGTGCACCA | 65754 | 0.4939203790193284 | No Hit |
| GGCGGACCGGGCCC | 62715 | 0.4710925049456638 | No Hit |
| CCGGGACGTGGCGGCT | 58225 | 0.43736524117772896 | No Hit |
| ACCGGGACGTGGCGGCTGACGGCA | 55072 | 0.4136810401398006 | No Hit |
| ACCGGGACGTGGCGGCT | 51468 | 0.38660908944500394 | No Hit |
| TCCCACTGTCCCTGTCT | 50962 | 0.3828081995860786 | No Hit |
| CCTCCTGGGAAGTCCTCGTGTTGCACCTCT | 47663 | 0.35802729910268954 | No Hit |
| TCCCGGCTGGTGCACCA | 45863 | 0.344506347035366 | No Hit |
| CAGGGACAGTCGGGGGC | 44478 | 0.3341027255835643 | No Hit |
| GCCCGGACCCTGTCGCACCA | 37444 | 0.2812658495604789 | No Hit |
| TGGCGGACCGGGCCC | 37298 | 0.28016915011501825 | No Hit |
| TGAGGGGCAGAGAGCGAGACTTT | 33865 | 0.2543816898666173 | No Hit |
| TCGAGGAGCTCACAGTCTAGT | 33710 | 0.25321738566082 | No Hit |
| TGAGGTAGTAGATTGTATAGTT | 33656 | 0.25281175709880027 | No Hit |
| CCGGGACGTGGCGGCTGACGGCA | 33404 | 0.25091882380937497 | No Hit |
|  | 32508 | 0.24418839433586284 | No Hit |
| CAACGGAATCCCAAAAGCAGCT | 29814 | 0.22395203607510197 | No Hit |
| GCCGGTCGGGGGACGGATTGGGA | 29683 | 0.22296801123020232 | No Hit |
| GAGGTACGGGGATCG | 29067 | 0.21834084096716272 | No Hit |
| CCCGGACCCTGTCGCACCA | 28811 | 0.21641786111758782 | No Hit |
| CCCGGCTGGTGCACC | 28096 | 0.2110470384908454 | No Hit |
| ACCGGGACGTGGCGGCTGA | 27365 | 0.20555602962350458 | No Hit |
| CCCACTGTCCCTGTCT | 26728 | 0.20077111491967953 | No Hit |
| CCCCCACTGCTAAATTTGACTGGCTT | 26228 | 0.19701529490097855 | No Hit |
| GAGGTACGGGGATC | 25236 | 0.18956374798387582 | No Hit |
| CAACGGAATCCCAAAAGCAGCTG | 24666 | 0.1852821131625567 | No Hit |
| ACAGGGACAGTCGGGGGC | 24665 | 0.1852746015225193 | No Hit |
| TTCCCGGCTGGTGCACCA | 24640 | 0.18508681052158424 | No Hit |
| TCCCGGCTGGTGCACC | 22816 | 0.17138557909336308 | No Hit |
| TGTAAACATCCCCGACTGGAAGCT | 20070 | 0.15075861555065728 | No Hit |
| GCGGACCGGGCCC | 19497 | 0.14645444580922598 | No Hit |
| CCTGTCGGGACCCGA | 18703 | 0.14049020361952883 | No Hit |
| CGCGACGGGGTA | 18328 | 0.13767333860550307 | No Hit |
| CGGCGGGAGTAACTA | 18194 | 0.13666677884049122 | No Hit |
| ACCACGTGGGAATTCTTGATGCTGTATGT | 17882 | 0.13432314714882182 | No Hit |
| CCGGGACGTGGCGGCTGACGGCAA | 17619 | 0.1323475858189851 | No Hit |
| GATTCCCACTGTCCCTGTCT | 17267 | 0.12970348852581962 | No Hit |
| ATTCCCACTGTCCCTGTCT | 17247 | 0.12955325572507156 | No Hit |
| GCCCCGTCGTGCCCGGACCCT | 16979 | 0.12754013619504784 | No Hit |
| ACCGGGACGTGGCGGCTGACGGCAA | 16970 | 0.12747253143471124 | No Hit |
| AGGCCGAGGACTTACC | 16536 | 0.12421247965847877 | No Hit |
| TTCCTGAACCGGGACGTGGCGGCTGA | 16527 | 0.12414487489814216 | No Hit |
| CCCCCCACTGCTAAATTTGACTGGCTTT | 16349 | 0.12280780297148461 | No Hit |
| CAGGGACAGTCGGGGGCA | 15886 | 0.1193299136341675 | No Hit |
| GGCGGACCGGGCCCA | 15692 | 0.11787265546691153 | No Hit |
| TCCTGTACTGAGCTGCCCCGAG | 15131 | 0.11365862540592903 | No Hit |
| GAGGCACGGGGTTCG | 15006 | 0.11271967040125377 | No Hit |
| CTGGCGATGCGGGA | 14981 | 0.11253187940031874 | No Hit |
| CCGGCTGGTGCACC | 14893 | 0.11187085507702735 | No Hit |
| CTCCGCTGGCACCTT | 14483 | 0.10879108266169256 | No Hit |
| AAATGATACGGCGACCACCGA | 14381 | 0.10802489537787756 | No Hit |
| CGTCGGCGGGGGCCCCGGAA | 14339 | 0.10770940649630668 | No Hit |
| ACGAGACCTCAGCCTGCTA | 14233 | 0.10691317265234207 | No Hit |
| CGCCCTCGACCTATTCTC | 14039 | 0.1054559144850861 | No Hit |
| GCGCCCGTAGCTCAACGGATAGAGCACTTGAC | 13941 | 0.1047197737614207 | No Hit |
| CCCAGGGCGCGA | 13836 | 0.10393105155749348 | No Hit |
| CCTGTCGGGACCCGAAA | 13728 | 0.10311979443345409 | No Hit |
| GCACCGGTCGGCTCGTCCCTTC | 13562 | 0.10187286218724535 | No Hit |
| TGCCCGGACCCTGTCGCACCA | 13449 | 0.10102404686301893 | No Hit |
| CTCCGCTGGCACCTTA | 13379 | 0.10049823206040079 | No Hit |

## Adapter Content

Produced by FastQC (version 0.11.8)
