# Supplementary material for: Analysis of plasma‐derived small extracellular vesicle characteristics and microRNA cargo following exercise‐induced skeletal muscle damage in men
Source: Physiol Rep. 2024 Sep 20;12(18):e70056. doi: 10.14814/phy2.70056 (PMC11415274; doi:10.14814/phy2.70056)
Supplement: Supplementary file 2 — Data S2: Supporting Information. [file PHY2-12-e70056-s001.zip › Processed Files For Individual Samples/SV18362-0004_S4_R1_001.fastq_CORE_RESULTS_v4.6.2.tgz!/SV18362-0004_S4_R1_001.fastq/SV18362-0004_S4_R1_001.fastq.clipped.trimmed.filtered_fastqc.zip!/SV18362-0004_S4_R1_001.fastq.clipped.trimmed.filtered_fastqc/fastqc_report.html]

SV18362-0004\_S4\_R1\_001.fastq.clipped.trimmed.filtered.fastq.gz FastQC Report 

FastQC Report

Fri 21 Feb 2020  
SV18362-0004\_S4\_R1\_001.fastq.clipped.trimmed.filtered.fastq.gz

## Summary

- Basic Statistics
- Per base sequence quality
- Per tile sequence quality
- Per sequence quality scores
- Per base sequence content
- Per sequence GC content
- Per base N content
- Sequence Length Distribution
- Sequence Duplication Levels
- Overrepresented sequences
- Adapter Content

## Basic Statistics

| Measure | Value |
| --- | --- |
| Filename | SV18362-0004\_S4\_R1\_001.fastq.clipped.trimmed.filtered.fastq.gz |
| File type | Conventional base calls |
| Encoding | Sanger / Illumina 1.9 |
| Total Sequences | 10820635 |
| Sequences flagged as poor quality | 0 |
| Sequence length | 18-47 |
| %GC | 51 |

## Per base sequence quality

## Per tile sequence quality

## Per sequence quality scores

## Per base sequence content

## Per sequence GC content

## Per base N content

## Sequence Length Distribution

## Sequence Duplication Levels

## Overrepresented sequences

| Sequence | Count | Percentage | Possible Source |
| --- | --- | --- | --- |
| GGCTGGTCCGATGGTAGTGGGTTATCAGAACT | 1133549 | 10.475808489982334 | No Hit |
| CCCCCCACTGCTAAATTTGACTGGCTT | 509001 | 4.703984562828337 | No Hit |
| TTCAAGTAATCCAGGATAGGCT | 467963 | 4.324727707754674 | No Hit |
| CCCCCACTGCTAAATTTGACTGGCTT | 88901 | 0.8215876424997239 | No Hit |
| TGAGGTAGTAGATTGTATAGTT | 82292 | 0.760509896138258 | No Hit |
| TCGAGGAGCTCACAGTCTAGT | 81586 | 0.7539853252604861 | No Hit |
| CAACGGAATCCCAAAAGCAGCT | 75198 | 0.6949499728990027 | No Hit |
| CAACGGAATCCCAAAAGCAGCTG | 64894 | 0.5997245078500476 | No Hit |
| GGCTGGTCCGATGGTAGTGGGTTATCAGAAC | 59518 | 0.550041656520158 | No Hit |
| TGAGGGGCAGAGAGCGAGACTTT | 56010 | 0.5176221173711155 | No Hit |
| TGTAAACATCCCCGACTGGAAGCT | 47263 | 0.4367858263401362 | No Hit |
| CCCCCCACTGCTAAATTTGACTGGCTTT | 45174 | 0.41748012015930674 | No Hit |
| TGGAATGTAAAGAAGTATGGAG | 45135 | 0.41711969768872165 | No Hit |
| GTAGGTAATCGTCAGGCT | 43506 | 0.4020651283404347 | No Hit |
| TACCCTGTAGAACCGAATTTGT | 39444 | 0.36452574178872127 | No Hit |
| TCCTGTACTGAGCTGCCCCGAG | 37594 | 0.34742877844045195 | No Hit |
| AAAGTAGGTAATCGTCAGGCT | 35444 | 0.32755933454922004 | No Hit |
| TGAGGTAGTAGGTTGTATAGTT | 34988 | 0.32334516412391695 | No Hit |
| TGAGAACTGAATTCCATGGGTT | 34249 | 0.31651562038641906 | No Hit |
| TGAAAGTAGGTAATCGTCAGGCT | 33254 | 0.30732022658559316 | No Hit |
| AAATGATACGGCGACCACCGA | 32761 | 0.30276411689332466 | No Hit |
| GAAAGTAGGTAATCGTCAGGCT | 31526 | 0.29135073865812866 | No Hit |
| AGTAGGTAATCGTCAGGCT | 31369 | 0.28989980717397823 | No Hit |
| TCCCTGAGACCCTTTAACCTGT | 30873 | 0.2853159726762801 | No Hit |
| AAGTAGGTAATCGTCAGGCT | 30793 | 0.28457664453149006 | No Hit |
| TGTAAACATCCTTGACTGGAAGCT | 28592 | 0.26423587894795453 | No Hit |
| TGACTAGATCCATACTCATCCA | 26060 | 0.2408361431653503 | No Hit |
| GGCTGGTCCGATGGTAGTGGGTTATCAGAACTT | 25459 | 0.23528194047761522 | No Hit |
| TGAGATCATTGTGAAAGCTGTTT | 24757 | 0.22879433600708274 | No Hit |
| GTGAAAGTAGGTAATCGTCAGGCT | 22385 | 0.20687325651405855 | No Hit |
| GGCTGGTCCGATGGTAGTGGGTTATCAGAACA | 22244 | 0.20557019065886614 | No Hit |
| GAACTATTGACTGGCACTCACAG | 21862 | 0.20203989876749376 | No Hit |
| TCGAGGAGCTCACAGTCTAGTA | 21375 | 0.19753923868608447 | No Hit |
| TAATACTGTCAGGTAAAGATGTCT | 21214 | 0.19605134079469458 | No Hit |
| TAGCTTATCAGACTGATGTTGACT | 20356 | 0.18812204644182157 | No Hit |
| TACCCTGTAGATCCGAATTTGT | 20020 | 0.18501686823370347 | No Hit |
| TGTGAAAGTAGGTAATCGTCAGGCT | 18696 | 0.17278098743742856 | No Hit |
| ACGTCGTGGAGGACCGAACC | 18514 | 0.17109901590803128 | No Hit |
| GTGTGAAAGTAGGTAATCGTCAGGCT | 18210 | 0.16828956895782918 | No Hit |
| TGAGAACTGAATTCCATGGGTTG | 18112 | 0.16738389198046139 | No Hit |
| CCGTGTGAAAGTAGGTAATCGTCAGGCT | 17626 | 0.162892473500862 | No Hit |
| CGTGTGAAAGTAGGTAATCGTCAGGCT | 17200 | 0.15895555112985513 | No Hit |
| GCCCAGGTCGGGGAGCCA | 17100 | 0.15803139094886762 | No Hit |
| TCCTGTACTGAGCTGCCCCGAGT | 17042 | 0.15749537804389482 | No Hit |
| AATGATACGGCGACCACCGA | 16501 | 0.15249567146475232 | No Hit |
| TGAGGTAGTAGATTGTATAGT | 16422 | 0.15176558492177214 | No Hit |
| CCCCCCACTGCTAAATTTGACTGGCTA | 16064 | 0.1484570914738368 | No Hit |
| TCCTGTACTGAGCTGCCCCGA | 15980 | 0.14768079692180727 | No Hit |
| TTCACAGTGGCTAAGTTCTGC | 14872 | 0.13744110211646543 | No Hit |
| TCCTGTACTGAGCTGCCCCGAGA | 14785 | 0.1366370827590063 | No Hit |
| TGAGGTAGTAGGTTGTGTGGTT | 14126 | 0.13054686716629849 | No Hit |
| GCTGGTCCGATGGTAGTGGGTTATCAGAACT | 13407 | 0.12390215546499812 | No Hit |
| TGGACGGAGAACTGATAAGG | 12827 | 0.11854202641527045 | No Hit |
| TGAGGGGCAGAGAGCGAGACTTTT | 12777 | 0.1180799463247767 | No Hit |
| TGGACAACAGGATAATGGCGT | 12768 | 0.11799677190848781 | No Hit |
| CAACGGAATCCCAAAAGCAGCTGT | 12434 | 0.11491007690398947 | No Hit |
| AGGCGCTTGAGAGAACTCGGG | 12106 | 0.11187883151035037 | No Hit |
| TAGCTTATCAGACTGATGTTGAC | 12095 | 0.11177717389044173 | No Hit |
| AGCTCGGTCTGAGGCCCCTCAGT | 12019 | 0.11107481215289121 | No Hit |
| TGAGGTAGTAGATTGTATAGTTT | 11948 | 0.11041865842439007 | No Hit |
| TGAGAACTGAATTCCATAGGCTGT | 11862 | 0.10962388066874079 | No Hit |
| CCCGTGTGAAAGTAGGTAATCGTCAGGCT | 11381 | 0.10517867019819079 | No Hit |
| TCAGTGCATCACAGAACTTTGTTT | 11343 | 0.1048274893294155 | No Hit |
| CCCATAAAGTAGAAAGCACT | 10853 | 0.10029910444257661 | No Hit |
| TTCGACTCCTATCTCCGGCACCA | 10836 | 0.10014199721180873 | No Hit |

## Adapter Content

Produced by FastQC (version 0.11.8)
