# Supplementary material for: Analysis of plasma‐derived small extracellular vesicle characteristics and microRNA cargo following exercise‐induced skeletal muscle damage in men
Source: Physiol Rep. 2024 Sep 20;12(18):e70056. doi: 10.14814/phy2.70056 (PMC11415274; doi:10.14814/phy2.70056)
Supplement: Supplementary file 2 — Data S2: Supporting Information. [file PHY2-12-e70056-s001.zip › Processed Files For Individual Samples/SV18362-0002_S2_R1_001.fastq_CORE_RESULTS_v4.6.2/SV18362-0002_S2_R1_001.fastq/SV18362-0002_S2_R1_001.fastq.clipped.trimmed.filtered_fastqc.zip!/SV18362-0002_S2_R1_001.fastq.clipped.trimmed.filtered_fastqc/fastqc_report.html]

SV18362-0002\_S2\_R1\_001.fastq.clipped.trimmed.filtered.fastq.gz FastQC Report 

FastQC Report

Fri 21 Feb 2020  
SV18362-0002\_S2\_R1\_001.fastq.clipped.trimmed.filtered.fastq.gz

## Summary

- Basic Statistics
- Per base sequence quality
- Per tile sequence quality
- Per sequence quality scores
- Per base sequence content
- Per sequence GC content
- Per base N content
- Sequence Length Distribution
- Sequence Duplication Levels
- Overrepresented sequences
- Adapter Content

## Basic Statistics

| Measure | Value |
| --- | --- |
| Filename | SV18362-0002\_S2\_R1\_001.fastq.clipped.trimmed.filtered.fastq.gz |
| File type | Conventional base calls |
| Encoding | Sanger / Illumina 1.9 |
| Total Sequences | 8902503 |
| Sequences flagged as poor quality | 0 |
| Sequence length | 18-47 |
| %GC | 57 |

## Per base sequence quality

## Per tile sequence quality

## Per sequence quality scores

## Per base sequence content

## Per sequence GC content

## Per base N content

## Sequence Length Distribution

## Sequence Duplication Levels

## Overrepresented sequences

| Sequence | Count | Percentage | Possible Source |
| --- | --- | --- | --- |
| GGCTGGTCCGATGGTAGTGGGTTATCAGAACT | 378941 | 4.256566945273706 | No Hit |
| GCGGGGCGAAGCCAGAGGA | 273177 | 3.0685415101797777 | No Hit |
| TTCAAGTAATCCAGGATAGGCT | 181957 | 2.04388585996545 | No Hit |
| CCCCCCACTGCTAAATTTGACTGGCTT | 145635 | 1.635888244014071 | No Hit |
| GCGGGGCGAAGCCAGAGGAA | 119391 | 1.3410947460506333 | No Hit |
| ACCGGGACGTGGCGGCTGACGGCA | 55072 | 0.6186125407652208 | No Hit |
| CCTCCTGGGAAGTCCTCGTGTTGCACCTCT | 47663 | 0.5353887552747806 | No Hit |
| GCCCGGACCCTGTCGCACCA | 37444 | 0.4206008130522394 | No Hit |
| TGAGGGGCAGAGAGCGAGACTTT | 33865 | 0.38039863620377323 | No Hit |
| TCGAGGAGCTCACAGTCTAGT | 33710 | 0.37865755282531216 | No Hit |
| TGAGGTAGTAGATTGTATAGTT | 33656 | 0.3780509818418483 | No Hit |
| CCGGGACGTGGCGGCTGACGGCA | 33404 | 0.3752203172523503 | No Hit |
| CAACGGAATCCCAAAAGCAGCT | 29814 | 0.3348945796479934 | No Hit |
| GCCGGTCGGGGGACGGATTGGGA | 29683 | 0.333423083373294 | No Hit |
| CCCGGACCCTGTCGCACCA | 28811 | 0.3236280852699516 | No Hit |
| ACCGGGACGTGGCGGCTGA | 27365 | 0.30738546226830815 | No Hit |
| CCCCCACTGCTAAATTTGACTGGCTT | 26228 | 0.2946137732275968 | No Hit |
| CAACGGAATCCCAAAAGCAGCTG | 24666 | 0.277068145891105 | No Hit |
| ACAGGGACAGTCGGGGGC | 24665 | 0.27705691309511493 | No Hit |
| TTCCCGGCTGGTGCACCA | 24640 | 0.27677609319536317 | No Hit |
| TGTAAACATCCCCGACTGGAAGCT | 20070 | 0.22544221552073612 | No Hit |
| ACCACGTGGGAATTCTTGATGCTGTATGT | 17882 | 0.2008648578944596 | No Hit |
| CCGGGACGTGGCGGCTGACGGCAA | 17619 | 0.19791063254907074 | No Hit |
| GATTCCCACTGTCCCTGTCT | 17267 | 0.19395668836056557 | No Hit |
| ATTCCCACTGTCCCTGTCT | 17247 | 0.19373203244076415 | No Hit |
| GCCCCGTCGTGCCCGGACCCT | 16979 | 0.19072164311542494 | No Hit |
| ACCGGGACGTGGCGGCTGACGGCAA | 16970 | 0.1906205479515143 | No Hit |
| TTCCTGAACCGGGACGTGGCGGCTGA | 16527 | 0.18564441932791262 | No Hit |
| CCCCCCACTGCTAAATTTGACTGGCTTT | 16349 | 0.18364498164167986 | No Hit |
| CAGGGACAGTCGGGGGCA | 15886 | 0.17844419709827675 | No Hit |
| TCCTGTACTGAGCTGCCCCGAG | 15131 | 0.16996343612577272 | No Hit |
| AAATGATACGGCGACCACCGA | 14381 | 0.16153883913321906 | No Hit |
| CGTCGGCGGGGGCCCCGGAA | 14339 | 0.16106706170163607 | No Hit |
| ACGAGACCTCAGCCTGCTA | 14233 | 0.15987638532668846 | No Hit |
| CGCCCTCGACCTATTCTC | 14039 | 0.15769722290461458 | No Hit |
| GCGCCCGTAGCTCAACGGATAGAGCACTTGAC | 13941 | 0.15659640889758758 | No Hit |
| GCACCGGTCGGCTCGTCCCTTC | 13562 | 0.15233917921735046 | No Hit |
| TGCCCGGACCCTGTCGCACCA | 13449 | 0.15106987327047236 | No Hit |
| AGACAGCAGGACGGTGGTC | 13226 | 0.1485649597646864 | No Hit |
| GCACCGGTCGGCTCGTCCCTT | 13211 | 0.14839646782483532 | No Hit |
| CCGGGACGTGGCGGCTGA | 13033 | 0.14639703013860259 | No Hit |
| ACCGGGACGTGGCGGCTGACG | 12603 | 0.1415669278628718 | No Hit |
| ACAGGGACAGTCGGGGGCA | 12538 | 0.14083679612351718 | No Hit |
| TAGCTTATCAGACTGATGTTGACT | 12348 | 0.13870256488540356 | No Hit |
| ACGTCGTGGAGGACCGAACC | 12260 | 0.13771407883827727 | No Hit |
| TGAGGTAGTAGGTTGTATAGTT | 12193 | 0.13696148150694248 | No Hit |
| GGCTGGTCCGATGGTAGTGGGTTATCAGAAC | 12171 | 0.1367143599951609 | No Hit |
| TGTAAACATCCTTGACTGGAAGCT | 11935 | 0.13406342014150402 | No Hit |
| GCACCGGTCGGCTCGTCCCT | 11926 | 0.13396232497759336 | No Hit |
| CCGGCGATGCGCTCCTGTCCT | 11907 | 0.13374890185378202 | No Hit |
| ACGAGATTCCCACTGTCCCTGTCT | 11873 | 0.13336698679011957 | No Hit |
| GCGGGGCGAAGCCAGAGG | 11649 | 0.13085084048834356 | No Hit |
| GCCCGGACCCTGTCGCACC | 11573 | 0.12999714799309814 | No Hit |
| TGAGAACTGAATTCCATGGGTT | 11316 | 0.12711031942364973 | No Hit |
| ACCACGTTGGAATTCTTGATGCTGTATGT | 11137 | 0.12509964894142692 | No Hit |
| TCGGCGGGGGCCCCGGAA | 10943 | 0.12292048651935303 | No Hit |
| GCACCGCCGACCGACCCTG | 10400 | 0.11682107829674418 | No Hit |
| CGGGGGACGGATTGGGAA | 10006 | 0.112395356676656 | No Hit |
| GTAGGTAATCGTCAGGCT | 9687 | 0.10881209475582317 | No Hit |
| AAAGTAGGTAATCGTCAGGCT | 9558 | 0.10736306407310393 | No Hit |
| TACCCTGTAGAACCGAATTTGT | 9363 | 0.10517266885503998 | No Hit |
| TCGAGGAGCTCACAGTCTAGTA | 9313 | 0.10461102905553642 | No Hit |
| GGGGATGTAGCTCAAATGGT | 9083 | 0.10202748597781994 | No Hit |
| GCCGGTCGGGGGACGGATTGGGAA | 9057 | 0.10173543328207807 | No Hit |
| GCGGGGCGAAGCCAGAGGAAA | 8974 | 0.10080311121490215 | No Hit |

## Adapter Content

Produced by FastQC (version 0.11.8)
