# Supplementary material for: Analysis of plasma‐derived small extracellular vesicle characteristics and microRNA cargo following exercise‐induced skeletal muscle damage in men
Source: Physiol Rep. 2024 Sep 20;12(18):e70056. doi: 10.14814/phy2.70056 (PMC11415274; doi:10.14814/phy2.70056)
Supplement: Supplementary file 2 — Data S2: Supporting Information. [file PHY2-12-e70056-s001.zip › Processed Files For Individual Samples/SV18362-0004_S4_R1_001.fastq_CORE_RESULTS_v4.6.2.tgz!/SV18362-0004_S4_R1_001.fastq/SV18362-0004_S4_R1_001.fastq.clipped.trimmed.filtered.noRiboRNA_fastqc.zip!/SV18362-0004_S4_R1_001.fastq.clipped.trimmed.filtered.noRiboRNA_fastqc/fastqc_report.html]

SV18362-0004\_S4\_R1\_001.fastq.clipped.trimmed.filtered.noRiboRNA.fastq.gz FastQC Report 

FastQC Report

Fri 21 Feb 2020  
SV18362-0004\_S4\_R1\_001.fastq.clipped.trimmed.filtered.noRiboRNA.fastq.gz

## Summary

- Basic Statistics
- Per base sequence quality
- Per tile sequence quality
- Per sequence quality scores
- Per base sequence content
- Per sequence GC content
- Per base N content
- Sequence Length Distribution
- Sequence Duplication Levels
- Overrepresented sequences
- Adapter Content

## Basic Statistics

| Measure | Value |
| --- | --- |
| Filename | SV18362-0004\_S4\_R1\_001.fastq.clipped.trimmed.filtered.noRiboRNA.fastq.gz |
| File type | Conventional base calls |
| Encoding | Sanger / Illumina 1.9 |
| Total Sequences | 10536304 |
| Sequences flagged as poor quality | 0 |
| Sequence length | 18-47 |
| %GC | 51 |

## Per base sequence quality

## Per tile sequence quality

## Per sequence quality scores

## Per base sequence content

## Per sequence GC content

## Per base N content

## Sequence Length Distribution

## Sequence Duplication Levels

## Overrepresented sequences

| Sequence | Count | Percentage | Possible Source |
| --- | --- | --- | --- |
| GGCTGGTCCGATGGTAGTGGGTTATCAGAACT | 1133549 | 10.758506967908291 | No Hit |
| CCCCCCACTGCTAAATTTGACTGGCTT | 509001 | 4.830925531381782 | No Hit |
| TTCAAGTAATCCAGGATAGGCT | 467963 | 4.441434111999806 | No Hit |
| CCCCCACTGCTAAATTTGACTGGCTT | 88901 | 0.8437588740795634 | No Hit |
| TGAGGTAGTAGATTGTATAGTT | 82292 | 0.7810328935080081 | No Hit |
| TCGAGGAGCTCACAGTCTAGT | 81586 | 0.7743322516130894 | No Hit |
| CAACGGAATCCCAAAAGCAGCT | 75198 | 0.7137037807565157 | No Hit |
| CAACGGAATCCCAAAAGCAGCTG | 64894 | 0.6159085766697696 | No Hit |
| GGCTGGTCCGATGGTAGTGGGTTATCAGAAC | 59518 | 0.5648849919288585 | No Hit |
| TGAGGGGCAGAGAGCGAGACTTT | 56010 | 0.5315905843263444 | No Hit |
| TGTAAACATCCCCGACTGGAAGCT | 47263 | 0.44857285818632414 | No Hit |
| CCCCCCACTGCTAAATTTGACTGGCTTT | 45174 | 0.42874617133294557 | No Hit |
| TGGAATGTAAAGAAGTATGGAG | 45135 | 0.4283760225597136 | No Hit |
| GTAGGTAATCGTCAGGCT | 43506 | 0.41291519303163615 | No Hit |
| TACCCTGTAGAACCGAATTTGT | 39444 | 0.37436277465038975 | No Hit |
| TCCTGTACTGAGCTGCCCCGAG | 37594 | 0.3568044354073307 | No Hit |
| AAAGTAGGTAATCGTCAGGCT | 35444 | 0.33639879790864047 | No Hit |
| TGAGGTAGTAGGTTGTATAGTT | 34988 | 0.33207090456008104 | No Hit |
| TGAGAACTGAATTCCATGGGTT | 34249 | 0.32505705985704286 | No Hit |
| TGAAAGTAGGTAATCGTCAGGCT | 33254 | 0.3156135206425327 | No Hit |
| GAAAGTAGGTAATCGTCAGGCT | 31526 | 0.29921308269009705 | No Hit |
| AGTAGGTAATCGTCAGGCT | 31369 | 0.29772299660298335 | No Hit |
| TCCCTGAGACCCTTTAACCTGT | 30873 | 0.29301546348700647 | No Hit |
| AAGTAGGTAATCGTCAGGCT | 30793 | 0.29225618395217146 | No Hit |
| TGTAAACATCCTTGACTGGAAGCT | 28592 | 0.2713665057500239 | No Hit |
| TGACTAGATCCATACTCATCCA | 26060 | 0.24733530847249663 | No Hit |
| GGCTGGTCCGATGGTAGTGGGTTATCAGAACTT | 25459 | 0.24163122096704878 | No Hit |
| TGAGATCATTGTGAAAGCTGTTT | 24757 | 0.23496854304887177 | No Hit |
| GTGAAAGTAGGTAATCGTCAGGCT | 22385 | 0.21245590484101445 | No Hit |
| GGCTGGTCCGATGGTAGTGGGTTATCAGAACA | 22244 | 0.21111767466086778 | No Hit |
| GAACTATTGACTGGCACTCACAG | 21862 | 0.20749211488203073 | No Hit |
| TCGAGGAGCTCACAGTCTAGTA | 21375 | 0.20287000071372274 | No Hit |
| TAATACTGTCAGGTAAAGATGTCT | 21214 | 0.20134195064986735 | No Hit |
| TAGCTTATCAGACTGATGTTGACT | 20356 | 0.19319867763876214 | No Hit |
| TACCCTGTAGATCCGAATTTGT | 20020 | 0.1900097035924552 | No Hit |
| TGTGAAAGTAGGTAATCGTCAGGCT | 18696 | 0.17744362729093616 | No Hit |
| ACGTCGTGGAGGACCGAACC | 18514 | 0.17571626634918658 | No Hit |
| GTGTGAAAGTAGGTAATCGTCAGGCT | 18210 | 0.17283100411681365 | No Hit |
| TGAGAACTGAATTCCATGGGTTG | 18112 | 0.17190088668664077 | No Hit |
| CCGTGTGAAAGTAGGTAATCGTCAGGCT | 17626 | 0.16728826351251824 | No Hit |
| CGTGTGAAAGTAGGTAATCGTCAGGCT | 17200 | 0.16324509998952194 | No Hit |
| GCCCAGGTCGGGGAGCCA | 17100 | 0.1622960005709782 | No Hit |
| TCCTGTACTGAGCTGCCCCGAGT | 17042 | 0.16174552290822286 | No Hit |
| TGAGGTAGTAGATTGTATAGT | 16422 | 0.15586110651325172 | No Hit |
| CCCCCCACTGCTAAATTTGACTGGCTA | 16064 | 0.15246333059486514 | No Hit |
| TCCTGTACTGAGCTGCCCCGA | 15980 | 0.1516660870832884 | No Hit |
| TTCACAGTGGCTAAGTTCTGC | 14872 | 0.14115006552582385 | No Hit |
| TCCTGTACTGAGCTGCCCCGAGA | 14785 | 0.1403243490316908 | No Hit |
| TGAGGTAGTAGGTTGTGTGGTT | 14126 | 0.1340697838634876 | No Hit |
| GCTGGTCCGATGGTAGTGGGTTATCAGAACT | 13407 | 0.12724575904415816 | No Hit |
| TGGACGGAGAACTGATAAGG | 12827 | 0.12174098241660453 | No Hit |
| TGAGGGGCAGAGAGCGAGACTTTT | 12777 | 0.12126643270733266 | No Hit |
| TGGACAACAGGATAATGGCGT | 12768 | 0.12118101375966372 | No Hit |
| CAACGGAATCCCAAAAGCAGCTGT | 12434 | 0.11801102170172766 | No Hit |
| AGGCGCTTGAGAGAACTCGGG | 12106 | 0.11489797560890422 | No Hit |
| TAGCTTATCAGACTGATGTTGAC | 12095 | 0.11479357467286441 | No Hit |
| AGCTCGGTCTGAGGCCCCTCAGT | 12019 | 0.11407225911477117 | No Hit |
| TGAGGTAGTAGATTGTATAGTTT | 11948 | 0.11339839852760514 | No Hit |
| TGAGAACTGAATTCCATAGGCTGT | 11862 | 0.11258217302765752 | No Hit |
| CCCGTGTGAAAGTAGGTAATCGTCAGGCT | 11381 | 0.10801700482446217 | No Hit |
| TCAGTGCATCACAGAACTTTGTTT | 11343 | 0.10765634704541555 | No Hit |
| CCCATAAAGTAGAAAGCACT | 10853 | 0.10300575989455125 | No Hit |
| TTCGACTCCTATCTCCGGCACCA | 10836 | 0.10284441299339882 | No Hit |
| CAACGGAATCCCAAAAGCAGCTT | 10673 | 0.10129738094117255 | No Hit |

## Adapter Content

Produced by FastQC (version 0.11.8)
