# Supplementary material for: Analysis of plasma‐derived small extracellular vesicle characteristics and microRNA cargo following exercise‐induced skeletal muscle damage in men
Source: Physiol Rep. 2024 Sep 20;12(18):e70056. doi: 10.14814/phy2.70056 (PMC11415274; doi:10.14814/phy2.70056)
Supplement: Supplementary file 2 — Data S2: Supporting Information. [file PHY2-12-e70056-s001.zip › Processed Files For Individual Samples/SV18362-0002_S2_R1_001.fastq_CORE_RESULTS_v4.6.2/SV18362-0002_S2_R1_001.fastq/SV18362-0002_S2_R1_001.fastq.clipped.trimmed.filtered.noRiboRNA_fastqc.zip!/SV18362-0002_S2_R1_001.fastq.clipped.trimmed.filtered.noRiboRNA_fastqc/fastqc_report.html]

SV18362-0002\_S2\_R1\_001.fastq.clipped.trimmed.filtered.noRiboRNA.fastq.gz FastQC Report 

FastQC Report

Fri 21 Feb 2020  
SV18362-0002\_S2\_R1\_001.fastq.clipped.trimmed.filtered.noRiboRNA.fastq.gz

## Summary

- Basic Statistics
- Per base sequence quality
- Per tile sequence quality
- Per sequence quality scores
- Per base sequence content
- Per sequence GC content
- Per base N content
- Sequence Length Distribution
- Sequence Duplication Levels
- Overrepresented sequences
- Adapter Content

## Basic Statistics

| Measure | Value |
| --- | --- |
| Filename | SV18362-0002\_S2\_R1\_001.fastq.clipped.trimmed.filtered.noRiboRNA.fastq.gz |
| File type | Conventional base calls |
| Encoding | Sanger / Illumina 1.9 |
| Total Sequences | 8554326 |
| Sequences flagged as poor quality | 0 |
| Sequence length | 18-47 |
| %GC | 57 |

## Per base sequence quality

## Per tile sequence quality

## Per sequence quality scores

## Per base sequence content

## Per sequence GC content

## Per base N content

## Sequence Length Distribution

## Sequence Duplication Levels

## Overrepresented sequences

| Sequence | Count | Percentage | Possible Source |
| --- | --- | --- | --- |
| GGCTGGTCCGATGGTAGTGGGTTATCAGAACT | 378941 | 4.429817147487715 | No Hit |
| GCGGGGCGAAGCCAGAGGA | 273177 | 3.1934368645758884 | No Hit |
| TTCAAGTAATCCAGGATAGGCT | 181957 | 2.1270758210524128 | No Hit |
| CCCCCCACTGCTAAATTTGACTGGCTT | 145635 | 1.702471942266404 | No Hit |
| GCGGGGCGAAGCCAGAGGAA | 119391 | 1.3956797999047499 | No Hit |
| ACCGGGACGTGGCGGCTGACGGCA | 55072 | 0.6437912232945062 | No Hit |
| CCTCCTGGGAAGTCCTCGTGTTGCACCTCT | 47663 | 0.5571800747364549 | No Hit |
| GCCCGGACCCTGTCGCACCA | 37444 | 0.43772004948139687 | No Hit |
| TGAGGGGCAGAGAGCGAGACTTT | 33865 | 0.3958815691616148 | No Hit |
| TCGAGGAGCTCACAGTCTAGT | 33710 | 0.3940696204470112 | No Hit |
| TGAGGTAGTAGATTGTATAGTT | 33656 | 0.3934383608948268 | No Hit |
| CCGGGACGTGGCGGCTGACGGCA | 33404 | 0.39049248298463257 | No Hit |
| CAACGGAATCCCAAAAGCAGCT | 29814 | 0.3485254127560722 | No Hit |
| GCCGGTCGGGGGACGGATTGGGA | 29683 | 0.3469940238424395 | No Hit |
| CCCGGACCCTGTCGCACCA | 28811 | 0.33680035107383094 | No Hit |
| ACCGGGACGTGGCGGCTGA | 27365 | 0.3198966230653356 | No Hit |
| CCCCCACTGCTAAATTTGACTGGCTT | 26228 | 0.3066051024943403 | No Hit |
| CAACGGAATCCCAAAAGCAGCTG | 24666 | 0.28834533544781904 | No Hit |
| ACAGGGACAGTCGGGGGC | 24665 | 0.2883336454561119 | No Hit |
| TTCCCGGCTGGTGCACCA | 24640 | 0.2880413956634339 | No Hit |
| TGTAAACATCCCCGACTGGAAGCT | 20070 | 0.23461813356189606 | No Hit |
| ACCACGTGGGAATTCTTGATGCTGTATGT | 17882 | 0.20904043170671774 | No Hit |
| CCGGGACGTGGCGGCTGACGGCAA | 17619 | 0.20596596388774524 | No Hit |
| GATTCCCACTGTCCCTGTCT | 17267 | 0.201851086806839 | No Hit |
| ATTCCCACTGTCCCTGTCT | 17247 | 0.20161728697269662 | No Hit |
| GCCCCGTCGTGCCCGGACCCT | 16979 | 0.19848436919518853 | No Hit |
| ACCGGGACGTGGCGGCTGACGGCAA | 16970 | 0.1983791592698244 | No Hit |
| TTCCTGAACCGGGACGTGGCGGCTGA | 16527 | 0.1932004929435703 | No Hit |
| CCCCCCACTGCTAAATTTGACTGGCTTT | 16349 | 0.19111967441970298 | No Hit |
| CAGGGACAGTCGGGGGCA | 15886 | 0.18570720825930645 | No Hit |
| TCCTGTACTGAGCTGCCCCGAG | 15131 | 0.17688126452043096 | No Hit |
| CGTCGGCGGGGGCCCCGGAA | 14339 | 0.167622791088392 | No Hit |
| ACGAGACCTCAGCCTGCTA | 14233 | 0.1663836519674373 | No Hit |
| CGCCCTCGACCTATTCTC | 14039 | 0.16411579357625605 | No Hit |
| GCGCCCGTAGCTCAACGGATAGAGCACTTGAC | 13941 | 0.16297017438895828 | No Hit |
| GCACCGGTCGGCTCGTCCCTTC | 13562 | 0.15853966753195986 | No Hit |
| TGCCCGGACCCTGTCGCACCA | 13449 | 0.1572186984690553 | No Hit |
| AGACAGCAGGACGGTGGTC | 13226 | 0.15461183031836756 | No Hit |
| GCACCGGTCGGCTCGTCCCTT | 13211 | 0.1544364804427608 | No Hit |
| CCGGGACGTGGCGGCTGA | 13033 | 0.15235566191889344 | No Hit |
| ACCGGGACGTGGCGGCTGACG | 12603 | 0.14732896548483188 | No Hit |
| ACAGGGACAGTCGGGGGCA | 12538 | 0.1465691160238691 | No Hit |
| TAGCTTATCAGACTGATGTTGACT | 12348 | 0.14434801759951632 | No Hit |
| ACGTCGTGGAGGACCGAACC | 12260 | 0.14331929832928977 | No Hit |
| TGAGGTAGTAGGTTGTATAGTT | 12193 | 0.14253606888491274 | No Hit |
| GGCTGGTCCGATGGTAGTGGGTTATCAGAAC | 12171 | 0.14227888906735608 | No Hit |
| TGTAAACATCCTTGACTGGAAGCT | 11935 | 0.1395200510244758 | No Hit |
| GCACCGGTCGGCTCGTCCCT | 11926 | 0.13941484109911173 | No Hit |
| CCGGCGATGCGCTCCTGTCCT | 11907 | 0.13919273125667644 | No Hit |
| GCGGGGCGAAGCCAGAGG | 11649 | 0.13617671339623952 | No Hit |
| GCCCGGACCCTGTCGCACC | 11573 | 0.1352882740264984 | No Hit |
| TGAGAACTGAATTCCATGGGTT | 11316 | 0.1322839461577686 | No Hit |
| ACCACGTTGGAATTCTTGATGCTGTATGT | 11137 | 0.13019143764219412 | No Hit |
| TCGGCGGGGGCCCCGGAA | 10943 | 0.1279235792510129 | No Hit |
| GCACCGCCGACCGACCCTG | 10400 | 0.12157591375404678 | No Hit |
| CGGGGGACGGATTGGGAA | 10006 | 0.11697005702144155 | No Hit |
| GTAGGTAATCGTCAGGCT | 9687 | 0.11324094966687032 | No Hit |
| AAAGTAGGTAATCGTCAGGCT | 9558 | 0.11173294073665184 | No Hit |
| TACCCTGTAGAACCGAATTTGT | 9363 | 0.10945339235376346 | No Hit |
| TCGAGGAGCTCACAGTCTAGTA | 9313 | 0.10886889276840747 | No Hit |
| GGGGATGTAGCTCAAATGGT | 9083 | 0.10618019467576989 | No Hit |
| GCCGGTCGGGGGACGGATTGGGAA | 9057 | 0.10587625489138477 | No Hit |
| ACCGGGACGTGGCGGCTG | 8864 | 0.10362008649191065 | No Hit |
| AGCGGGGCGAAGCCAGAGGA | 8709 | 0.10180813777730706 | No Hit |
| TGAAAGTAGGTAATCGTCAGGCT | 8705 | 0.10176137781047857 | No Hit |
| TGAGAACTGAATTCCATAGGCTGT | 8697 | 0.10166785787682163 | No Hit |
| TGCACCGCCGACCGACCCTG | 8693 | 0.10162109790999313 | No Hit |
| GGCTGGTCCGATGGTAGTGGGTTATCAGAACTT | 8639 | 0.10098983835780868 | No Hit |
| AGGCCGGCATTGGGAGTCCATTG | 8578 | 0.10027674886367435 | No Hit |

## Adapter Content

Produced by FastQC (version 0.11.8)
