# Supplementary material for: Analysis of plasma‐derived small extracellular vesicle characteristics and microRNA cargo following exercise‐induced skeletal muscle damage in men
Source: Physiol Rep. 2024 Sep 20;12(18):e70056. doi: 10.14814/phy2.70056 (PMC11415274; doi:10.14814/phy2.70056)
Supplement: Supplementary file 2 — Data S2: Supporting Information. [file PHY2-12-e70056-s001.zip › Processed Files For Individual Samples/SV18362-0004_S4_R1_001.fastq_CORE_RESULTS_v4.6.2.tgz!/SV18362-0004_S4_R1_001.fastq/SV18362-0004_S4_R1_001.fastq.trim_adapter.fastq.tmp_fastqc.zip!/SV18362-0004_S4_R1_001.fastq.trim_adapter.fastq.tmp_fastqc/fastqc_report.html]

SV18362-0004\_S4\_R1\_001.fastq.trim\_adapter.fastq.tmp FastQC Report 

FastQC Report

Fri 21 Feb 2020  
SV18362-0004\_S4\_R1\_001.fastq.trim\_adapter.fastq.tmp

## Summary

- Basic Statistics
- Per base sequence quality
- Per tile sequence quality
- Per sequence quality scores
- Per base sequence content
- Per sequence GC content
- Per base N content
- Sequence Length Distribution
- Sequence Duplication Levels
- Overrepresented sequences
- Adapter Content

## Basic Statistics

| Measure | Value |
| --- | --- |
| Filename | SV18362-0004\_S4\_R1\_001.fastq.trim\_adapter.fastq.tmp |
| File type | Conventional base calls |
| Encoding | Sanger / Illumina 1.9 |
| Total Sequences | 13764143 |
| Sequences flagged as poor quality | 0 |
| Sequence length | 0-47 |
| %GC | 52 |

## Per base sequence quality

## Per tile sequence quality

## Per sequence quality scores

## Per base sequence content

## Per sequence GC content

## Per base N content

## Sequence Length Distribution

## Sequence Duplication Levels

## Overrepresented sequences

| Sequence | Count | Percentage | Possible Source |
| --- | --- | --- | --- |
| GGCTGGTCCGATGGTAGTGGGTTATCAGAACT | 1133549 | 8.235521819266191 | No Hit |
| CCCCCCACTGCTAAATTTGACTGGCTT | 509001 | 3.698021736623922 | No Hit |
| TTCAAGTAATCCAGGATAGGCT | 467963 | 3.399870228026547 | No Hit |
| CCCCCACTGCTAAATTTGACTGGCTT | 88901 | 0.6458883782303046 | No Hit |
| TGAGGTAGTAGATTGTATAGTT | 82292 | 0.5978723121374139 | No Hit |
| TCGAGGAGCTCACAGTCTAGT | 81586 | 0.5927430425563001 | No Hit |
|  | 75332 | 0.5473061417626945 | No Hit |
| CAACGGAATCCCAAAAGCAGCT | 75198 | 0.546332597677894 | No Hit |
| CAACGGAATCCCAAAAGCAGCTG | 64894 | 0.471471416709344 | No Hit |
| GGCTGGTCCGATGGTAGTGGGTTATCAGAAC | 59518 | 0.4324134092474919 | No Hit |
| TGAGGGGCAGAGAGCGAGACTTT | 56010 | 0.40692689693793505 | No Hit |
| TGTAAACATCCCCGACTGGAAGCT | 47263 | 0.34337771701441927 | No Hit |
| CCCCCCACTGCTAAATTTGACTGGCTTT | 45174 | 0.3282006006476393 | No Hit |
| TGGAATGTAAAGAAGTATGGAG | 45135 | 0.32791725572743613 | No Hit |
| GTAGGTAATCGTCAGGCT | 43506 | 0.3160821563681807 | No Hit |
| TACCCTGTAGAACCGAATTTGT | 39444 | 0.2865706931408661 | No Hit |
| TCCTGTACTGAGCTGCCCCGAG | 37594 | 0.27312997256712607 | No Hit |
| AAAGTAGGTAATCGTCAGGCT | 35444 | 0.25750967568413086 | No Hit |
| TGAGGTAGTAGGTTGTATAGTT | 34988 | 0.25419671969406304 | No Hit |
| TGAGAACTGAATTCCATGGGTT | 34249 | 0.24882769671893118 | No Hit |
| TGAAAGTAGGTAATCGTCAGGCT | 33254 | 0.24159876862656832 | No Hit |
| AAATGATACGGCGACCACCGA | 32761 | 0.2380169982250257 | No Hit |
| GAAAGTAGGTAATCGTCAGGCT | 31526 | 0.22904440908525872 | No Hit |
| AGTAGGTAATCGTCAGGCT | 31369 | 0.22790376415008184 | No Hit |
| TCCCTGAGACCCTTTAACCTGT | 30873 | 0.22430019798544668 | No Hit |
| AAGTAGGTAATCGTCAGGCT | 30793 | 0.22371897763631196 | No Hit |
| TGTAAACATCCTTGACTGGAAGCT | 28592 | 0.20772815278074344 | No Hit |
| TGACTAGATCCATACTCATCCA | 26060 | 0.18933252873063003 | No Hit |
| GGCTGGTCCGATGGTAGTGGGTTATCAGAACTT | 25459 | 0.18496611085775555 | No Hit |
| TGAGATCATTGTGAAAGCTGTTT | 24757 | 0.17986590229409852 | No Hit |
| GTGAAAGTAGGTAATCGTCAGGCT | 22385 | 0.16263271894225453 | No Hit |
| GGCTGGTCCGATGGTAGTGGGTTATCAGAACA | 22244 | 0.16160831807690462 | No Hit |
| GAACTATTGACTGGCACTCACAG | 21862 | 0.1588329909097864 | No Hit |
| TCGAGGAGCTCACAGTCTAGTA | 21375 | 0.15529481203442888 | No Hit |
| TAATACTGTCAGGTAAAGATGTCT | 21214 | 0.15412510608179528 | No Hit |
| TAGCTTATCAGACTGATGTTGACT | 20356 | 0.14789151783732557 | No Hit |
| TACCCTGTAGATCCGAATTTGT | 20020 | 0.14545039237095983 | No Hit |
| TGTGAAAGTAGGTAATCGTCAGGCT | 18696 | 0.13583119559278045 | No Hit |
| AGGCCGAGGACTTACC | 18515 | 0.13451618455286318 | No Hit |
| ACGTCGTGGAGGACCGAACC | 18514 | 0.134508919298499 | No Hit |
| GTGTGAAAGTAGGTAATCGTCAGGCT | 18210 | 0.13230028197178711 | No Hit |
| TGAGAACTGAATTCCATGGGTTG | 18112 | 0.1315882870440971 | No Hit |
| CCGTGTGAAAGTAGGTAATCGTCAGGCT | 17626 | 0.12805737342310378 | No Hit |
| CGTGTGAAAGTAGGTAATCGTCAGGCT | 17200 | 0.12496237506396148 | No Hit |
| GCCCAGGTCGGGGAGCCA | 17100 | 0.1242358496275431 | No Hit |
| TCCTGTACTGAGCTGCCCCGAGT | 17042 | 0.12381446487442045 | No Hit |
| AATGATACGGCGACCACCGA | 16501 | 0.119883962263397 | No Hit |
| TGAGGTAGTAGATTGTATAGT | 16422 | 0.11931000716862648 | No Hit |
| CCCCCCACTGCTAAATTTGACTGGCTA | 16064 | 0.11670904610624867 | No Hit |
| TCCTGTACTGAGCTGCCCCGA | 15980 | 0.11609876473965723 | No Hit |
| GGTAATCGTCAGGCT | 15670 | 0.11384653588676025 | No Hit |
| TAGGTAATCGTCAGGCT | 14902 | 0.10826682053506709 | No Hit |
| TTCACAGTGGCTAAGTTCTGC | 14872 | 0.10804886290414159 | No Hit |
| TCCTGTACTGAGCTGCCCCGAGA | 14785 | 0.1074167857744576 | No Hit |
| TGAGGTAGTAGGTTGTGTGGTT | 14126 | 0.10262898314846046 | No Hit |

## Adapter Content

Produced by FastQC (version 0.11.8)
