# Supplementary material for: Analysis of plasma‐derived small extracellular vesicle characteristics and microRNA cargo following exercise‐induced skeletal muscle damage in men
Source: Physiol Rep. 2024 Sep 20;12(18):e70056. doi: 10.14814/phy2.70056 (PMC11415274; doi:10.14814/phy2.70056)
Supplement: Supplementary file 2 — Data S2: Supporting Information. [file PHY2-12-e70056-s001.zip › Processed Files For Individual Samples/SV18362-0003_S3_R1_001.fastq_CORE_RESULTS_v4.6.2.tgz!/SV18362-0003_S3_R1_001.fastq/SV18362-0003_S3_R1_001.fastq.clipped.trimmed.filtered_fastqc.zip!/SV18362-0003_S3_R1_001.fastq.clipped.trimmed.filtered_fastqc/fastqc_report.html]

SV18362-0003\_S3\_R1\_001.fastq.clipped.trimmed.filtered.fastq.gz FastQC Report 

FastQC Report

Fri 21 Feb 2020  
SV18362-0003\_S3\_R1\_001.fastq.clipped.trimmed.filtered.fastq.gz

## Summary

- Basic Statistics
- Per base sequence quality
- Per tile sequence quality
- Per sequence quality scores
- Per base sequence content
- Per sequence GC content
- Per base N content
- Sequence Length Distribution
- Sequence Duplication Levels
- Overrepresented sequences
- Adapter Content

## Basic Statistics

| Measure | Value |
| --- | --- |
| Filename | SV18362-0003\_S3\_R1\_001.fastq.clipped.trimmed.filtered.fastq.gz |
| File type | Conventional base calls |
| Encoding | Sanger / Illumina 1.9 |
| Total Sequences | 9337638 |
| Sequences flagged as poor quality | 0 |
| Sequence length | 18-47 |
| %GC | 52 |

## Per base sequence quality

## Per tile sequence quality

## Per sequence quality scores

## Per base sequence content

## Per sequence GC content

## Per base N content

## Sequence Length Distribution

## Sequence Duplication Levels

## Overrepresented sequences

| Sequence | Count | Percentage | Possible Source |
| --- | --- | --- | --- |
| GGCTGGTCCGATGGTAGTGGGTTATCAGAACT | 1017529 | 10.897070543964116 | No Hit |
| CCCCCCACTGCTAAATTTGACTGGCTT | 425782 | 4.5598469334536205 | No Hit |
| TTCAAGTAATCCAGGATAGGCT | 224684 | 2.4062187889485545 | No Hit |
| CCCCCACTGCTAAATTTGACTGGCTT | 74531 | 0.7981782973381492 | No Hit |
| TGAGGTAGTAGATTGTATAGTT | 48707 | 0.5216201356274467 | No Hit |
| TGAGGGGCAGAGAGCGAGACTTT | 47081 | 0.504206738363599 | No Hit |
| TCGAGGAGCTCACAGTCTAGT | 45465 | 0.4869004345638587 | No Hit |
| CCCCCCACTGCTAAATTTGACTGGCTTT | 44037 | 0.47160748788933565 | No Hit |
| GGCTGGTCCGATGGTAGTGGGTTATCAGAAC | 43593 | 0.4668525380829713 | No Hit |
| CAACGGAATCCCAAAAGCAGCTG | 39712 | 0.42528956466292656 | No Hit |
| CAACGGAATCCCAAAAGCAGCT | 37883 | 0.4057021700777006 | No Hit |
| TCCTGTACTGAGCTGCCCCGAG | 31522 | 0.3375800175590444 | No Hit |
| AAATGATACGGCGACCACCGA | 27506 | 0.294571282373551 | No Hit |
| TGTAAACATCCCCGACTGGAAGCT | 26691 | 0.2858431650488057 | No Hit |
| GTAGGTAATCGTCAGGCT | 26405 | 0.28278029197533683 | No Hit |
| AAAGTAGGTAATCGTCAGGCT | 21927 | 0.234823838748086 | No Hit |
| TGAAAGTAGGTAATCGTCAGGCT | 21916 | 0.23470603593756792 | No Hit |
| GGCTGGTCCGATGGTAGTGGGTTATCAGAACTT | 21264 | 0.22772354207777173 | No Hit |
| TGAGAACTGAATTCCATGGGTT | 21138 | 0.22637416443001968 | No Hit |
| GAAAGTAGGTAATCGTCAGGCT | 19303 | 0.20672251376632933 | No Hit |
| AAGTAGGTAATCGTCAGGCT | 19158 | 0.20516965853677344 | No Hit |
| AGTAGGTAATCGTCAGGCT | 18027 | 0.19305738774623732 | No Hit |
| TGAGGTAGTAGGTTGTATAGTT | 15714 | 0.16828666949821786 | No Hit |
| TGTAAACATCCTTGACTGGAAGCT | 15684 | 0.16796538910589595 | No Hit |
| CGGAATGTAGCGCAGTTGGTAGC | 15566 | 0.16670168622942974 | No Hit |
| GGCTGGTCCGATGGTAGTGGGTTATCAGAACA | 15247 | 0.16328540472440675 | No Hit |
| TCCTGTACTGAGCTGCCCCGA | 14715 | 0.15758803243389816 | No Hit |
| CCCCCCACTGCTAAATTTGACTGGCTA | 14711 | 0.15754519504825526 | No Hit |
| GTGAAAGTAGGTAATCGTCAGGCT | 14345 | 0.1536255742619279 | No Hit |
| GCGCCCGTAGCTCAACGGATAGAGCACTTGAC | 14187 | 0.1519334975290325 | No Hit |
| TAGCTTATCAGACTGATGTTGACT | 13064 | 0.13990690150978224 | No Hit |
| TCGAGGAGCTCACAGTCTAGTA | 12958 | 0.1387717107902448 | No Hit |
| TCCTGTACTGAGCTGCCCCGAGA | 12894 | 0.13808631261995807 | No Hit |
| GTGTGAAAGTAGGTAATCGTCAGGCT | 12733 | 0.13636210784783048 | No Hit |
| AATGATACGGCGACCACCGA | 12226 | 0.13093246921759014 | No Hit |
| TGAGAACTGAATTCCATAGGCTGT | 11857 | 0.12698072039203062 | No Hit |
| CGTGTGAAAGTAGGTAATCGTCAGGCT | 11670 | 0.12497807261322404 | No Hit |
| CCGTGTGAAAGTAGGTAATCGTCAGGCT | 11600 | 0.12422841836447289 | No Hit |
| GCCCAGGTCGGGGAGCCA | 11425 | 0.12235428274259506 | No Hit |
| TGTGAAAGTAGGTAATCGTCAGGCT | 11371 | 0.12177597803641564 | No Hit |
| TCCTGTACTGAGCTGCCCCGAGT | 10969 | 0.117470820779302 | No Hit |
| TGAGGGGCAGAGAGCGAGACTTTT | 10933 | 0.1170852843085157 | No Hit |
| GCTGGTCCGATGGTAGTGGGTTATCAGAACT | 10642 | 0.11396886450299316 | No Hit |
| TGAGAACTGAATTCCATGGGTTG | 10616 | 0.11369042149631417 | No Hit |
| TGAACTGAAGAGTTTGATCCTGGCTC | 10458 | 0.11199834476341874 | No Hit |
| GCGGAAGTAGCTCAGTTGATAGAGC | 9473 | 0.10144963854884928 | No Hit |

## Adapter Content

Produced by FastQC (version 0.11.8)
