# Supplementary material for: Analysis of plasma‐derived small extracellular vesicle characteristics and microRNA cargo following exercise‐induced skeletal muscle damage in men
Source: Physiol Rep. 2024 Sep 20;12(18):e70056. doi: 10.14814/phy2.70056 (PMC11415274; doi:10.14814/phy2.70056)
Supplement: Supplementary file 2 — Data S2: Supporting Information. [file PHY2-12-e70056-s001.zip › Processed Files For Individual Samples/SV18362-0003_S3_R1_001.fastq_CORE_RESULTS_v4.6.2.tgz!/SV18362-0003_S3_R1_001.fastq/SV18362-0003_S3_R1_001.fastq.clipped.trimmed.filtered.noRiboRNA_fastqc.zip!/SV18362-0003_S3_R1_001.fastq.clipped.trimmed.filtered.noRiboRNA_fastqc/fastqc_report.html]

SV18362-0003\_S3\_R1\_001.fastq.clipped.trimmed.filtered.noRiboRNA.fastq.gz FastQC Report 

FastQC Report

Fri 21 Feb 2020  
SV18362-0003\_S3\_R1\_001.fastq.clipped.trimmed.filtered.noRiboRNA.fastq.gz

## Summary

- Basic Statistics
- Per base sequence quality
- Per tile sequence quality
- Per sequence quality scores
- Per base sequence content
- Per sequence GC content
- Per base N content
- Sequence Length Distribution
- Sequence Duplication Levels
- Overrepresented sequences
- Adapter Content

## Basic Statistics

| Measure | Value |
| --- | --- |
| Filename | SV18362-0003\_S3\_R1\_001.fastq.clipped.trimmed.filtered.noRiboRNA.fastq.gz |
| File type | Conventional base calls |
| Encoding | Sanger / Illumina 1.9 |
| Total Sequences | 9037792 |
| Sequences flagged as poor quality | 0 |
| Sequence length | 18-47 |
| %GC | 52 |

## Per base sequence quality

## Per tile sequence quality

## Per sequence quality scores

## Per base sequence content

## Per sequence GC content

## Per base N content

## Sequence Length Distribution

## Sequence Duplication Levels

## Overrepresented sequences

| Sequence | Count | Percentage | Possible Source |
| --- | --- | --- | --- |
| GGCTGGTCCGATGGTAGTGGGTTATCAGAACT | 1017529 | 11.258601658458172 | No Hit |
| CCCCCCACTGCTAAATTTGACTGGCTT | 425782 | 4.711128558833839 | No Hit |
| TTCAAGTAATCCAGGATAGGCT | 224684 | 2.486049690012782 | No Hit |
| CCCCCACTGCTAAATTTGACTGGCTT | 74531 | 0.8246593858322918 | No Hit |
| TGAGGTAGTAGATTGTATAGTT | 48707 | 0.5389258792413014 | No Hit |
| TGAGGGGCAGAGAGCGAGACTTT | 47081 | 0.520934759286339 | No Hit |
| TCGAGGAGCTCACAGTCTAGT | 45465 | 0.5030542858255644 | No Hit |
| CCCCCCACTGCTAAATTTGACTGGCTTT | 44037 | 0.48725396645552366 | No Hit |
| GGCTGGTCCGATGGTAGTGGGTTATCAGAAC | 43593 | 0.4823412621135782 | No Hit |
| CAACGGAATCCCAAAAGCAGCTG | 39712 | 0.43939935771923055 | No Hit |
| CAACGGAATCCCAAAAGCAGCT | 37883 | 0.41916211393225244 | No Hit |
| TCCTGTACTGAGCTGCCCCGAG | 31522 | 0.3487798789792905 | No Hit |
| TGTAAACATCCCCGACTGGAAGCT | 26691 | 0.29532655763708654 | No Hit |
| GTAGGTAATCGTCAGGCT | 26405 | 0.2921620679033109 | No Hit |
| AAAGTAGGTAATCGTCAGGCT | 21927 | 0.242614567805942 | No Hit |
| TGAAAGTAGGTAATCGTCAGGCT | 21916 | 0.24249285666233522 | No Hit |
| GGCTGGTCCGATGGTAGTGGGTTATCAGAACTT | 21264 | 0.2352787052412802 | No Hit |
| TGAGAACTGAATTCCATGGGTT | 21138 | 0.23388455941451186 | No Hit |
| GAAAGTAGGTAATCGTCAGGCT | 19303 | 0.21358092773102103 | No Hit |
| AAGTAGGTAATCGTCAGGCT | 19158 | 0.2119765535652956 | No Hit |
| AGTAGGTAATCGTCAGGCT | 18027 | 0.1994624350726372 | No Hit |
| TGAGGTAGTAGGTTGTATAGTT | 15714 | 0.17386990096696184 | No Hit |
| TGTAAACATCCTTGACTGGAAGCT | 15684 | 0.17353796148439796 | No Hit |
| CGGAATGTAGCGCAGTTGGTAGC | 15566 | 0.17223233285298 | No Hit |
| GGCTGGTCCGATGGTAGTGGGTTATCAGAACA | 15247 | 0.16870270968838405 | No Hit |
| TCCTGTACTGAGCTGCCCCGA | 14715 | 0.16281631619758455 | No Hit |
| CCCCCCACTGCTAAATTTGACTGGCTA | 14711 | 0.16277205759990934 | No Hit |
| GTGAAAGTAGGTAATCGTCAGGCT | 14345 | 0.15872239591263 | No Hit |
| GCGCCCGTAGCTCAACGGATAGAGCACTTGAC | 14187 | 0.1569741813044602 | No Hit |
| TAGCTTATCAGACTGATGTTGACT | 13064 | 0.14454858000715218 | No Hit |
| TCGAGGAGCTCACAGTCTAGTA | 12958 | 0.14337572716875982 | No Hit |
| TCCTGTACTGAGCTGCCCCGAGA | 12894 | 0.14266758960595685 | No Hit |
| GTGTGAAAGTAGGTAATCGTCAGGCT | 12733 | 0.14088618104953068 | No Hit |
| TGAGAACTGAATTCCATAGGCTGT | 11857 | 0.13119354815866532 | No Hit |
| CGTGTGAAAGTAGGTAATCGTCAGGCT | 11670 | 0.12912445871735043 | No Hit |
| CCGTGTGAAAGTAGGTAATCGTCAGGCT | 11600 | 0.1283499332580347 | No Hit |
| GCCCAGGTCGGGGAGCCA | 11425 | 0.1264136196097454 | No Hit |
| TGTGAAAGTAGGTAATCGTCAGGCT | 11371 | 0.1258161285411304 | No Hit |
| TCCTGTACTGAGCTGCCCCGAGT | 10969 | 0.12136813947477437 | No Hit |
| TGAGGGGCAGAGAGCGAGACTTTT | 10933 | 0.1209698120956977 | No Hit |
| GCTGGTCCGATGGTAGTGGGTTATCAGAACT | 10642 | 0.11774999911482804 | No Hit |
| TGAGAACTGAATTCCATGGGTTG | 10616 | 0.11746231822993936 | No Hit |
| TGAACTGAAGAGTTTGATCCTGGCTC | 10458 | 0.11571410362176957 | No Hit |
| GCGGAAGTAGCTCAGTTGATAGAGC | 9473 | 0.1048154239442554 | No Hit |

## Adapter Content

Produced by FastQC (version 0.11.8)
