# Supplementary material for: Analysis of plasma‐derived small extracellular vesicle characteristics and microRNA cargo following exercise‐induced skeletal muscle damage in men
Source: Physiol Rep. 2024 Sep 20;12(18):e70056. doi: 10.14814/phy2.70056 (PMC11415274; doi:10.14814/phy2.70056)
Supplement: Supplementary file 2 — Data S2: Supporting Information. [file PHY2-12-e70056-s001.zip › Processed Files For Individual Samples/SV18362-0003_S3_R1_001.fastq_CORE_RESULTS_v4.6.2.tgz!/SV18362-0003_S3_R1_001.fastq/SV18362-0003_S3_R1_001.fastq.trim_adapter.fastq.tmp_fastqc.zip!/SV18362-0003_S3_R1_001.fastq.trim_adapter.fastq.tmp_fastqc/fastqc_report.html]

SV18362-0003\_S3\_R1\_001.fastq.trim\_adapter.fastq.tmp FastQC Report 

FastQC Report

Fri 21 Feb 2020  
SV18362-0003\_S3\_R1\_001.fastq.trim\_adapter.fastq.tmp

## Summary

- Basic Statistics
- Per base sequence quality
- Per tile sequence quality
- Per sequence quality scores
- Per base sequence content
- Per sequence GC content
- Per base N content
- Sequence Length Distribution
- Sequence Duplication Levels
- Overrepresented sequences
- Adapter Content

## Basic Statistics

| Measure | Value |
| --- | --- |
| Filename | SV18362-0003\_S3\_R1\_001.fastq.trim\_adapter.fastq.tmp |
| File type | Conventional base calls |
| Encoding | Sanger / Illumina 1.9 |
| Total Sequences | 11882909 |
| Sequences flagged as poor quality | 0 |
| Sequence length | 0-47 |
| %GC | 53 |

## Per base sequence quality

## Per tile sequence quality

## Per sequence quality scores

## Per base sequence content

## Per sequence GC content

## Per base N content

## Sequence Length Distribution

## Sequence Duplication Levels

## Overrepresented sequences

| Sequence | Count | Percentage | Possible Source |
| --- | --- | --- | --- |
| GGCTGGTCCGATGGTAGTGGGTTATCAGAACT | 1017529 | 8.562962150093046 | No Hit |
| CCCCCCACTGCTAAATTTGACTGGCTT | 425782 | 3.583146180787886 | No Hit |
| TTCAAGTAATCCAGGATAGGCT | 224684 | 1.8908164659007318 | No Hit |
| CCCCCACTGCTAAATTTGACTGGCTT | 74531 | 0.6272117374626028 | No Hit |
| TGAGGTAGTAGATTGTATAGTT | 48707 | 0.40989121434827114 | No Hit |
| TGAGGGGCAGAGAGCGAGACTTT | 47081 | 0.39620769628043107 | No Hit |
| TCGAGGAGCTCACAGTCTAGT | 45465 | 0.38260833269025285 | No Hit |
| CCCCCCACTGCTAAATTTGACTGGCTTT | 44037 | 0.37059107328012025 | No Hit |
| GGCTGGTCCGATGGTAGTGGGTTATCAGAAC | 43593 | 0.3668546144719277 | No Hit |
|  | 42498 | 0.3576396991679394 | No Hit |
| CAACGGAATCCCAAAAGCAGCTG | 39712 | 0.33419426169130806 | No Hit |
| CAACGGAATCCCAAAAGCAGCT | 37883 | 0.31880240772692947 | No Hit |
| TCCTGTACTGAGCTGCCCCGAG | 31522 | 0.2652717444861355 | No Hit |
| AAATGATACGGCGACCACCGA | 27506 | 0.231475306257079 | No Hit |
| TGTAAACATCCCCGACTGGAAGCT | 26691 | 0.22461671632762653 | No Hit |
| GTAGGTAATCGTCAGGCT | 26405 | 0.2222098982664935 | No Hit |
| AAAGTAGGTAATCGTCAGGCT | 21927 | 0.18452552316945287 | No Hit |
| TGAAAGTAGGTAATCGTCAGGCT | 21916 | 0.18443295324402467 | No Hit |
| GGCTGGTCCGATGGTAGTGGGTTATCAGAACTT | 21264 | 0.17894608130046272 | No Hit |
| TGAGAACTGAATTCCATGGGTT | 21138 | 0.17788573488192158 | No Hit |
| GCGCCCGTAGCTCAAC | 20864 | 0.17557990219398298 | No Hit |
| GAAAGTAGGTAATCGTCAGGCT | 19303 | 0.1624433882309458 | No Hit |
| AAGTAGGTAATCGTCAGGCT | 19158 | 0.1612231483048469 | No Hit |
| AGTAGGTAATCGTCAGGCT | 18027 | 0.15170527688127544 | No Hit |
| TGAGGTAGTAGGTTGTATAGTT | 15714 | 0.13224034619805639 | No Hit |
| TGTAAACATCCTTGACTGGAAGCT | 15684 | 0.1319878827650704 | No Hit |
| CGGAATGTAGCGCAGTTGGTAGC | 15566 | 0.1309948599286589 | No Hit |
| GGCTGGTCCGATGGTAGTGGGTTATCAGAACA | 15247 | 0.1283103320912413 | No Hit |
| CGCGGGGTGGAGC | 14780 | 0.1243803179844262 | No Hit |
| TCCTGTACTGAGCTGCCCCGA | 14715 | 0.12383331387962324 | No Hit |
| CCCCCCACTGCTAAATTTGACTGGCTA | 14711 | 0.12379965208855845 | No Hit |
| GTGAAAGTAGGTAATCGTCAGGCT | 14345 | 0.1207195982061295 | No Hit |
| GCGCCCGTAGCTCAACGGATAGAGCACTTGAC | 14187 | 0.11938995745907 | No Hit |
| AGGCCGAGGACTTACC | 13076 | 0.11004039499082252 | No Hit |
| TAGCTTATCAGACTGATGTTGACT | 13064 | 0.10993940961762814 | No Hit |
| TCGAGGAGCTCACAGTCTAGTA | 12958 | 0.109047372154411 | No Hit |
| TCCTGTACTGAGCTGCCCCGAGA | 12894 | 0.10850878349737425 | No Hit |
| GTGTGAAAGTAGGTAATCGTCAGGCT | 12733 | 0.10715389640701616 | No Hit |
| AATGATACGGCGACCACCGA | 12226 | 0.1028872643895531 | No Hit |

## Adapter Content

Produced by FastQC (version 0.11.8)
